# Phenotypic Characterization by Mass Cytometry of the Microenvironment in Ovarian Cancer and Impact of Tumor Dissociation Methods

**DOI:** 10.3390/cancers13040755

**Published:** 2021-02-11

**Authors:** Shamundeeswari Anandan, Liv Cecilie V. Thomsen, Stein-Erik Gullaksen, Tamim Abdelaal, Katrin Kleinmanns, Jørn Skavland, Geir Bredholt, Bjørn Tore Gjertsen, Emmet McCormack, Line Bjørge

**Affiliations:** 1 Centre for Cancer Biomarkers, Department of Clinical Science, University of Bergen, 5021 Bergen, Norway; Shamundeeswari.Anandan@uib.no (S.A.); liv.vestrheim@uib.no (L.C.V.T.); stein.gullaksen@uib.no (S.-E.G.); katrin.kleinmanns@uib.no (K.K.); Jorn.Skavland@uib.no (J.S.); Geir.Bredholt@uib.no (G.B.); bjorn.gjertsen@uib.no (B.T.G.); Emmet.Mc.Cormack@uib.no (E.M.); 2 Department of Obstetrics and Gynecology, Haukeland University Hospital, 5021 Bergen, Norway; 3 Delft Bioinformatics Lab, Delft University of Technology, 2600 AA Delft, The Netherlands; T.R.M.Abdelaal-1@tudelft.nl; 4 Department of Internal Medicine, Hematology Section, Haukeland University Hospital, 5021 Bergen, Norway; 5 Centre for Pharmacy, Department of Clinical Science, The University of Bergen, Jonas Lies vei 65, 5021 Bergen, Norway

**Keywords:** high-grade serous ovarian cancer (HGSOC), single-cell mass cytometry, Cytometry by Time-of-Flight (CyTOF), tumor microenvironment (TME), cell expression profile, tumor dissociation, characterization

## Abstract

**Simple Summary:**

High-grade serous ovarian cancer (HGSOC) is the deadliest gynecological malignancy. Despite increasing research on HGSOC, biomarkers for individualized selection of therapy are scarce. In this study, we develop a multiparametric mass cytometry antibody panel to identify differences in the cellular composition of the microenvironment of tumor tissues dissociated to single-cell suspensions. We also investigate how dissociation methods impact results. Application of our antibody panel to HGSOC tissues showed its ability to identify established main cell subsets and subpopulations of these cells. Comparisons between dissociation methods revealed differences in cell fractions for one immune, two stromal, and three tumor cell subpopulations, while functional marker expression was not affected by the dissociation method. The interpatient disparities identified in the tumor microenvironment were more significant than those identified between differently dissociated tissues from one patient, indicating that the panel facilitates the mapping of individual tumor microenvironments in HGSOC patients.

**Abstract:**

Improved molecular dissection of the tumor microenvironment (TME) holds promise for treating high-grade serous ovarian cancer (HGSOC), a gynecological malignancy with high mortality. Reliable disease-related biomarkers are scarce, but single-cell mapping of the TME could identify patient-specific prognostic differences. To avoid technical variation effects, however, tissue dissociation effects on single cells must be considered. We present a novel Cytometry by Time-of-Flight antibody panel for single-cell suspensions to identify individual TME profiles of HGSOC patients and evaluate the effects of dissociation methods on results. The panel was developed utilizing cell lines, healthy donor blood, and stem cells and was applied to HGSOC tissues dissociated by six methods. Data were analyzed using Cytobank and X-shift and illustrated by t-distributed stochastic neighbor embedding plots, heatmaps, and stacked bar and error plots. The panel distinguishes the main cellular subsets and subpopulations, enabling characterization of individual TME profiles. The dissociation method affected some immune (*n* = 1), stromal (*n* = 2), and tumor (*n* = 3) subsets, while functional marker expressions remained comparable. In conclusion, the panel can identify subsets of the HGSOC TME and can be used for in-depth profiling. This panel represents a promising profiling tool for HGSOC when tissue handling is considered.

## 1. Introduction

The five-year survival rate is low for high-grade serous ovarian cancer (HGSOC), the most common epithelial ovarian cancer subtype, and long-term survival rates have improved only modestly over the last three decades despite increasingly aggressive surgical and chemotherapeutic approaches [1].

Like other malignant tumors, HGSOC consists not only of aggregates of heterogeneous populations of cancer cells but also of a variety of stromal and infiltrating immune cells, extracellular matrix proteins, and secreted molecules, collectively known as the tumor microenvironment (TME) [2,3]. This complex and dynamic interactive entity contributes to tumor growth through a reciprocal interplay between cancer and host cells [2,4].

The characterization of the composition, organization, and functionality of the HGSOC TME using sensitive tools [5] is fundamental for developing new management strategies and improving survival rates [6]. Traditionally, immunohistochemistry analysis and large-scale high-throughput omics technologies have been used for HGSOC TME characterization on the single-cell level [7,8,9,10,11]. Some components of the TME have been described using various methods, such as gene expression analyses of mRNA and microRNA, genetic mapping of promoter methylation and DNA copy numbers, and immune cell-focused analysis of the T cell receptor. However, a global depiction of the constituent cells of the TME is still required [6,7,8,9,10]. Single-cell Cytometry by Time-of-Flight (CyTOF), a relatively new method for single-cell profiling, utilizes stable isotopes combined with monoclonal antibodies as reporters. The method enables detailed phenotypic characterization and simultaneous detection of over 40 parameters per cell at a single-cell level [12]. However, few studies using this method of single-cell phenotyping of HGSOC tissues have been published. Those that are available describe novel cellular phenotypes associated with a specific immunological TME [13,14,15,16,17]. Gonzalez et al. applied a CyTOF panel of 41 antibody markers to dissociated tumors. Four of the markers were used to identify viable tumor cells and exclude stromal, immune, and blood vessel cells, and the remaining markers were used to interrogate HGSOC tumor cell biology [13]. Toker et al. and Kverneland et al. applied the CyTOF method to dissociated HGSOC tissues, focusing exclusively on the different T cell subsets of the ovarian cancer TME [14,15], while Casado et al. applied a new analysis tool combined with a HGSOC mass cytometry panel, which enabled them to identify different subsets of cells, mainly tumor and immune cells, in dissociated tissues [16]. Currently, except for Casado et al.’s study, the published research has been largely restricted to comparisons within tumor or immune cell subsets, while a more global characterization of all major cell subsets of the TME is lacking.

Due to the underlying complexity of data generated through CyTOF analyses, adequate attention to sample preparation is crucial to avoid introducing technically induced variation that may obscure biological insights. A common goal when preparing single-cell suspensions from solid tumors is to preserve cell viability and cellular diversity [18]. However, the extent to which the expression of certain markers is disrupted by the process of common tissue dissociation methods is incompletely understood. To interpret in-depth single-cell phenotyping results, it is important to establish a comprehensive understanding of the effects of sample conditioning. This information should also be included in the standard protocols for tissue dissociation to avoid unacknowledged methodological bias introduced by the sample treatment. Systematic comparisons of the cellular effects of tissue preparation techniques have previously been carried out in CyTOF analyses of gliomas, melanomas, small cell lung cancer, and tonsil tissues but not in HGSOC [18,19].

In this study, we present a novel TME-based 35-marker CyTOF panel to identify patient-specific tumor phenotypes. The CyTOF panel is optimized for single-cell suspensions of HGSOC tissues and delineates the main cell subsets of the TME: tumor cells, the immune cell subset, and the stromal niche. By applying the CyTOF panel to patient material, we evaluated the effects of six different tissue dissociation methods on cellular expression and demonstrated the impact of different techniques on antigen expression profiles. This is an important consideration when single-cell tumor suspensions are used to examine the HGSOC TME. However, we demonstrate how interpatient differences in the TME can be identified independent of the dissociation technique applied to the tissues. As the panel can be used to identify the main populations of immune, stromal, and tumor cells in the TME, as well as potential new cellular subsets within specific populations, its application, and the combination of the results with clinical data could provide insights into how differences in the TME composition impact clinical outcomes.

## 2. Results

### 2.1. Development of a Novel HGSOC TME-Based CyTOF Panel

**Figure 1:**
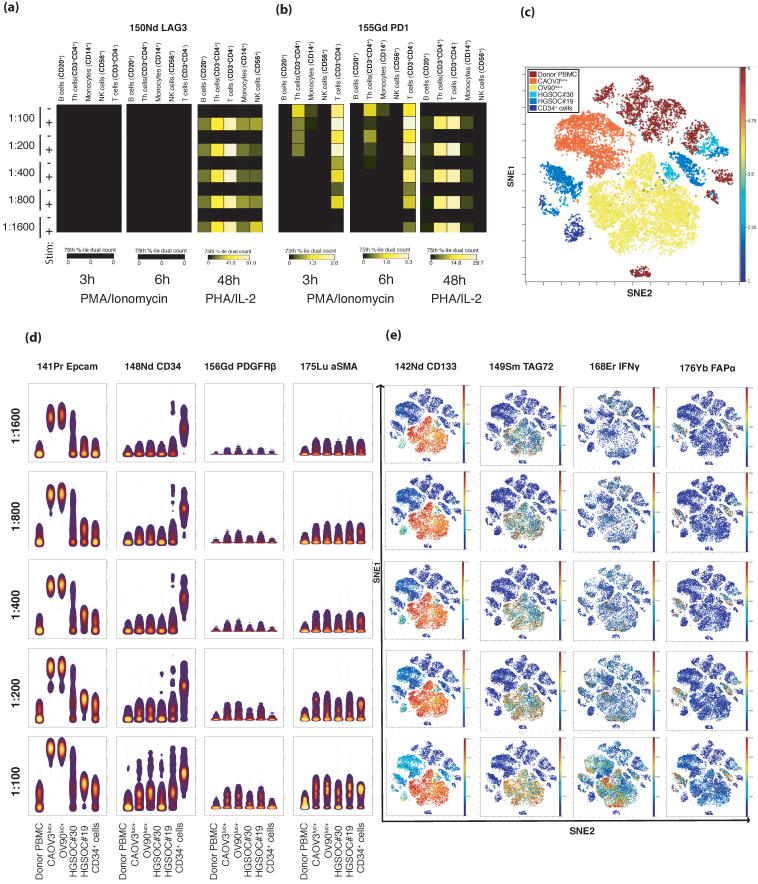
Titration of the panel antibodies using Cytobank software. (**a**,**b**) Immune checkpoint antibodies were titrated on both peripheral blood mononuclear cells (PBMCs) stimulated by phorbol 12-myristate 13-acetate (PMA) (25 ng/mL) + ionomycin (1 μg/mL) and PBMCs stimulated by phytohemagglutinin (PHA) (2.5 μg/mL) + interleukin 2 (IL-2). Heatmaps show that LAG-3 (**a**) was only expressed after stimulation by PHA/IL-2 and that PD-1 expression (**b**) differed with the dilution in the cells stimulated by PMA/ionomycin, while expression was consistent across all dilutions tested on PHA/IL-2-stimulated cells. (**c**) A viSNE plot was generated after pooling all samples from the stromal marker and tumor cell marker titration experiments, and the different samples were color coded. The results demonstrate a distinct separation of ovarian cancer cell lines (Caov-3 and OV-90) from the other samples, while the two dissociated tumor samples (HGSOC#19 and HGSOC#30) and the stem cells (CD34+ cells) show overlapping phenotypes, as well as some cellular similarity with the healthy donor sample (donor PBMCs). (**d**) Illustration of the gating strategy of the concatenated .fcs files to visualize immune staining. Plots display the sample-wise staining pattern in six samples (in the columns) of four selected markers, two tumor (EpCAM and CD34) and two stromal (PDGFRß and αSMA) antibodies (horizontally) in a dilution series from 1:100 to 1:1600 (vertically). (**e**) The viSNE plot in (**c**) color coded according to the specific antibody expression of four antibodies (horizontally) in the combined samples according to titration levels (vertically), from the most diluted on the top to the least diluted on the bottom.

The development of the CyTOF panel was the foundation for all further analyses. Based on a literature review, we designed a panel to define the major immune, stromal, and tumor subsets of the HGSOC TME [20,21] (Table S1). The panel was developed according to an optimization process, whereby all antibodies were titrated on a mixture of cells to include positive and negative marker controls (Figure 1, Table S1). A backbone panel containing immune markers (*n* = 15) identified either on the cell surface or intracellularly was titrated on unstimulated healthy peripheral blood mononuclear cells (PBMCs). Then, healthy PBMCs, both unstimulated and stimulated by the cytokine-producing agents phorbol 12-myristate 13-acetate (PMA), ionomycin, phytohemagglutinin (PHA), and interleukin 2 (IL-2), were used to determine optimized titer values of the cell surface antibodies that identify immune checkpoints (*n* = 6). The tumor (*n* = 11) and stromal (*n* = 4) markers were titrated on a mixture of two HGSOC cell lines, two dissociated primary tumor tissues, CD34+ cells, and unstimulated and stimulated healthy PBMCs (Figure 1, Table S1 and Figures S2–S6).

As a final step, all markers in the panel were titrated on a mix of four primary patient samples, as well as on two HGSOC cell lines, one CD34+ cell line, and PBMCs (Figure 1, Table S1, Supplementary Material 4). After initial gating steps, including Gaussian gating [22], the X-shift algorithm [23] was applied to the debarcoded CyTOF files. The cellular expression patterns of the panel markers were identified in the positive and not in the negative controls, which confirmed the specificity of marker expression. When the antibody panel was applied to dissociated primary tumor tissues, the resulting data confirmed that the panel could identify tumor cells and common infiltrating immune cells, such as T cells, B cells, resident tissue macrophages/monocytes, and natural killer cells. Cells belonging to the tumor stroma, including pericytes, endothelial cells, and fibroblasts, could also be distinguished successfully, and the data analyses could identify markers expressed either on cell subsets (such as stem cells) or activated cells or antibodies that are classified as immune checkpoint markers.

### 2.2. In-Depth Tissue and Cell Phenotyping of the Major TME Cell Populations

**Figure 2:**
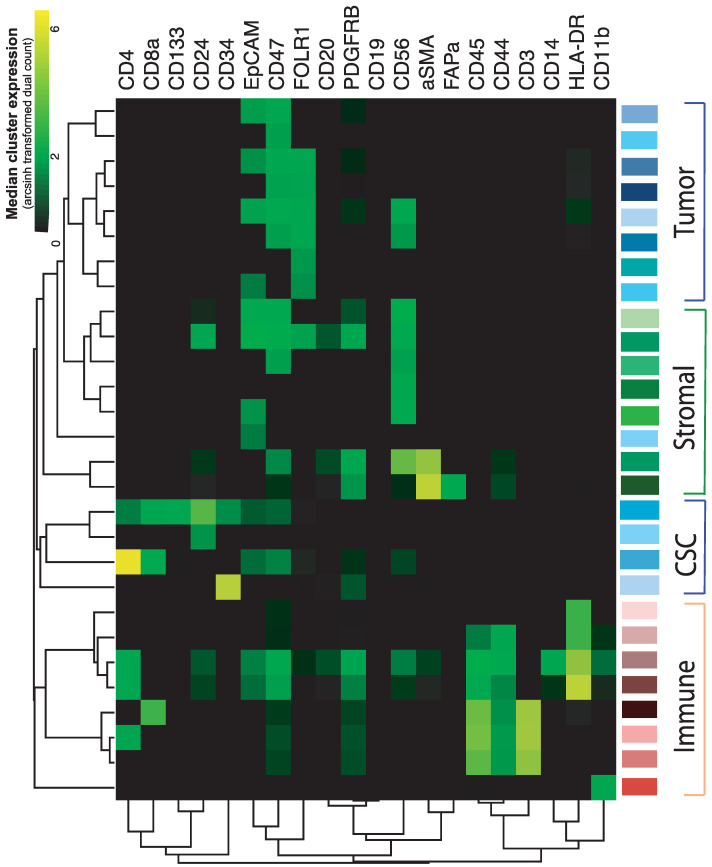
Heatmap showing the hierarchical clustering of data based on the identification of the main cellular subsets (tumor cells, stromal cells, immature or stem cells [CSC], and immune cells; y-axis) by the cellular expression of antibodies (x-axis). Data were generated from all dissociated samples from three patients and illustrate the 28 most common cell populations detected in the combined data set.

An analysis of the data from three patient samples dissociated by six different methods resulted in the identification of 28 populations for further analysis. When all data were combined, common infiltrating immune cell subsets were distinguishable, including three T cell subsets (CD3+ cells), antigen-presenting cells (HLA-DR+ cells), macrophages/monocytes (CD14+ cells), and granulocytes (CD11b+ cells) (Figure 2). Among the tumor cell clusters identified, two separately clustered categories of cells were observed: tumor cells expressing EpCAM, FOLR1, or both, with high expression of CD47 and those without high expression of CD47 (total clusters *n* = 10). The most abundant stromal populations of the three tumors included in the experiment were endothelial cells (PDGFRβ+), fibroblasts (CD56+ cells), and cancer-associated fibroblasts expressing αSMA and FAPα (Figure 2). A separate hierarchical cluster of immature or stem-like cell populations (*n* = 4) was identified, which expressed CD133, CD34, or both, as well as CD24, a marker found on both immature leukocytes and ovarian cancer cells [24,25].

Among the stem-like populations, one seems to be previously undetected in ovarian tumors (the uppermost CSC cluster in Figure 2), where the cells express the stem cell markers CD133, CD34, CD47 and the lymphocyte markers CD4 and CD8 but not CD3, in addition to the tumor markers CD24 and EpCAM.

### 2.3. Phenotypic Differences between the Patient Samples

**Figure 3:**
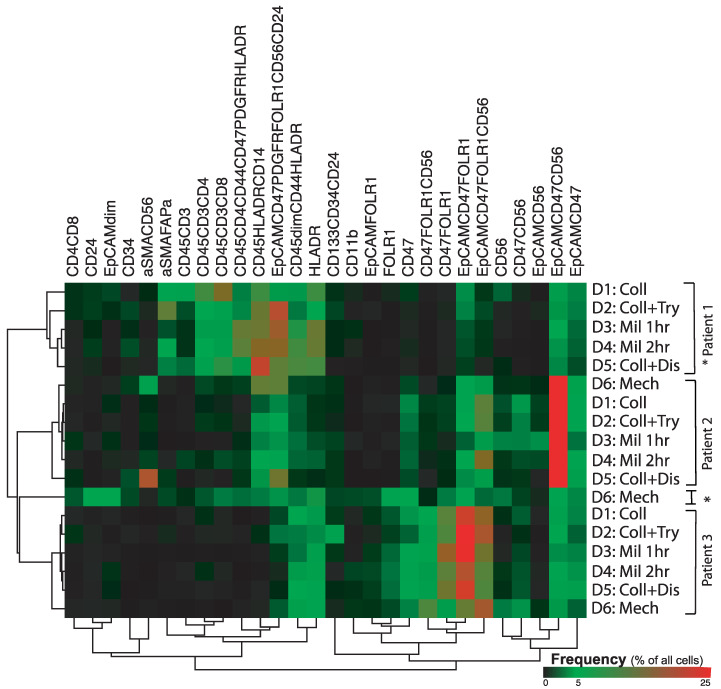
Hierarchically clustered heatmap showing variation in antibody expression (columns) in ovarian tissues from three patients using six different tissue dissociation methods (rows): collagenase, collagenase, and trypsin, Miltenyi enzyme mixture for 1 h, Miltenyi enzyme mixture for 2 h, collagenase and dispase, and mechanical dissociation. When the samples were clustered hierarchically according to antibody expression, the mechanically dissociated (D6) Patient 1 tumor, marked *, did separate from the rest of the samples and clustered between the samples from Patient 2 and Patient 3. The other samples cluster according to patient rather than according to dissociation method applied to the tissue.

Data from the tumor tissues of three patients, with each tumor dissociated by six separate methods, were combined, and the proportions of the main cell subsets were compared. This showed that the cellular composition varied greatly across tumor tissues (Figure 3). While patient 1′s tumor showed the presence of several CD45+ cell subsets regardless of the dissociation method, the tumors of patients 2 and 3 only showed cell expression of CD14+ leukocytes and HLA-DR positive cells, respectively. For the stromal cell subsets, all dissociated tissues from patient 1 showed expression of αSMA, while this marker was only found in tissues from patient 2 that were dissociated by the Miltenyi cocktail (for either 1 h or 2 h.) and was not found in any of the tissue samples from patient 3. Regarding the tumor cells, all dissociated tumors from all three patients contained cells expressing EpCAM and CD47, with and without positive CD56 expression. However, the EpCAM+ CD47+ CD56+ cell subset showed a method-independent higher number of these cells (event count) in the tumor of patient 2. The cells with FOLR1 expression were mainly found in the tumor tissues from patient 3, but the mechanically dissociated tissue of patient 1 also contained this cell subset. Regarding the fibroblast subsets defined as CD56+, those coexpressing CD47 were predominantly found in tumor samples from patients 2 and 3, while the cell subset defined singly by CD56 expression was mainly found in the mechanically dissociated tissues from all three patients.

### 2.4. Dissociation Method-Related Differences

In a hierarchical clustering of all samples, marker expression differed somewhat between disparately treated tissues from the same patient, in accordance with the results demonstrated in Figure 3. However, all but one mechanically dissociated sample (from patient 1) clustered according to the patient from whom the sample originated rather than according to the dissociating enzyme mixture used on the sample.

**Figure 4:**
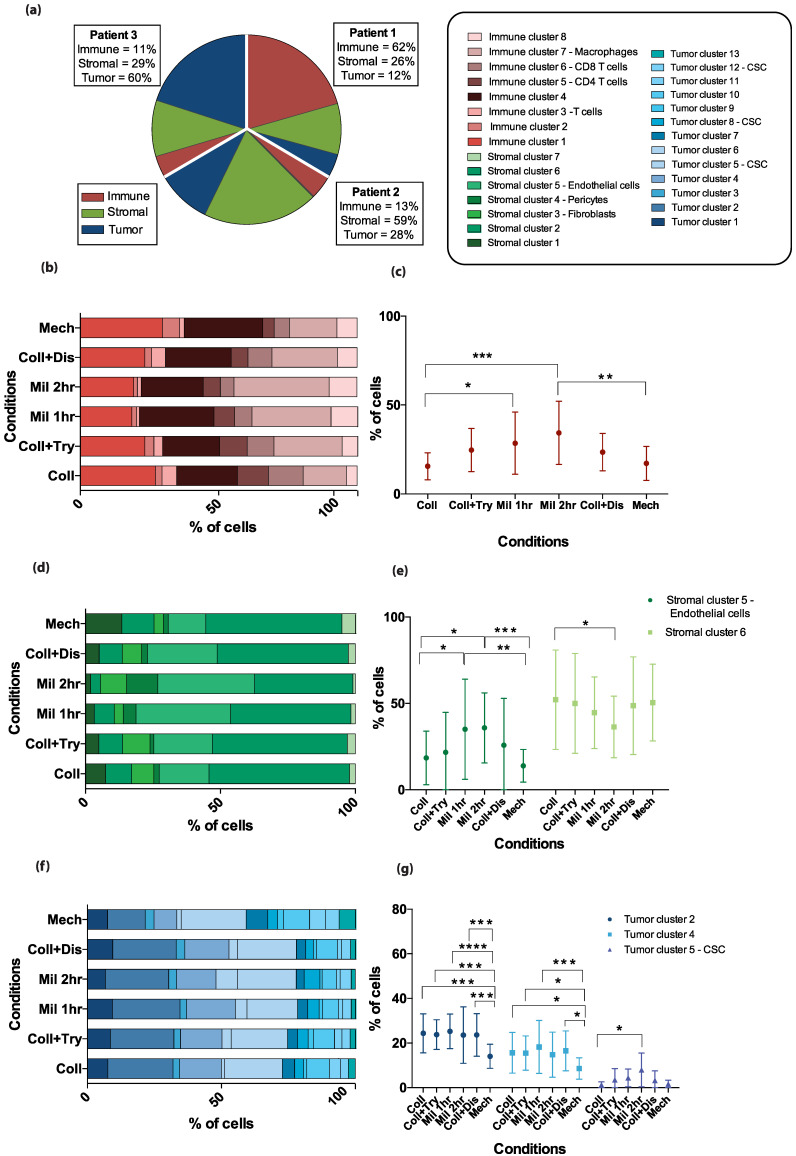
Differences in specific cell cluster proportions of the total cell population by dissociation method. (**a**) The pie chart displays the proportions of the three different metaclusters—immune cells in red, stromal cells in green, and tumor cells in blue—in three patients, out of a total of 300%. The graph is based on the pooled results from the six dissociation methods. The patient-specific fractions of each metacluster are shown in the text boxes. (**b**,**d**,**f**) Stacked bar plots illustrate how the identified clusters (color coded according to legend in (**a**)) within the metaclusters are proportioned in the combined patient samples according to the dissociation method applied for (**b**) immune cells, (**d**) stromal cells, and (**f**) tumor cells. (**c**,**e**,**g**) Error plots displaying the mean percentage of cells in the combined data from three patient samples with standard deviations. The specific clusters included in the figure are those where significant differences between dissociation methods were found by a two-way ANOVA analysis with Tukey’s multiple comparisons test. * *p*-value less than or equal to 0.05. ** *p*-value less than or equal to 0.01. *** *p*-value less than or equal to 0.001. **** *p*-value less than or equal to 0.0001.

When the three main cellular subsets—the immune, stromal, and tumor cells—were examined separately in the combined patient pool, a dissociation method effect was reflected in the resulting proportions of specific cell clusters that contributed to one main subset, as illustrated by the stacked heatmaps in Figure 4b,d,f. Mechanically dissociated tissues and single cells resulting from enzymatic dissociation by collagenase alone demonstrated lower fractions of immune cluster 7 and stromal cluster 5 compared to cell suspensions generated by the Miltenyi enzymatic cocktail (Figure 4c,e). For the stromal cluster 6 the opposite effect was seen as the percentage of cells in this cluster was higher when tissues were treated enzymatically by collagenase than by the Miltenyi cocktail for 2 h. Cells dissociated using the mechanical dissociation method differed from those dissociated by most enzymatic methods in terms of the expression of tumor cell clusters 2 and 4, while the expression of tumor cell cluster 5 differed significantly between tissues treated enzymatically by collagenase alone and those treated by the Miltenyi cocktail for two hours (Figure 4g). An overview over the markers defining the cell clusters which were affected discordantly by different dissociation methods (Figure 4c,e,g) can be found in Table S2.

An investigation of functional markers in each of the 28 identified main cell clusters according to dissociation method showed no significant differences in the median marker expression despite a trend toward lower expression in the mechanically dissociated tissues compared to samples dissociated by other methods (Figure S6). The functional markers demonstrated expression in cellular subsets similar to that commonly reported in the literature; T cells and some tumor clusters expressed markers for immune checkpoints, while tumor-associated fibroblasts (CD47+ CD56+ cells) and some tumor cell subsets (CD47+ FOLR1+ cells) did not demonstrate any increased expression of functional markers. Further analysis indicated mutually exclusive PD-1 expression on CD4-positive T cells and PD-L1 expression on the population of CD14+ monocytes/macrophages.

## 3. Discussion

In solid cancers, the TME can promote immunosuppressive mechanisms, tumor vascularization, growth, and metastasis [26,27,28]. The challenging heterogeneity and dynamic nature of solid tumors, as well as the inherent chemo-resistance present in many tumors, appear to be influenced considerably by the cellular composition of, and interactions within the TME [26,27,28]. Consequently, a significant number of studies have been conducted to identify predictive or prognostic markers in the TME of HGSOC using molecular methods [2,9,29,30,31,32,33]. Published research on the TME often focuses on one main cellular subset, usually tumor cells or immune cells [4,7,13,14,15,16]. However, the impacts of cancer stromal cells on clinical outcomes are also increasingly being investigated [2,34,35,36]. A more comprehensive mapping of the tissue composition of the TME could help reveal the phenotypic diversity of tumors [7]. The development of a mass cytometry panel involves a step-by-step optimization process [37,38]. In the recent past, several mass cytometry panels have been developed to investigate a range of dissociated cancers [19,37,39,40,41], but only a few of these publications have focused on the panel development process [19,37,42]. As all molecular or analytical techniques entail inherent biases, an awareness of how the techniques contribute to skewing results can prevent undetected weaknesses, facilitate more precise comparisons, and improve the reproducibility of experiments. Here, we describe the development of a CyTOF panel to identify the tumor, stromal, and immune cell subsets found in HGSOC tissues, as well as potential novel cell subgroups. We also examine how different commonly used methods for dissociating tumors into single cells in suspension can impact the results of CyTOF analyses.

Dissociated tissues can be examined by multiparameter flow cytometry with a relatively high cell throughput, but the overlapping emission spectra of the fluorescent dyes restrict the possibility for simultaneous examination of over 20 antibodies, although new methods are being developed to circumvent this restriction [43,44]. Histopathologic examination with immunohistochemistry analyses is another commonly applied method used both clinically and in research to identify the cellular composition of tumors and phenotype the cancer based on both morphology and antibody expression. While this method provides spatial information that will be lost in analyses of disseminated tissues, an advantage of the CyTOF method over both immunohistochemistry and flow cytometry is that the cellular expression of over 30 antibodies can be examined simultaneously [45].

Only a few studies using the suspension CyTOF technique on tumor tissues have been published. In our study, the fcs. file debarcoding process posed a major challenge, as the data in the generated files represented a mix of cells with vastly varying cell sizes and iridium content, which is not the case with data generated from experiments focusing on PBMCs. Gonzalez et al. addressed this issue by writing a debarcoding algorithm to apply to CyTOF data from HGSOC tumors [13]. For the current study, we manually debarcoded each of the files separately to achieve a balance between including as many live cells as possible from each sample and excluding debris. This process was performed using the debarcoding tool in the Fluidigm software and adapting both the barcode separation and Mahalanobis distance according to the barcode pattern seen in the files.

Gonzalez et al. developed a panel of 41 antibodies to interrogate the HGSOC tumor compartment [13], while Toker et al. used a panel focusing on the infiltrating T cell populations of the TME [14]. Each study interrogated specific compartments of the TME in detail. By contrast, our panel provides a broader overview of the main populations of the HGSOC TME. Like the other researchers, we were able to identify potential new cellular subsets within the specific populations when the panel was used on HGSOC tissues. Gonzalez et al. identified how tumor resistance to the chemotherapy drug carboplatin differed in two novel vimentin-positive subpopulations according to the expression of cMyc and human epididymis secretory protein 4 (HE4) [13]. Similarly, Casado et al. show how specific, previously undescribed, tumor subsets seem to be associated with the disease state. These subsets include cells with high expression of MUC1 and CD147, minimal Ki67 enrichment, and low ERK1-2 signaling, which were found to be present in greater numbers in recurrent tumors than in samples from a primary treatment setting [16]. In the present study, a previously undescribed stem-like cell subset is detected, expressing the following markers: CD24, EpCAM, CD133, CD34, CD47, CD4, and CD8. As the study cohort consists of only three patients, further validation of the finding in a larger patient material is warranted. Furthermore, an exploration of how presence or distribution of this cell subset associates with specific phenotypic outcomes, such as tumor resection rates or progression-free intervals could add translationally applicable information.

Application of our panel enables the TME profiles of the individual patient to be distinguished, and it is hoped that further use of the method on different patient cohorts could contribute to identifying how differences in TME composition impact clinical outcomes. The results of this study illustrate a sufficient depth of phenotyping when the developed CyTOF panel is applied to patient samples. For a more in-depth characterization of particular cell populations, possible solutions [13,14] include using dedicated panels with a focus on specific cell subsets, increasing the sample sizes or combining different panels using panel merging algorithms [6]. Combining the results from tumor analyses with clinical data from prospectively collected biobanks and implementing the method in clinical trials could increase its clinical translatability.

Our panel was optimized for single-cell suspensions obtained from primary tumor samples, and the six dissociation methods were selected to cover the spectrum of regular dissociation protocols applied to ovarian tumors [13,46]. In our study, we found that the method of tissue dissociation affected the marker expression of a few subgroups within the three major cell subsets. Similar effects on immune cells have been identified by Poláková et al. [19], who compared dissociation of tonsillar carcinoma tissues by two collagenases combined with Dnase 1. They found that the dissociation method significantly affected the expression of major lymphocyte markers [19]. Leelatian et al. examined the cellular effects of tumor dissociation methods on tissues from gliomas, tonsils, and melanomas and found that disaggregation conditions affected the various cell populations differently [18]. The observations from these studies and our own indicate that the lack of reproducibility of results from tissue-focused CyTOF studies may be partly due to the application of different tissue dissociation methods. Furthermore, our findings demonstrate the need for careful selection of dissociation method to suit the study hypothesis when planning experiments, as some markers of interest might be dimly or not expressed if particular dissociations are used, which may lead to a failure to detect important cell subsets. In particular, the cell subset-related results from studies applying mechanical dissociation of tissues needs to be interpreted with caution.

There are several limitations to this study, including a lack of validation of the findings by other methods. By adding more antibody-positive controls to the experiments, particularly for the stromal markers, and allocating part of the tumor sample for immunohistochemistry examination of the fresh frozen tissue it would be feasible to validate the presence of the cell subsets identified in the dissociated tumor samples. The sample size is small, which might cause dissociation method-related differences to go undetected. Still, with these three samples it was possible to detect changes in antigen expression in different cellular subsets that are important to recognize when future studies applying dissociated HGSOC tissues are planned.

In our experiment, we examined three patient samples dissociated by six different methods to ensure that we would be able to detect the different main cell subsets and investigate the potential impacts of the dissociation methods. The results of this study demonstrate greater interpatient differences than intrapatient differences and confirm the acknowledged tumor heterogeneity, as each patient sample displayed a unique combination of tumor, immune, and stromal cells, regardless of the method used for tumor dissociation. Several authors have illustrated the challenges of developing reliable diagnostic and therapy-related methods that can be applied to heterogeneous cancer tissues [13,47,48,49]. The polyclonality and heterogeneity of HGSOC tumors, which has been established through single-cell experiments [13,47,48,49], indicate a need for better profiling tools. Application of CyTOF in studies of HGSOC to detect cellular patterns rather than single biomarkers, potentially in combination with genetic biomarkers and preclinical modeling systems may provide breakthroughs. The CyTOF methodology has been extended during the last years, with the introduction of a method that combines CyTOF with imaging to examine single cells in tissue sections [50]. Thus, the adaptation of CyTOF panels for examination of solid tissues by this combined method is ongoing. It is our hope that as an increasing number of studies using imaging mass cytometry are published, concerns regarding the effects of the tumor dissociation methods on data generation will be eliminated. Studies on the cellular components of tumors and their impact on clinical outcomes often cannot be reproduced and differences in methodology, and such tumor dissociation methods could be a factor contributing to discrepancies in results. Hopefully, in future studies the variability in antibody expression induced by the dissociation method will be considered during the planning phase of new experiments.

## 4. Materials and Methods

### 4.1. Samples

#### 4.1.1. Patient Sample Collection

We used samples from four patients with primary advanced HGSOC (Stages IIc–IIIc) admitted to the Department of Obstetrics and Gynecology, Haukeland University Hospital (HUS), Bergen, Norway. All samples were included in the Bergen Gynecologic Cancer Biobank (GYNCAN). The tumor samples resected during primary debulking procedures were obtained from the primary ovarian tumors and placed directly in Dulbecco’s Modified Eagle’s Medium (DMEM) and transferred to the laboratory for immediate processing. However, our experiments on dissociation method effects required sufficient tissue for the application of six separate dissociation methods, which we could only obtain from three of the four patients. Clinical and histopathological data on these three patients can be found in the Table S3. Informed consent was obtained from the women before collection of the tumor samples was initiated.

#### 4.1.2. Ovarian Cancer Cell Lines

The human ovarian serous adenocarcinoma cell lines OV-90 (American Type Culture Collection [ATCC]**^®^**CRL-11732 ™) and Caov-3 (ATCC**^®^**HTB-75 ™) were obtained from ATCC, VA. The OV-90 cells were cultivated in RPMI 1640 medium, and the Caov-3 cells were cultivated in DMEM medium. Both media were supplemented with 10% heat-inactivated fetal calf serum (FCS), 2 mM L-glutamine, and penicillin 100 IU/mL (all from Gibco, Thermo Fisher Scientific, Waltham, MA, USA). Cells were grown in 75 cm^2^ cell culture flasks (Costar, MA, USA) at 37 °C in a humidified atmosphere with 5% CO_2_ and subcultured twice a week. Single-cell suspensions were obtained by washing the cells twice with phosphate buffered saline (PBS) (1:10 dilution of 10× stock PBS; Dulbecco’s tablets, Oxoid Limited, Thermo Fisher Scientific, Waltham, MA, USA) before incubation with trypsin (Gibco, Thermo Fisher Scientific). As a final step, cells were washed one last time, freezing media (90% FCS and 10% dimethyl sulfoxide (DMSO) (Sigma Aldrich, St. Louis, MO, USA)) was added, and the cells were cryopreserved.

#### 4.1.3. Stem Cells

The CD34+ stem cells had been collected as part of the Research Biobank for Blood Diseases (REC ID 2015/1759), HUS, Bergen, Norway, by a standardized process described elsewhere [51].

#### 4.1.4. Healthy Donor Peripheral Blood Mononuclear Cells (PBMCs)

Peripheral blood from healthy donors was collected at the Blood bank, Department of Immunology and Transfusion Medicine, HUS, Bergen, Norway. The sampling was approved by the REC (REC ID 2012/2247). The PBMCs were isolated by density gradient centrifugation with Lymphoprep (Axis-Shield, Oslo, Norway), cryopreserved in 90% FCS and 10% DMSO and frozen in a Mr. Frosty container (Thermo Fisher Scientific) at −80 °C for 24 h before being stored at −150 °C.

### 4.2. Stimulation of PBMCs

To enable identification of the optimal dilution of antibody markers for use in further experiments, including markers expressed in activated cells only, all markers for immune cells and immune checkpoints were titrated concomitantly on unstimulated and stimulated PBMCs. Prior to stimulation, a batch of cryopreserved PBMCs were thawed, slow diluted (1:12) in RPMI 1640 medium at room temperature, and pelleted at 300 g for 5 min. The cells (3 × 10^6^ cells/mL) were then resuspended in complete RPMI 1640 medium and incubated for an hour at 37 °C in a humidified atmosphere with 5% CO_2_, prior to stimulation with either phorbol 12-myristate 13-acetate (PMA) (25 ng/mL) and ionomycin (1 μg/mL) for 3 or 6 h, or 2.5 μg/mL phytohemagglutinin (PHA, Sigma Aldrich) and 100 IU/mL Human Interleukin-2 (IL-2) Recombinant Protein (Gibco, Thermo Fischer Scientific) for 48 h under the same incubation conditions. After stimulation, the cells were fixed using Stable-Lyse and Stable-Store (Smart Tube Inc., San Carlos, CA, USA) as per the manufacturer’s protocol (Protocol number: SLSSP1TF-150203).

### 4.3. Tumor Dissociation Methods

Each tumor piece was divided into cubes (1 mm^3^) using a sterile scalpel, and the cubes were randomly assigned to dissociation methods. Tissue pieces were washed with either Hanks’ Balanced Salt Solution (Thermo Fischer Scientific) or PBS and transferred to 50 mL Falcon tubes filled with the respective pre-warmed (37 °C) enzyme mixtures. Each patient tumor sample was treated by six different dissociation methods (Table 1): five enzymatic and one mechanical (Mech). The enzymatic methods were collagenase II + calcium chloride (CaCl_2_) (Coll), collagenase + CaCl_2_ + TrypLE (Coll+Try), Miltenyi for one hour (Mil 1 h), Miltenyi for two hours (Mil 2 h), and collagenase + CaCl_2_ + dispase (Coll+Dis). The enzymatic dissociations were performed (1) by collagenase type II (1 mg/mL, Gibco, Thermofischer) in combination with CaCl_2_ (3 mM, Sigma Aldrich) for either two hours without (Coll), or with (Coll+Try) TrypLE Express Enzyme (1×) added afterwards for 5–10 min with no phenol red (Thermo Fisher Scientific); (2) by collagenase for 1 h with dispase (50 U/mL, Thermo Fisher Scientific) (Coll+Dis); or (3) by application of the Tumor Dissociation Kit from Miltenyi Biotech (as per manufacturer’s recommendations) for either 1 h (Mil 1 h) or 2 h (Mil 2 h).

The tissue samples in the different enzyme mixtures were transferred to a 37 °C incubator with 5% CO_2_ with continuous shaking at 250 rpm for the respective durations required for each dissociation method. Conventional mechanical dissociation (Mech, Table 1) entailed fine mincing of the tissue with sterile glass slides. After tissue dissociation, all cells, regardless of the dissociation method used, were strained through a 40 μm cell strainer, checked for cell viability with trypan blue staining, centrifuged (300 g, 5 min, room temperature), and cryopreserved in freezing media (90% FBS [Gibco] with 10% DMSO [Sigma Aldrich]) in a Mr. Frosty container at −80 °C for 24 h before being stored at −150 °C. The viability of cells in the dissociated tissues varied between patient samples and dissociation methods, with the lowest fractions of viable cells after mechanical dissociation (Table S4).

### 4.4. The CyTOF Panel

A 35-marker panel was developed with a focus on three components of the HGSOC tumor microenvironment: tumor cells, immune cells, and stromal cells (Figure S1, Supplementary Material 1, Table S1). The markers included in the panel were selected after a review of the literature. The associated metal conjugates were chosen based on information found in the web-based application Maxpar Panel Designer (Fluidigm, Markham, ON, Canada). Pre-conjugated antibodies (*n* = 24) were purchased from Fluidigm. For antibodies in the panel that were unavailable in the pre-conjugated form, in-house conjugation of carrier-free antibodies (*n* = 11) to metal-chelated polymers (Maxpar X8 Antibody Labeling Kits, Fluidigm) was performed according to the manufacturer’s protocol (PRD002 Version 11). The in-house-conjugated metal-labeled antibodies (Table S1, Supplementary Material 2) were diluted to 0.5 mg/mL in antibody stabilization solution (CANDOR Bioscience GmbH, Wangen, Germany) and stored at 4 °C.

### 4.5. Antibody Titration

All markers were initially validated and titrated on the ovarian cell lines OV-90 and Caov-3 and on the CD34+ cells and unstimulated and stimulated PBMCs (Figure S2, Figure S3, Supplementary Material 3). Then, the selected dilution of each marker in the panel was validated on cell suspensions from four human tumor samples (Figure S4, Supplementary Material 4). The provider, clone, and metal tag of each antibody and the controls used for titrations are listed in the (Supporting Information Table S1). Next, the selected dilutions of antibodies were further titrated on two primary tumor tissues dissociated by collagenase [D1] and Miltenyi 1 h [D3], respectively, as well as on the biobanked stem-cell-derived CD34+ cells and unstimulated and stimulated PBMCs. As a final step, all markers in the panel were titrated on primary patient samples (*n* = 4), each dissociated by one of the five methods: Coll [D1], Coll+Try [D2], Mil 1hr [D3], Coll+Dis [D5], or Mech [D6].

To investigate the effects of the dissociation methods, 18 barcoded samples (three patients with six dissociated samples per patient) and two barcoded controls (OV-90 cells and unstimulated PBMCs) were resuspended in cell staining buffer (CSB), pooled, pelleted, and washed twice with CSB at 800 *g* for 4 min at 4 °C.

### 4.6. Sample Preparation for Mass Cytometry Analysis

All analyzed samples were treated in the following way: Cryopreserved samples from the single-cell suspensions from the different dissociations and the control cells (OV-90, Caov-3, and from unstimulated and stimulated PBMCs) were rapidly thawed in a water bath, diluted (1:10) in their respective culture media, and centrifuged at 300 g for 5 min at room temperature. To assess cell viability, 5 μM of Cell-ID^TM^ Cisplatin (Fluidigm) was added to each sample for 60 s, before the effect of the cisplatin was quenched by Maxpar CSB (Fluidigm) and the cells were fixed according to the Stable-Lyse and Stable-Store protocol (No: SLSSP1TF-150203). The samples were counted and 1–3 × 10^6^ cells from each sample were barcoded using the 20-plex metal barcoding kit (Fluidigm) as per the Fluidigm user guide (version PN PRD023 B1). All the following steps were conducted at room temperature. For extracellular staining, samples were incubated with FcR blocking reagent (Miltenyi Biotec) at 1:10 dilution for 20 min, followed by antibody staining with 50 uL of a mixture containing CSB and 33 antibodies per 50 μL of cell sample (1:1 ratio) for 20 min. Cells were then washed twice at 800 *g* with CSB before 1 mL of methanol was added to each sample. The samples were incubated for 15 min, after which cells were washed twice with CSB and the pellets resuspended with CSB to a total of 50 μL. Then, 50 μL of the intracellular antibody cocktail containing antibodies against FoxP3 and interferon gamma was added to each sample to a ratio of 1:1, and the samples were incubated for 30 min. Cells were then washed twice at 500 *g* with CSB prior to resuspension for 1 h in 1 mL (125 nM/mL) of Cell-ID Intercalator-Ir (Fluidigm). After washing (800 *g*, 4 min) once with CSB and once with PBS, samples were diluted to 1 × 10^6^ cells/mL in Maxpar Cell Acquisition Solution containing 10% of EQ™ Four Element Calibration Beads (all reagents from Fluidigm) and run sequentially on a Helios mass cytometer (Fluidigm) through a wide bore injector system (Fluidigm).

### 4.7. Data Analysis

The .fcs files resulting from the mass cytometry experiments were normalized using internal bead standards, and the normalization algorithm in the CyTOF software v.6.7 (Fluidigm) was conducted before further analysis.

#### 4.7.1. Initial Gating and Debarcoding

Using analysis tools from the Cytobank platform (v7.2.0 and v7.3.0, Beckman Coulter, Inc., Indianapolis, IN, USA), initial gating of live singlets was performed using manual gating according to the four Gaussian parameters: center, width, offset, and residual [22]. Files were debarcoded with a 20-plex-debarcoding key (Fluidigm) using the Premessa debarcoder GUI package (https://github.com/ParkerICI/premessa, accessed on 4 December 2018) in R (version R 3.4.1 GUI 1.70 El Capitan build (7375)).

#### 4.7.2. Visualization Methods

T-Distributed Stochastic Neighbor Embedding (tSNE) is a nonlinear dimensionality reduction algorithm that reduces high-dimensional data down to two dimensions for easy visualization and rapid exploratory data analysis of any data type [13,52]. To visualize the expression pattern of the panel markers in two dimensions, tSNE maps were plotted in Cytobank (Figure 1b,d). The main cell populations were defined by manual gating according to marker expression and overlaid on the corresponding tSNE plot (Figure 1b). 

To further compare the effects of the six dissociation methods on the individual panel markers (and interpatient differences in the individual markers), live singlet cells were gated out according to the Gaussian parameters after CATALYST debarcoding of .fcs files. All live single cells from all files generated from the dissociated patient samples (*n* = 18) were then grouped separately according to the dissociation method and patient tissue used, and histograms displaying x/5 arcsin-transformed median expression of marker distributions were plotted in MATLAB v.R2019a (The MathWorks Inc., Natick, MA, USA) (Figure S5).

#### 4.7.3. Clustering

Debarcoded files containing the live single cells were concatenated for the purpose of unsupervised clustering according to cellular phenotypical resemblances by a density-based clustering method called X-shift (VorteX, Java version 1.0) [23]. X-shift finds the local density maxima of each data point (cell event), which becomes the cell cluster centroid in a nearest neighbor graph, using the weighted K-nearest-neighbor density estimation in a multidimensional marker space. The K value was automatically selected as 28 from the elbow point in a number of cell clusters versus K plot, with the software merging clusters by a Mahalanobis distance of less than 2.0.

#### 4.7.4. Bar Plots and Pie Charts

To visualize the differences in the percentage of the cell clusters between the tumor dissociation conditions for the different patients, Prism (6.0c version; GraphPad) was used to plot stacked bar plots in an XY table format using the clustering data obtained from X-shift (Figure 4). Prism software was also used to generate pie charts and compare cell subset fractions according to dissociation method (Figure 4).

#### 4.7.5. Heatmaps and Cox Proportional-Hazards Models

Heatmaps were generated in X-shift to efficiently visualize patterns and relationships in the high-dimensional mass cytometry data from the differently dissociated tumors. Using proportional numbers of live single cells from all the quality-controlled files, X-shift identified 91 cellular subsets. To enable identification of dissociation method effects on the main cellular subsets and their marker expression, all clusters constituting an average of <0.5% of the total cell numbers of each sample were excluded from further analyses. Based on the resulting 34 clusters, a hierarchically clustered heatmap was generated, and the cellular subsets that deviated only according to HLA-DR expression (*n* = 6 pairs) were manually merged. This resulted in 28 clusters on which the analyses were performed.

Arcsin-transformed dual counts of the median expression of functional markers in the panel were investigated using Cox proportional-hazards models for the cellular populations according to dissociation method (Figure S6).

## 5. Conclusions

The TME must be better understood if survival rates are to be improved for HGSOC. New tools for phenotypic characterization are necessary, especially when information on all the different components of the TME is needed at a single-cell resolution. This study describes how a 35-marker CyTOF panel for single-cell suspensions of HGSOC tissues can be used to define the three main parts of the TME. The study also provides an overview of the effects of dissociation methods on the cell subsets. The results show that the panel will be useful for investigating HGSOC tumors. Further, this study demonstrates how the reproducibility of study outcomes can improve if researchers take appropriate measures when generating single-cell suspensions. A combinatorial analysis method, such as that presented here, could replace single phenotype biomarker approaches and make it possible to identify treatment options in a more refined and personalized way.

## Figures and Tables

**Table 1:** The six tumor dissociation methods evaluated using the CyTOF panel.

Dissociations/ Conditions	Primary Enzyme	Duration	Additional Enzyme	Duration
Coll	Collagenase II + CaCl_2_	2 h		
2. Coll+Try	Collagenase II + CaCl_2_	2 h	TrypLE	5 min
3. Mil 1 h	Miltenyi	1 h		
4. Mil 2 h	Miltenyi	2 h		
5. Coll+Dis	Collagenase II + CaCl_2_	1 h	Dispase	30 min
6. Mech	Mechanical. − No enzyme added.	-	-	-

## Data Availability

The data presented in this study are available on request from the corresponding author. The data are not publicly available due to due to ethical restrictions.

## Supplementary Materials

The following are available online at https://www.mdpi.com/2072-6694/13/4/755/s1, Figure S1: Overview of ovarian mass cytometry panel development, Figure S2: Antibody staining on unstimulated and PHA-stimulated PBMCs, Figure S3: Stromal and tumor panels were titrated on ovarian cancer cell lines and a mixture of the patient samples, Figure S4: Panel validation on pooled patient data, Figure S5: Histograms illustrating antibody expression according to three separate patient samples, Figure S6: Cox proportional-hazard models of median expression of the functional markers. Table S1: Overview of antibodies and metal conjugates (Tags) included in the HGSOC TME-based mass cytometry by time-of-flight (CyTOF) panel, the positive and negative controls used for each antibody, and the final antibody dilutions that should be applied to tissues, Table S2: Antibody-expression of the cell clusters significantly affected by the different dissociation methods, Table S3: Overview of the patient cohort (*n* = 3), Table S4: Viability of the cells in the dissociated tissues. For each patient sample and the six dissociation methods the percentage of dead cells in the sample is listed. The viability was measured directly after dissociation of the tumor before freezing.
