# Supplementary material for: Phenotypic Characterization by Mass Cytometry of the Microenvironment in Ovarian Cancer and Impact of Tumor Dissociation Methods
Source: Cancers (Basel). 2021 Feb 11;13(4):755. doi: 10.3390/cancers13040755 (PMC7918057; doi:10.3390/cancers13040755)

Figure S3.1: Histogram - aSMA

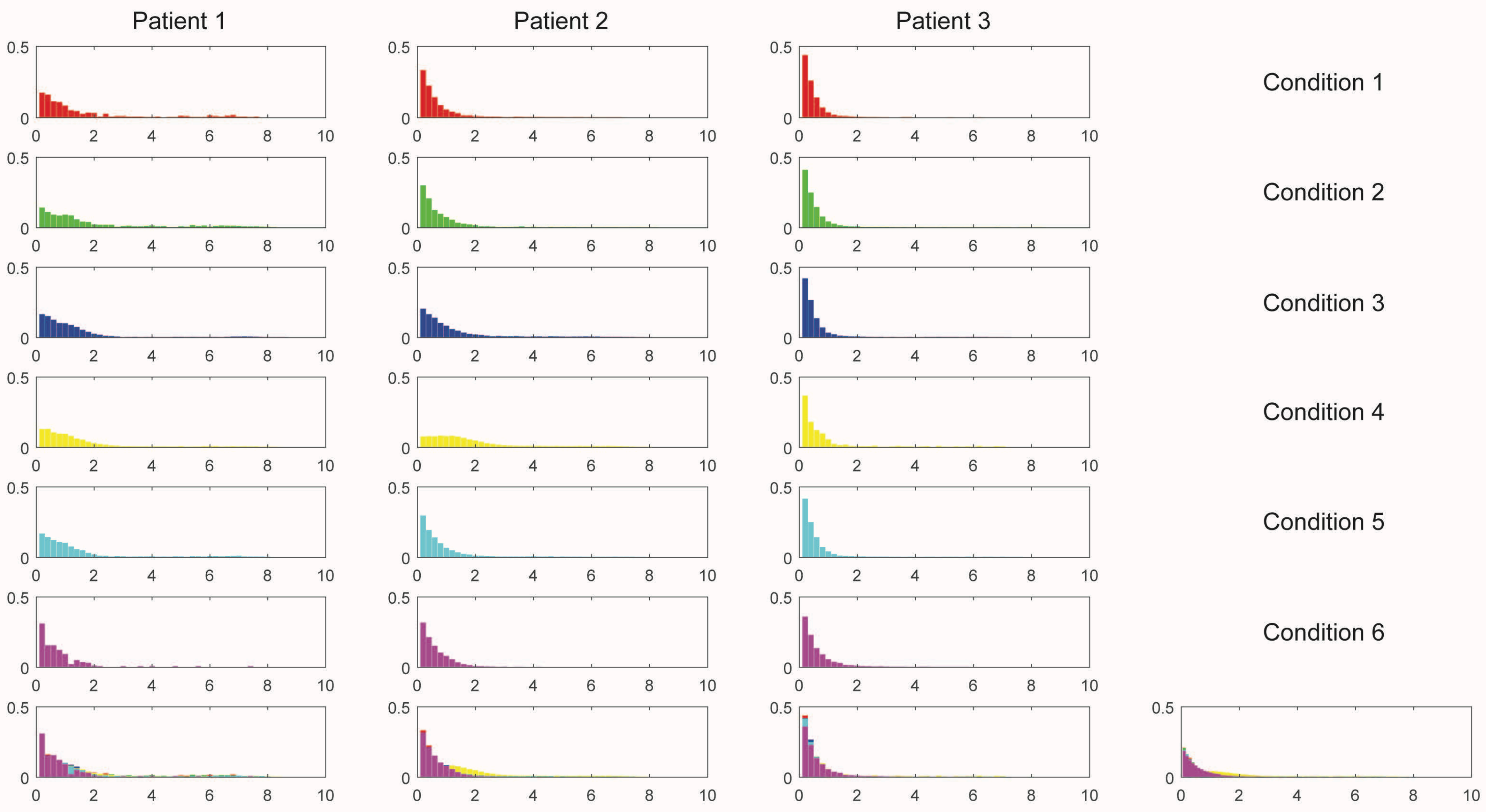

Figure S3.2: Histogram - Axl

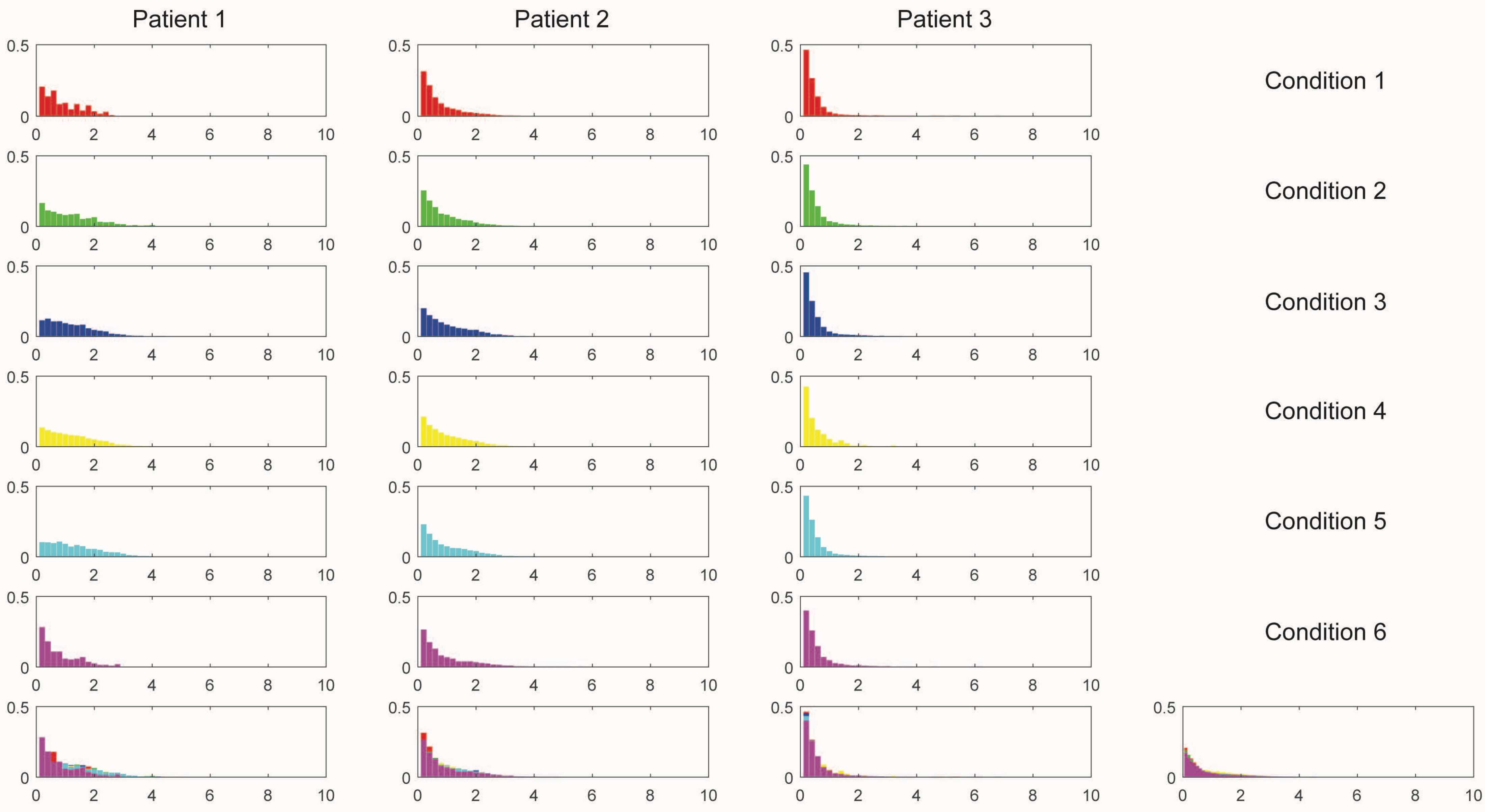

Figure S3.3: Histogram - CD3

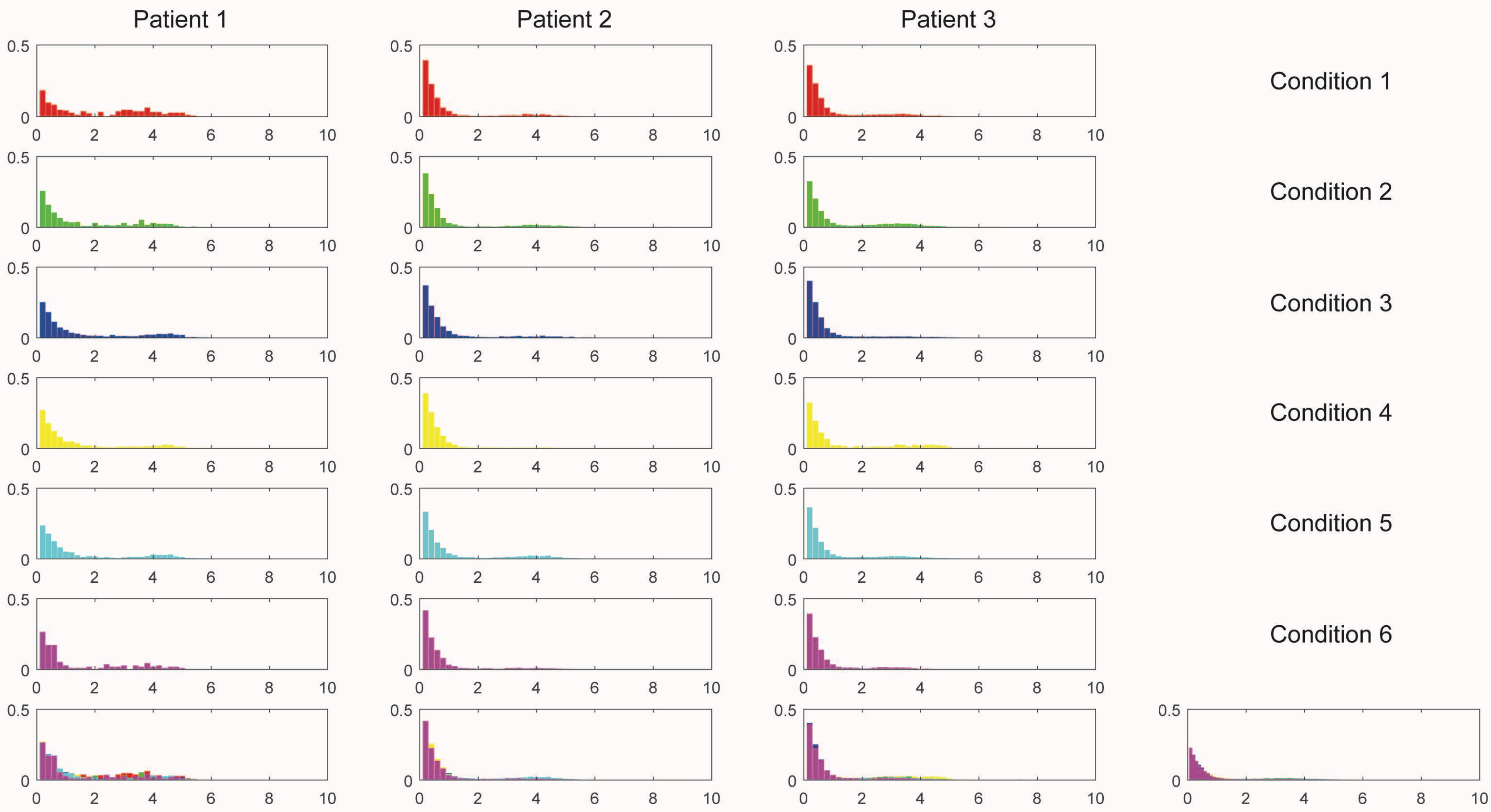

Figure S3.4: Histogram - CD4

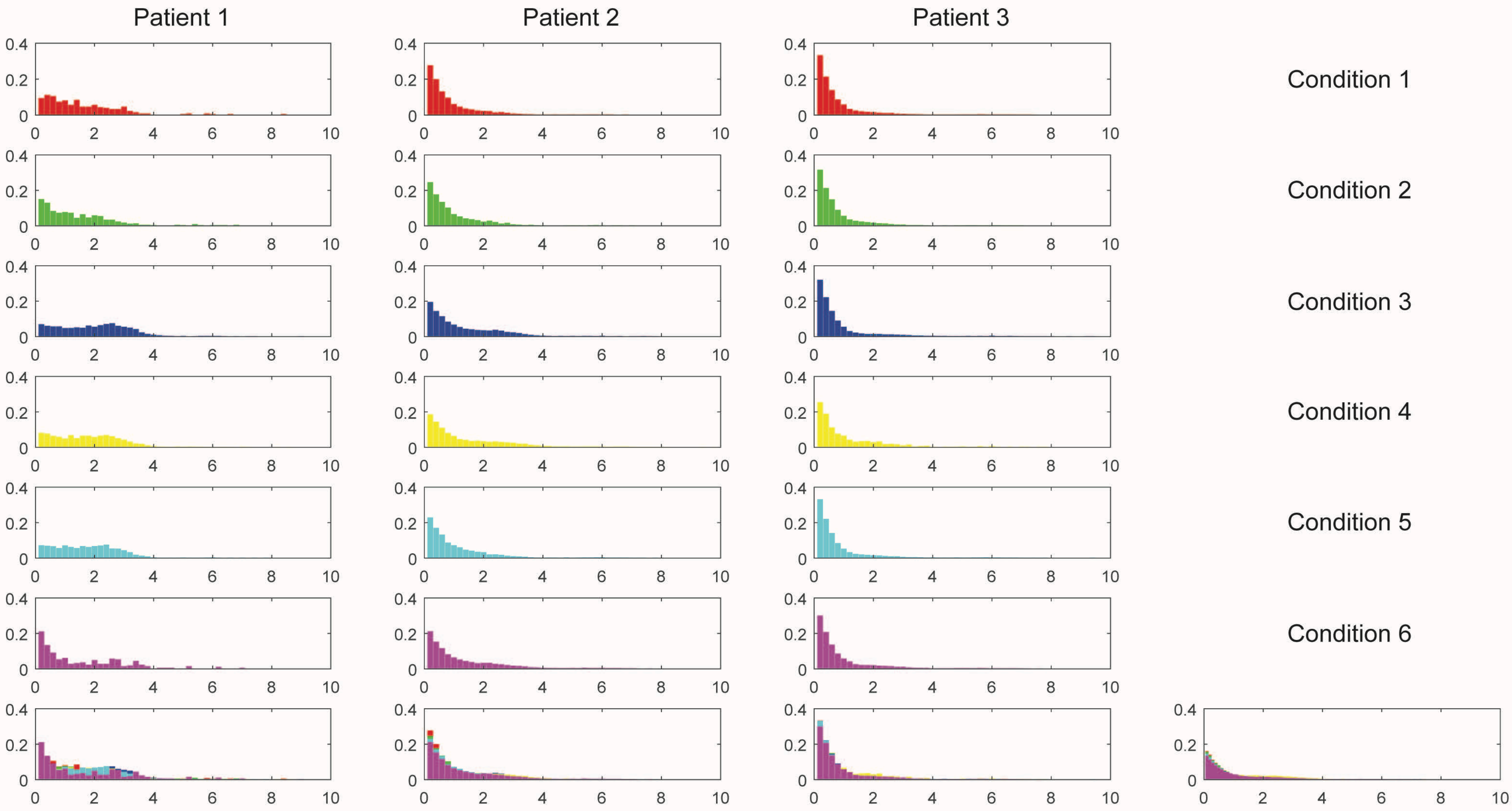

Figure S3.5: Histogram - CD8a

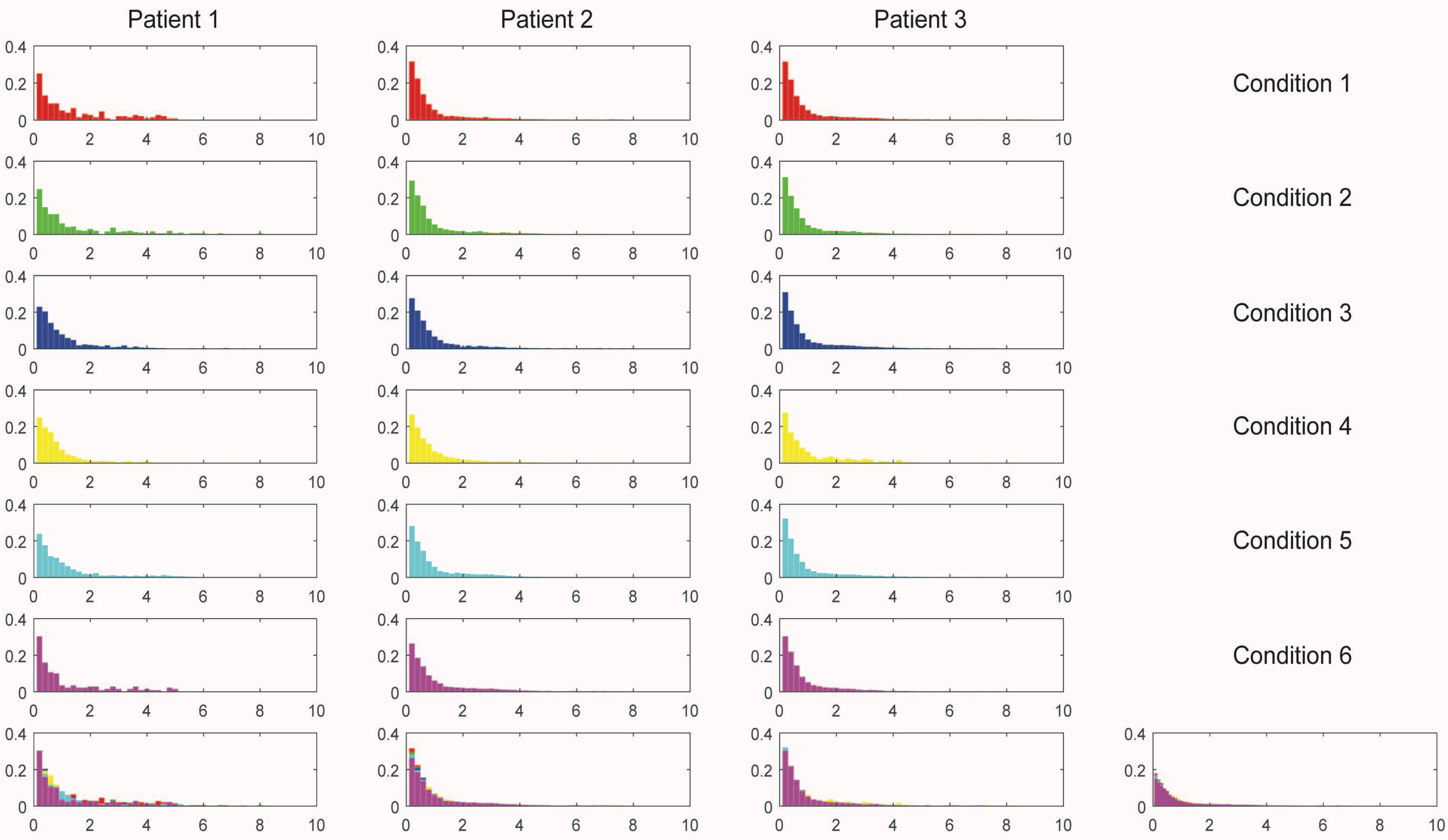

Figure S3.6: Histogram - CD11b

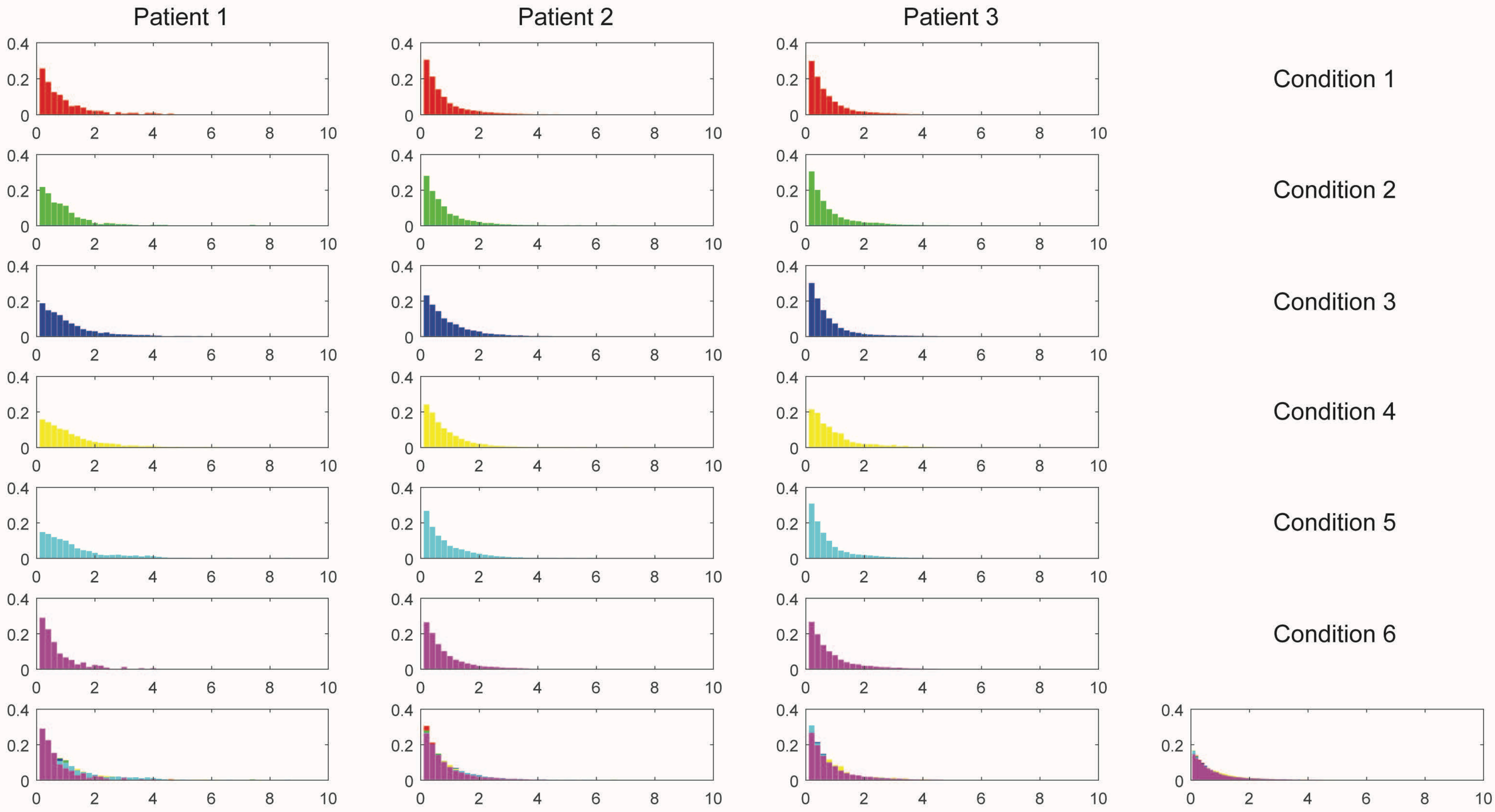

Figure S3.7: Histogram - CD14

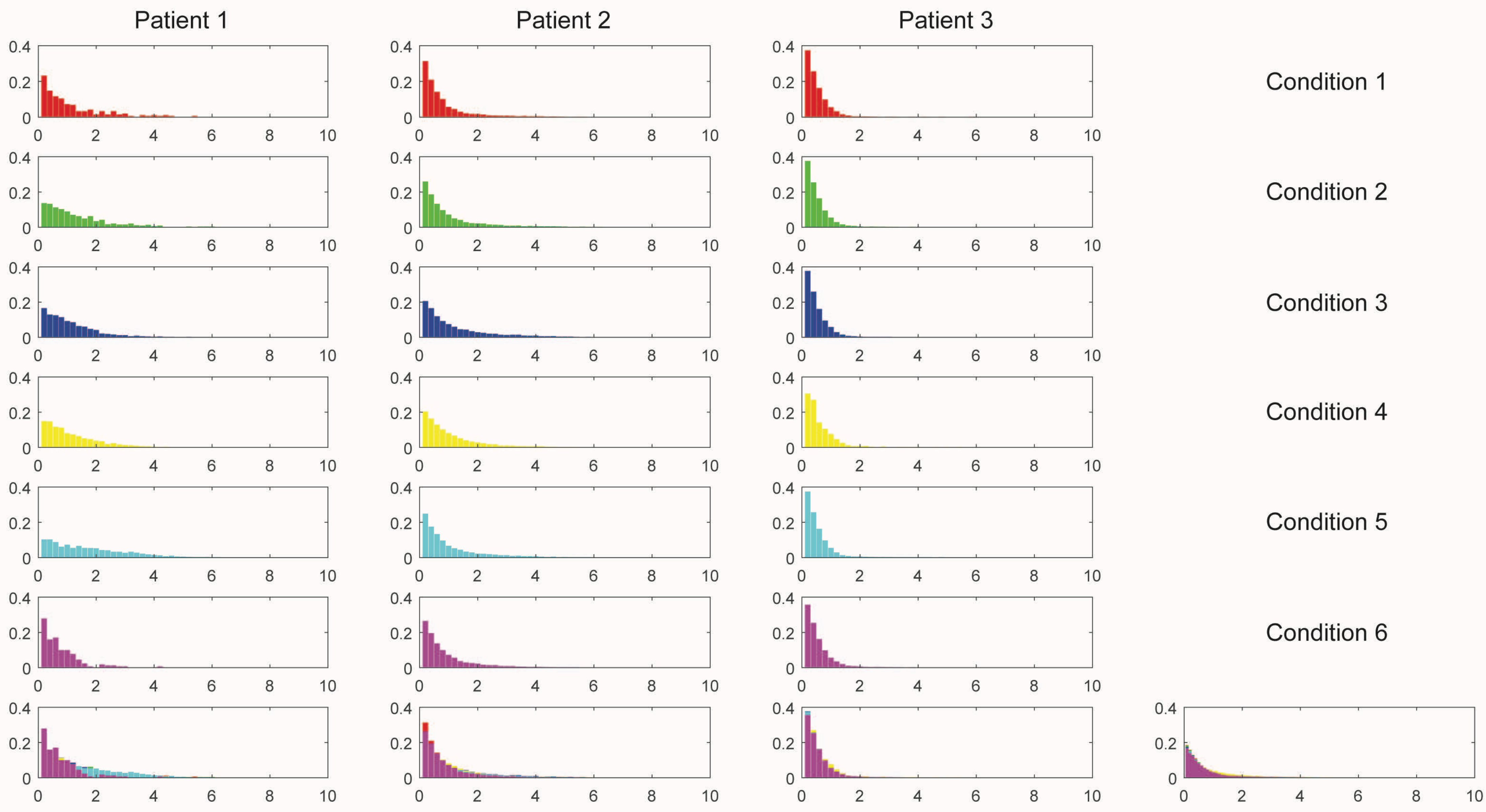

Figure S3.8: Histogram - CD19

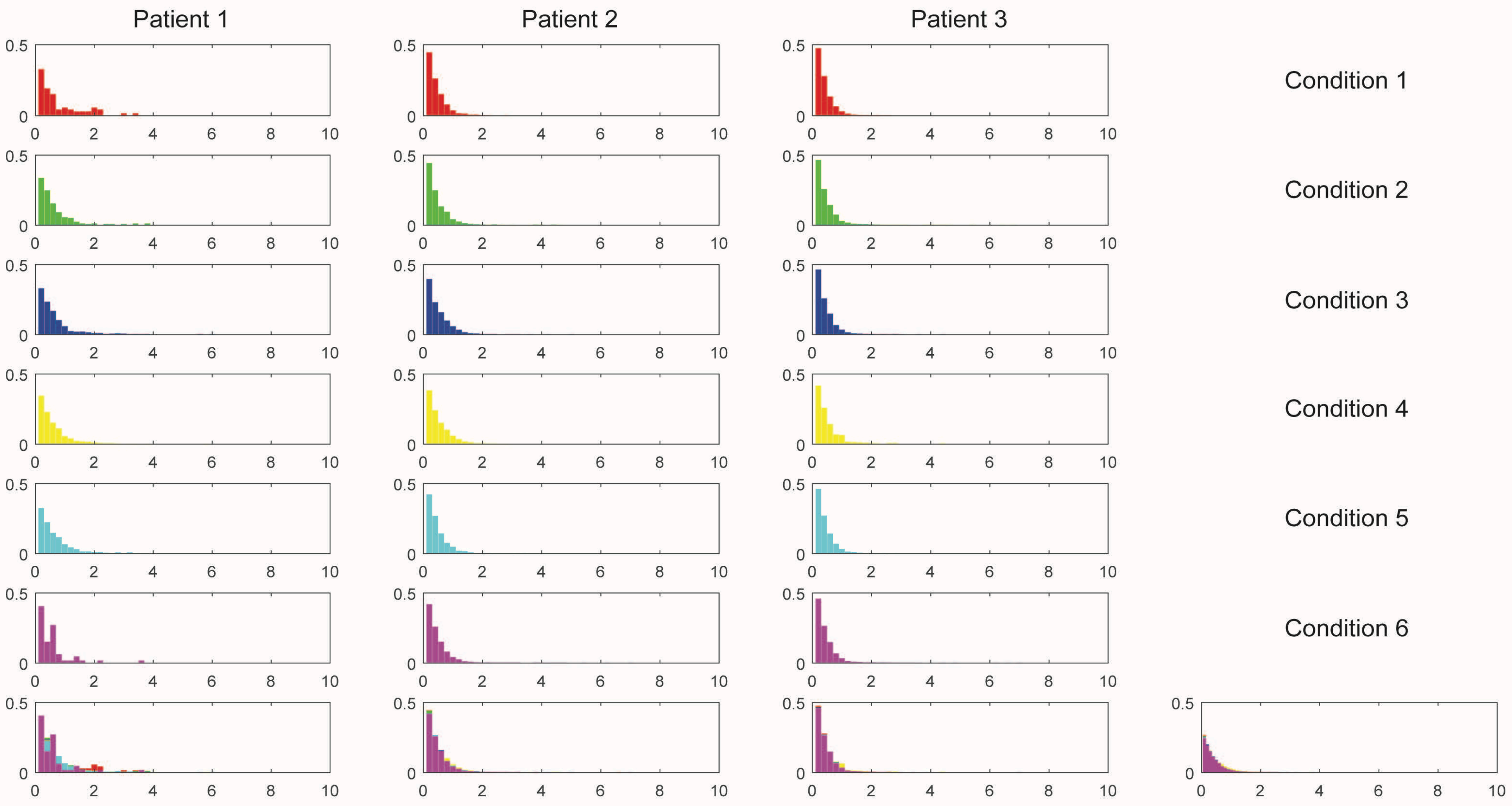

Figure S3.9: Histogram - CD20

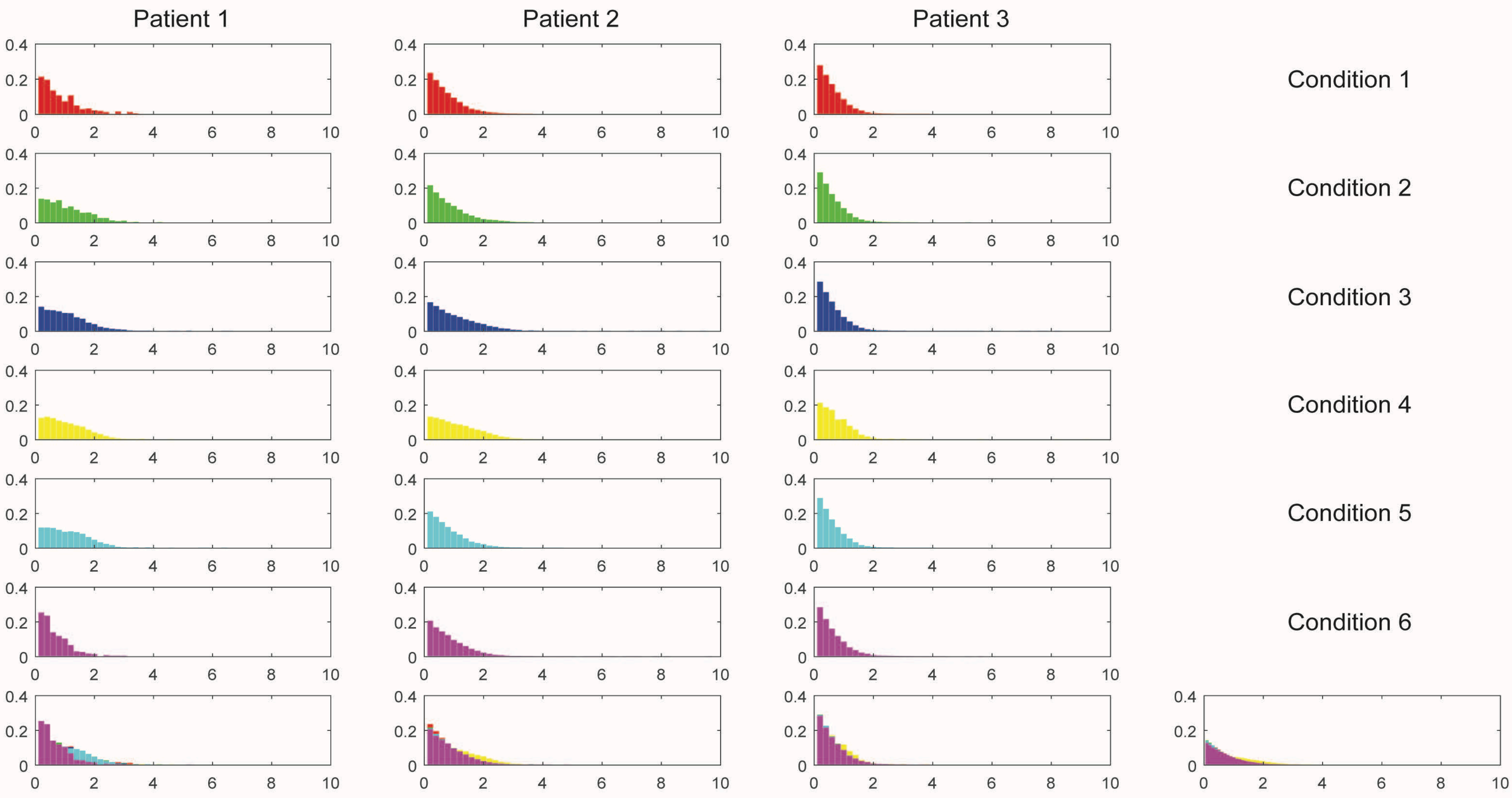

Figure S3.10: Histogram - CD24

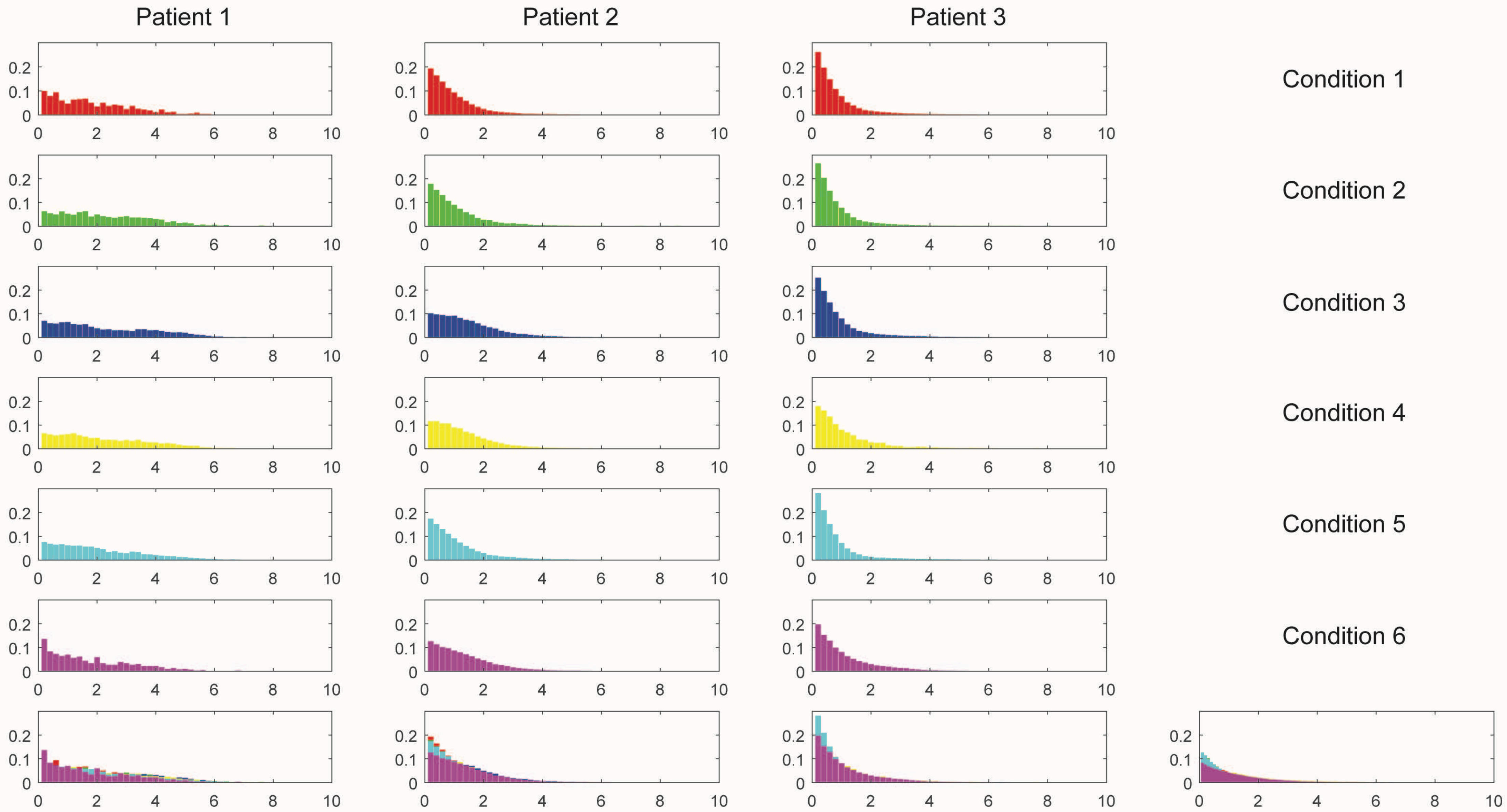

Figure S3.11: Histogram - CD25

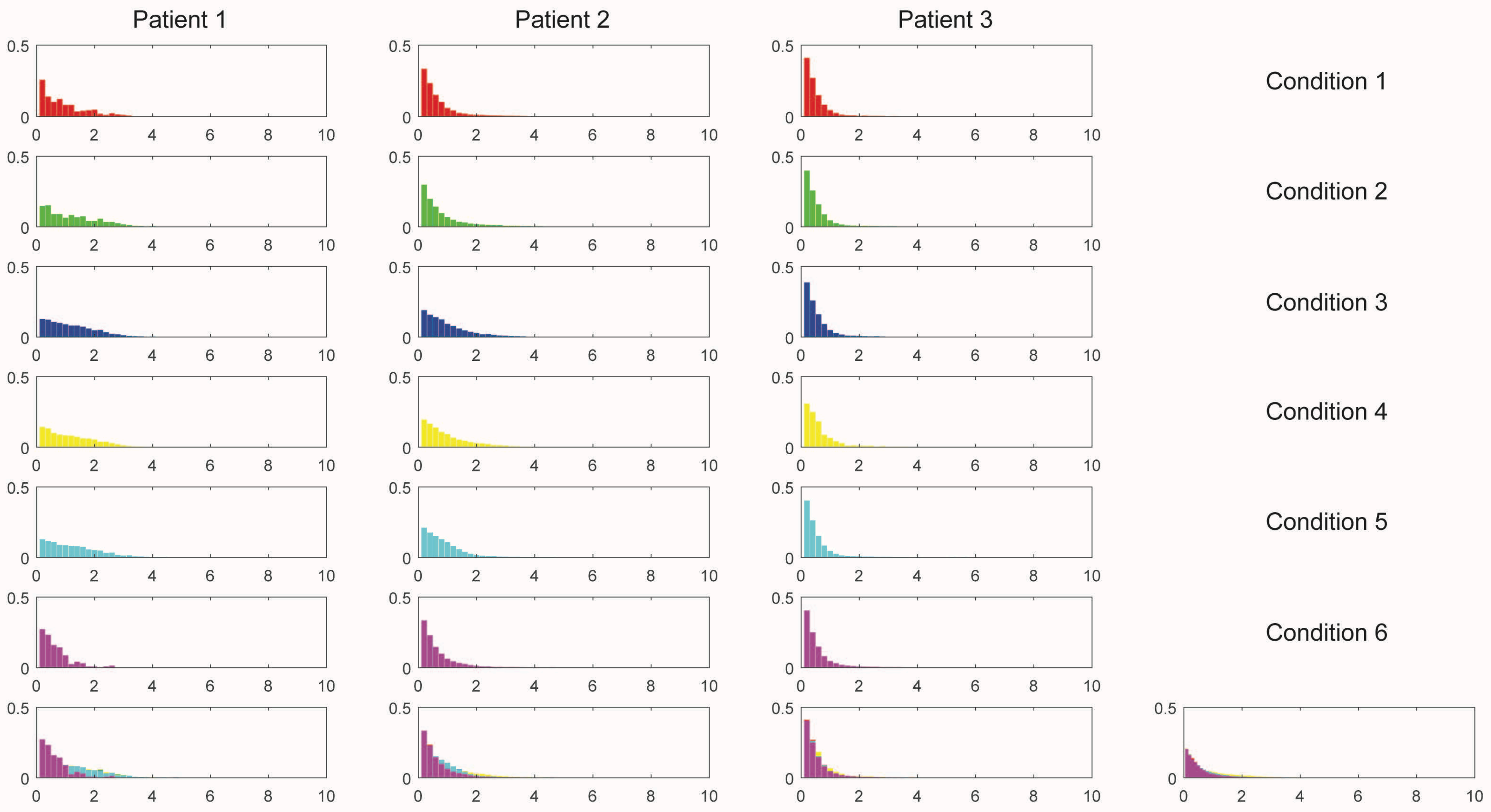

Figure S3.12: Histogram - CD34

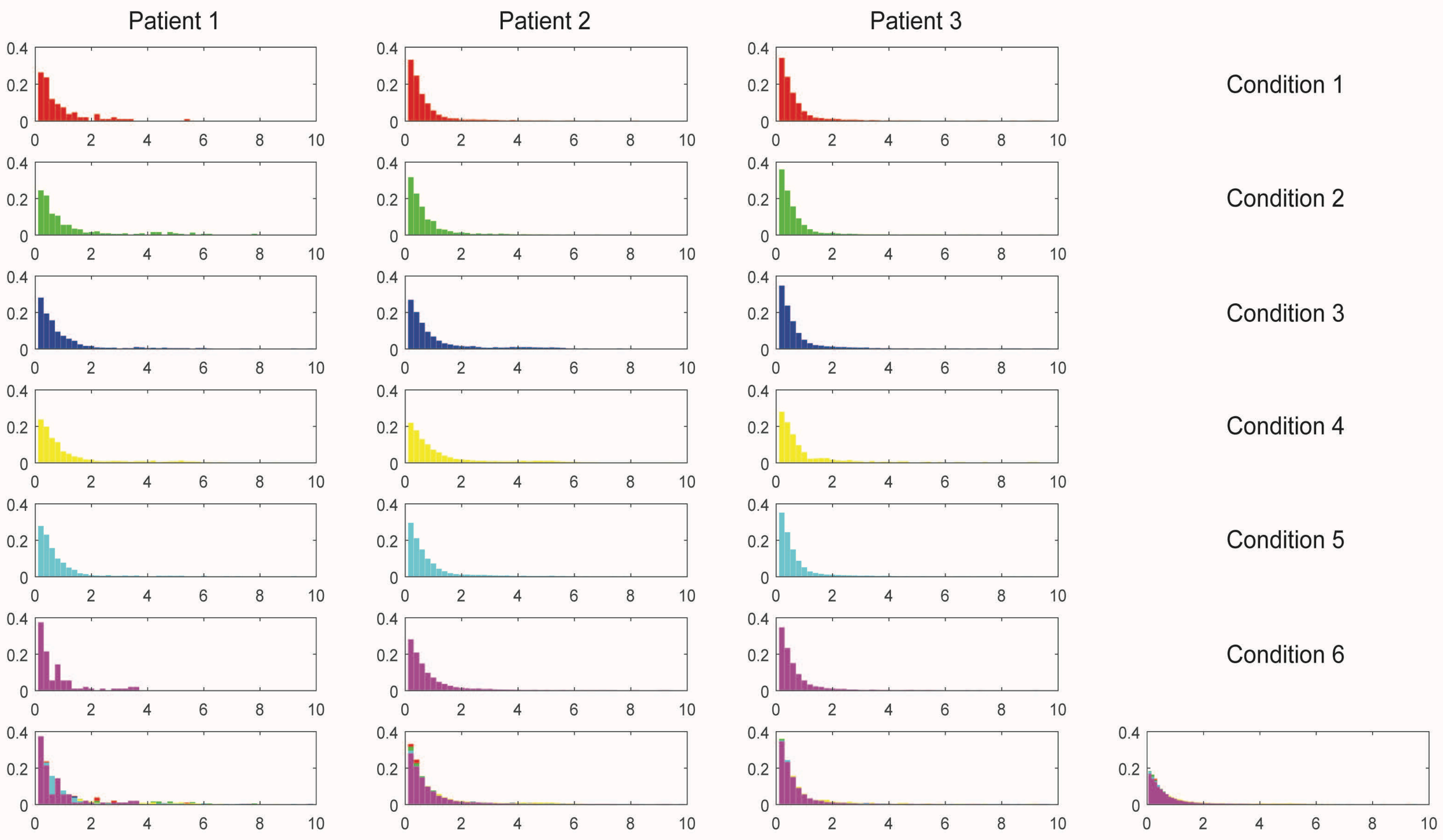

Figure S3.13: Histogram - CD44

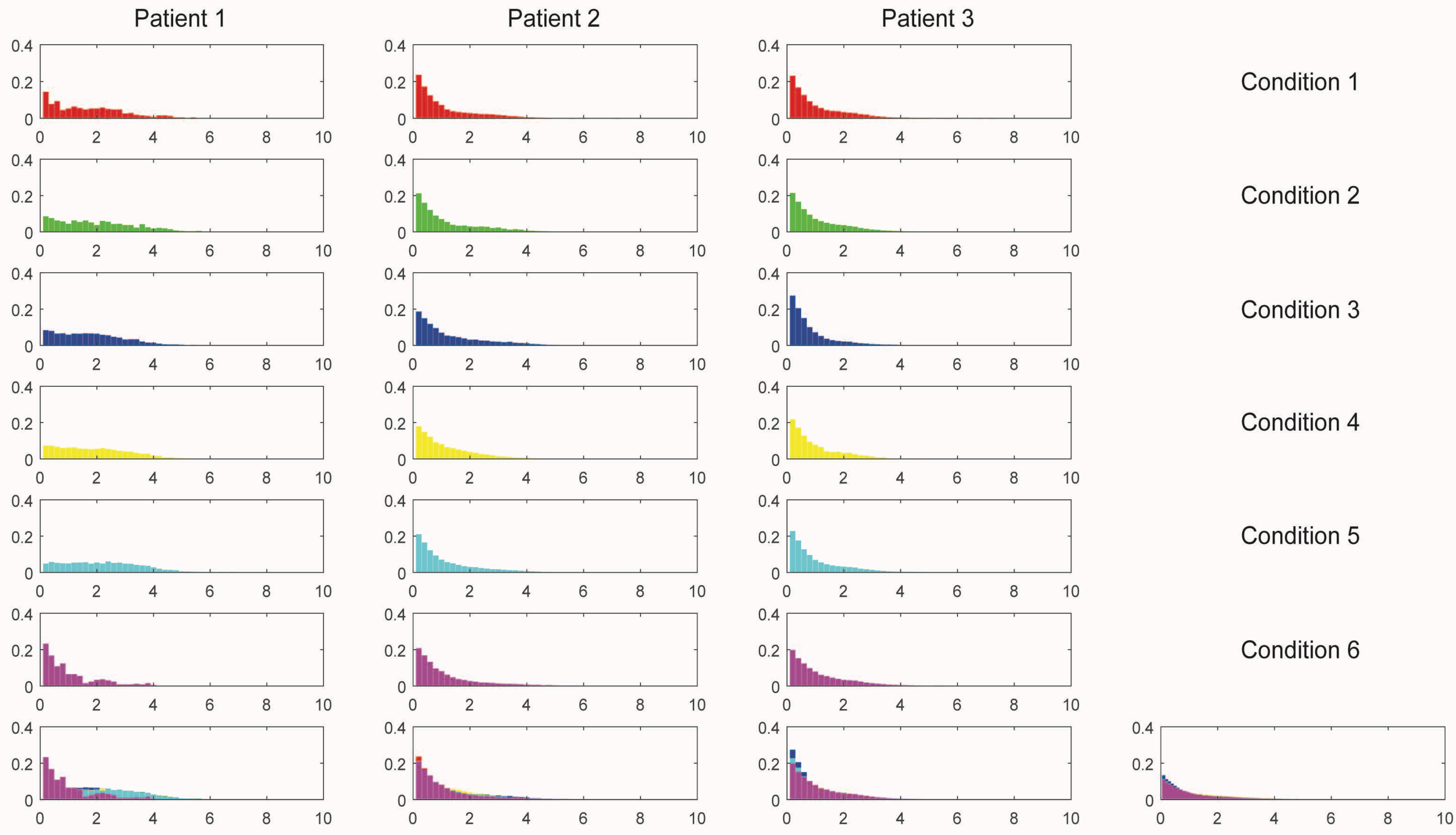

Figure S3.14: Histogram - CD45

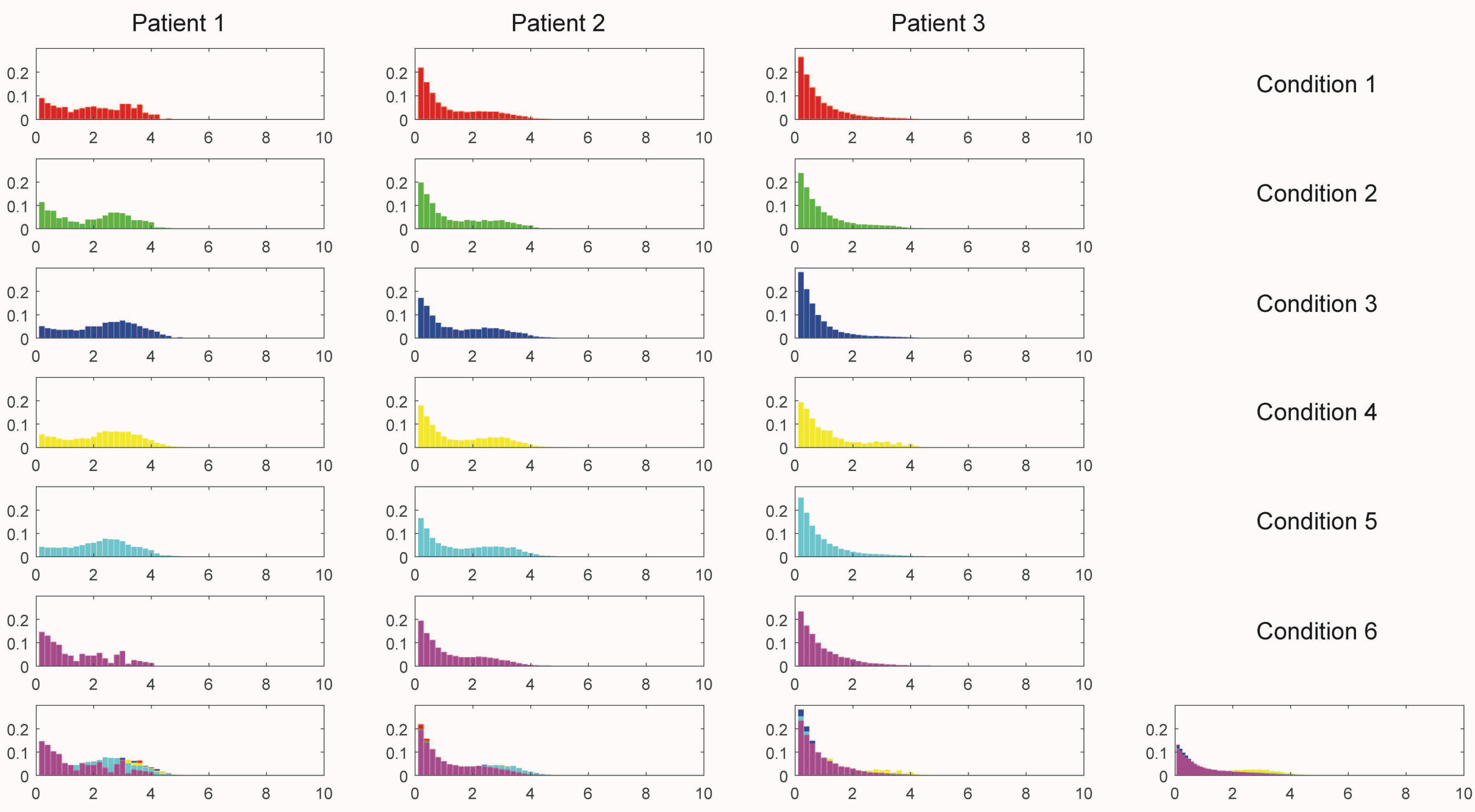

Figure S3.15: Histogram - CD45RO

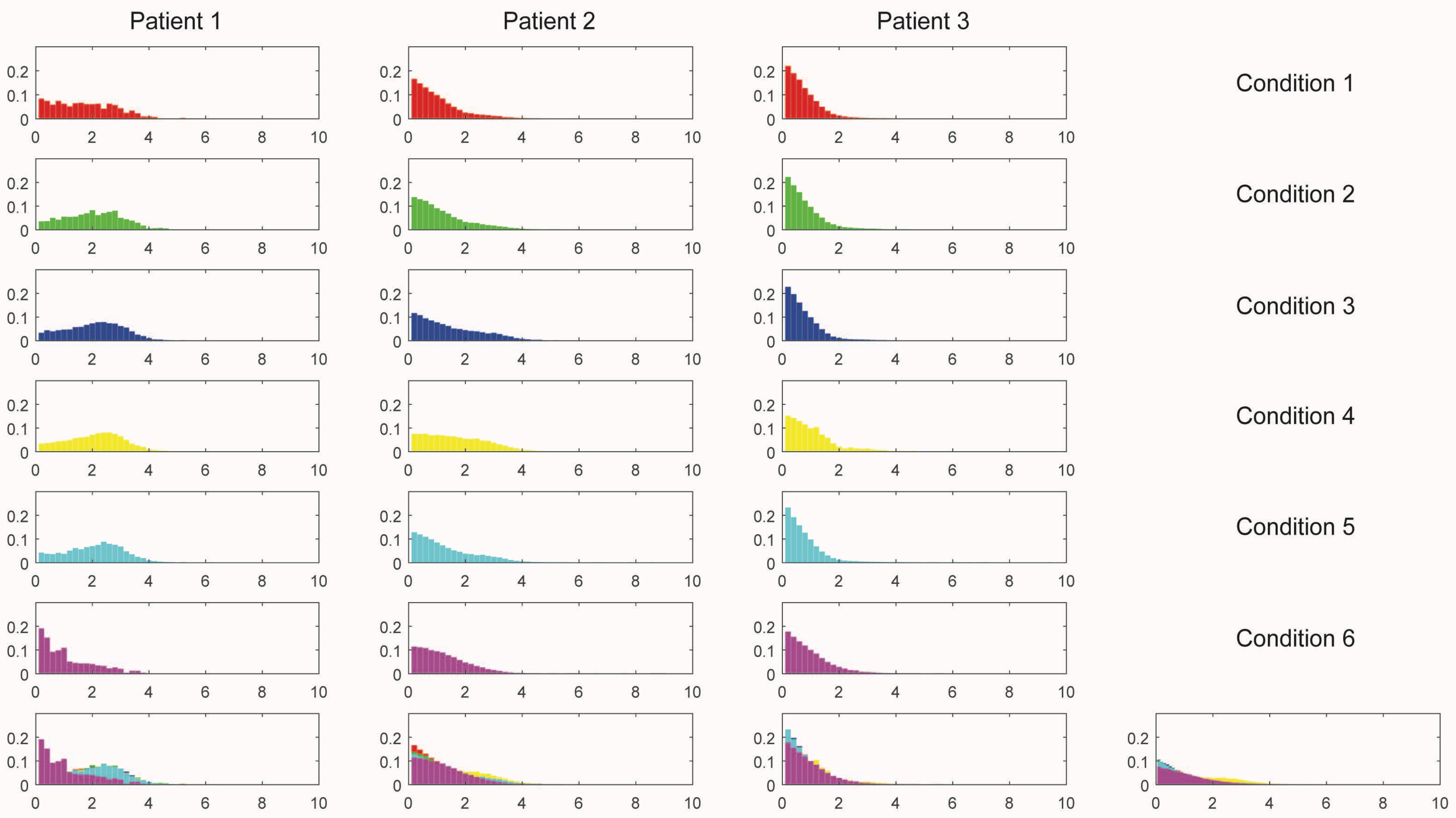

Figure S3.16: Histogram - CD47

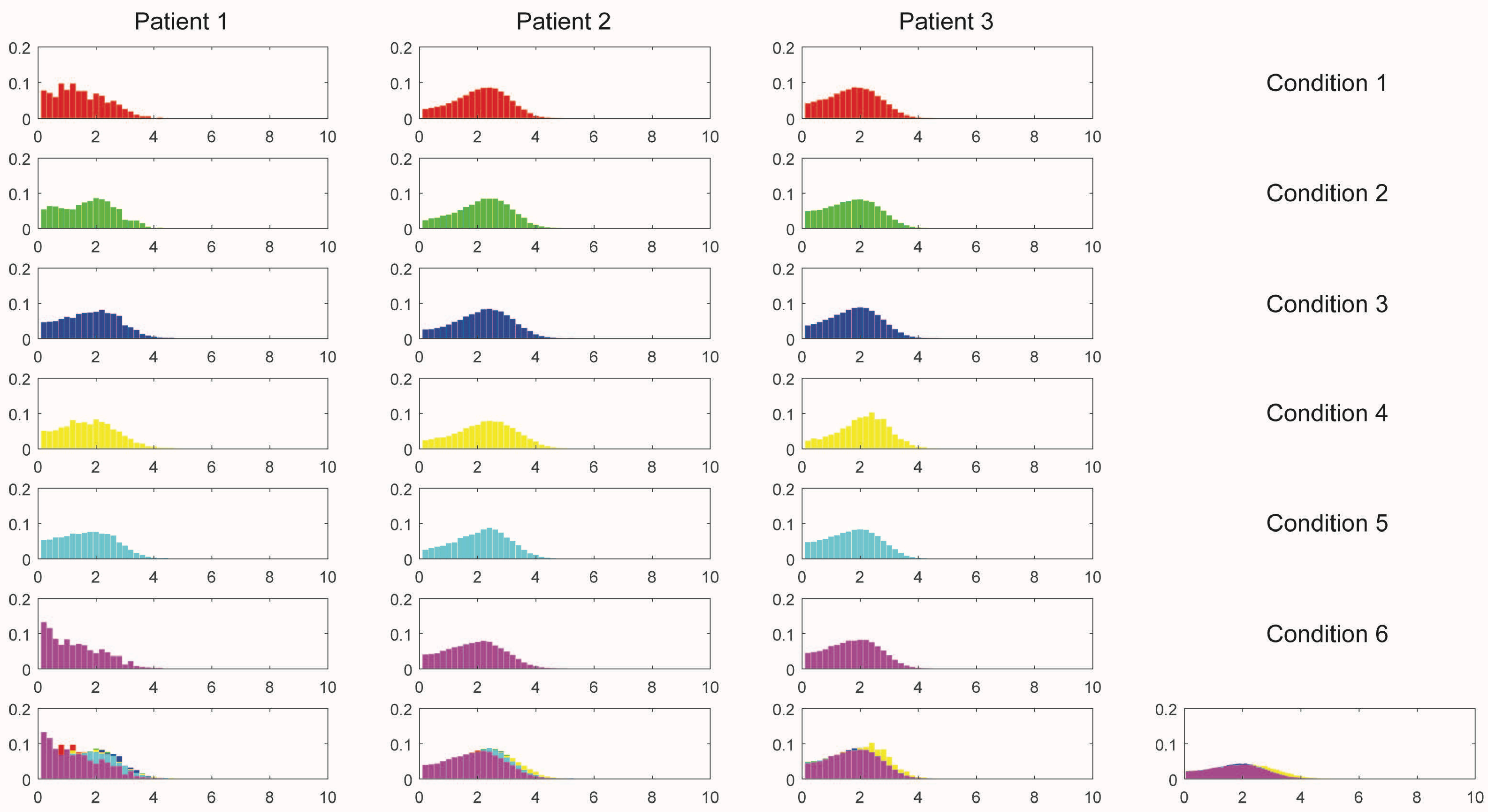

Figure S3.17: Histogram - CD56

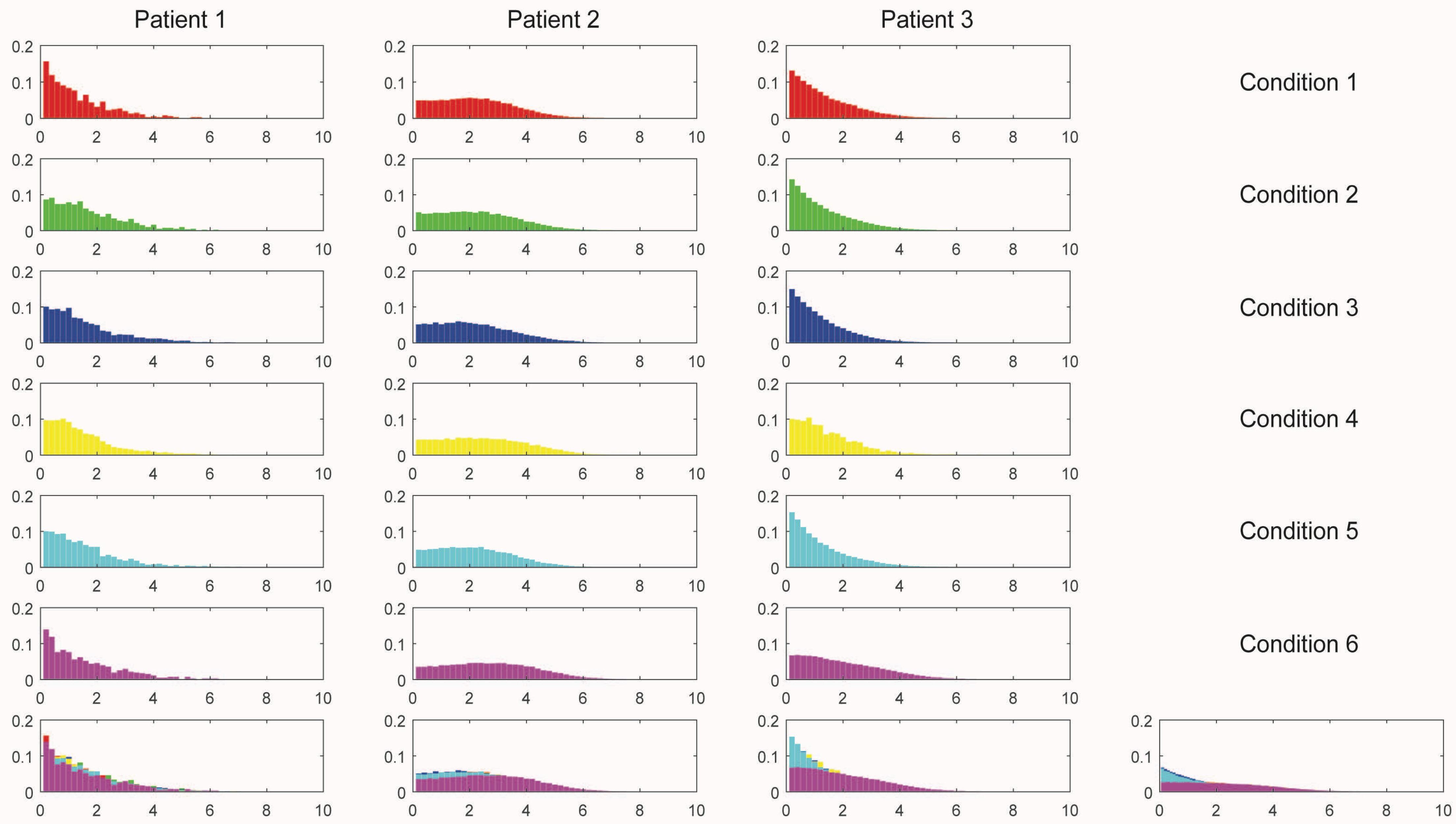

Figure S3.18: Histogram - CD73

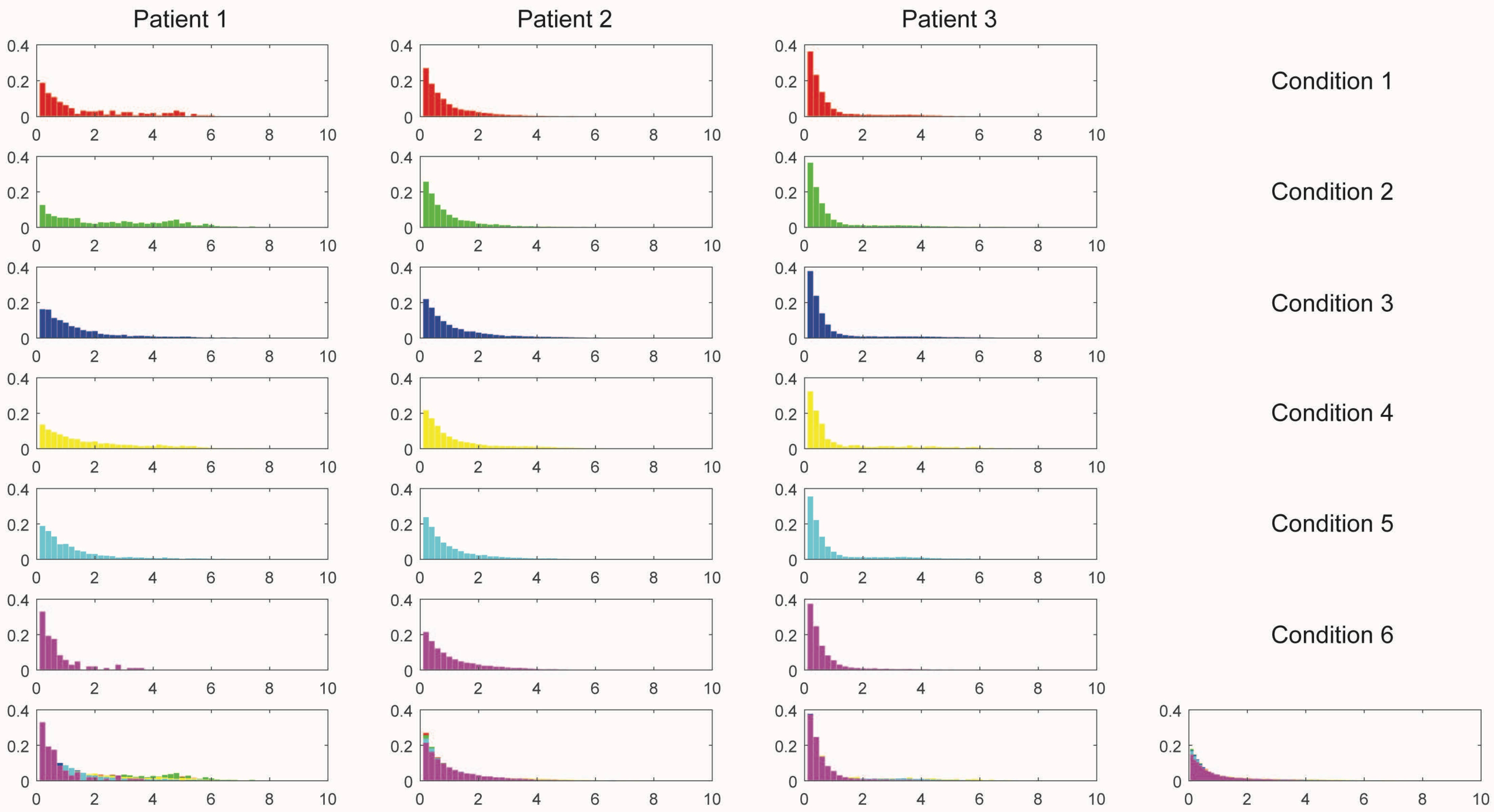

Figure S3.19: Histogram - CD103

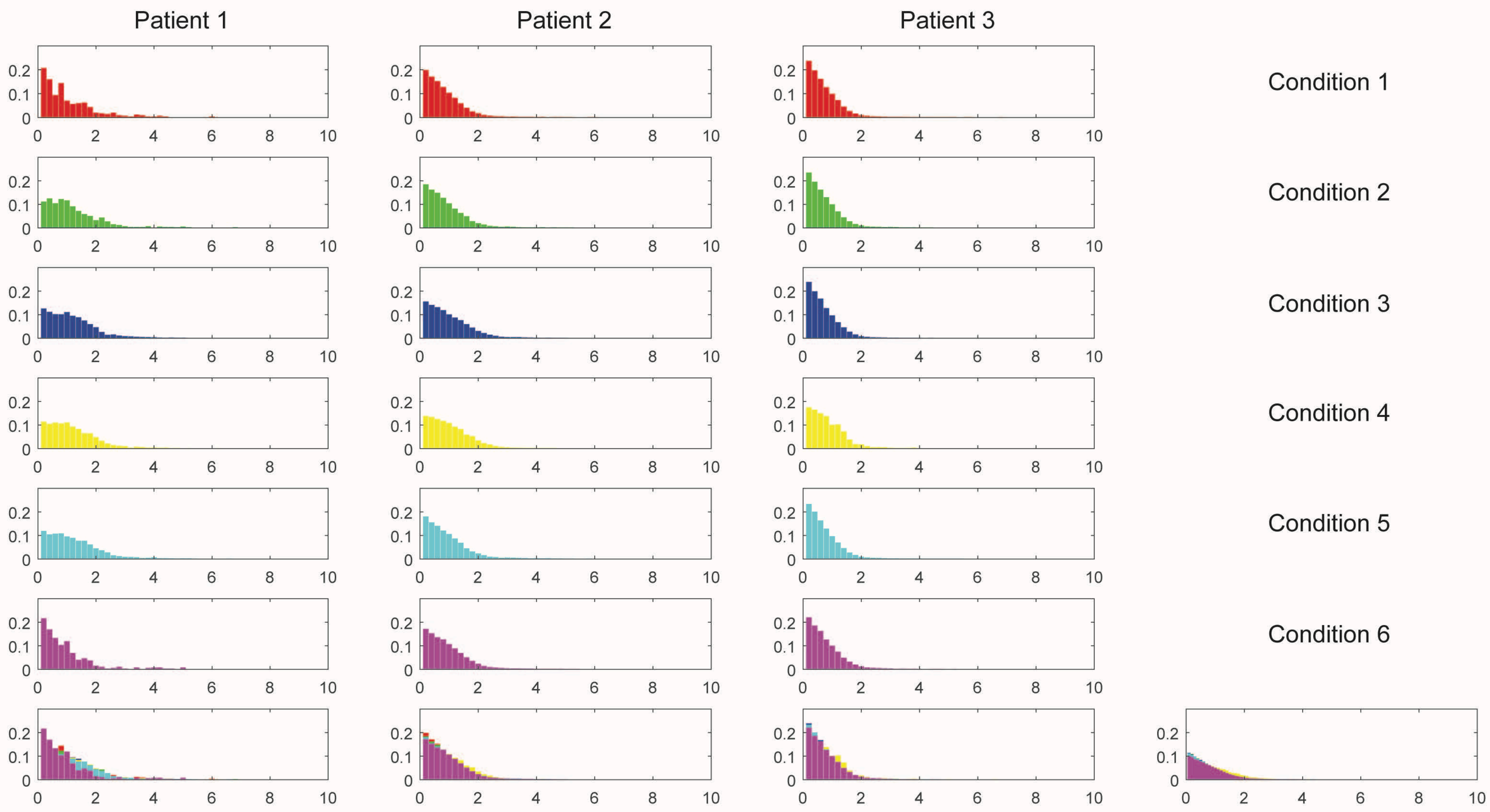

Figure S3.20: Histogram - CD117

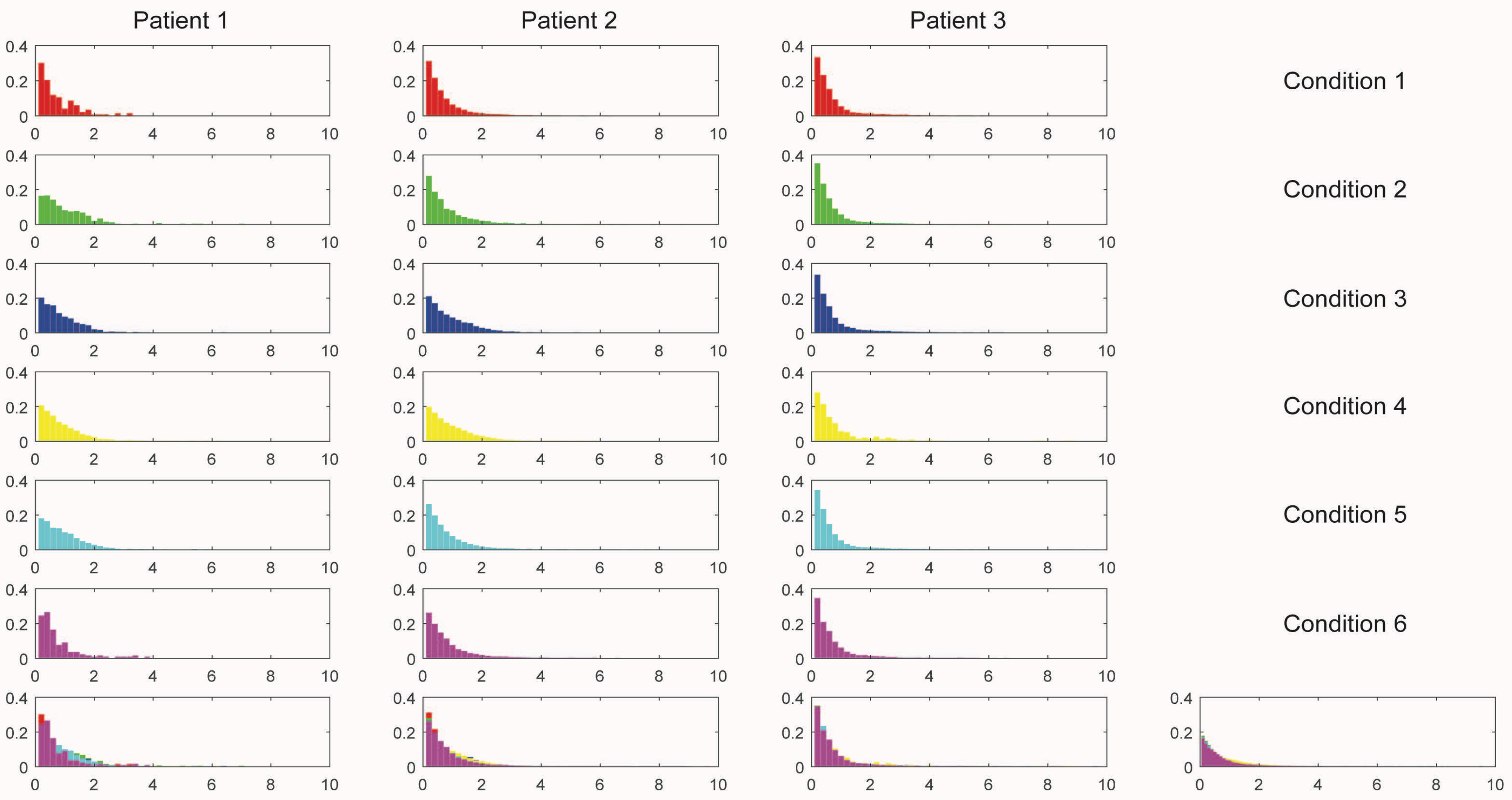

Figure S3.21: Histogram - CD133

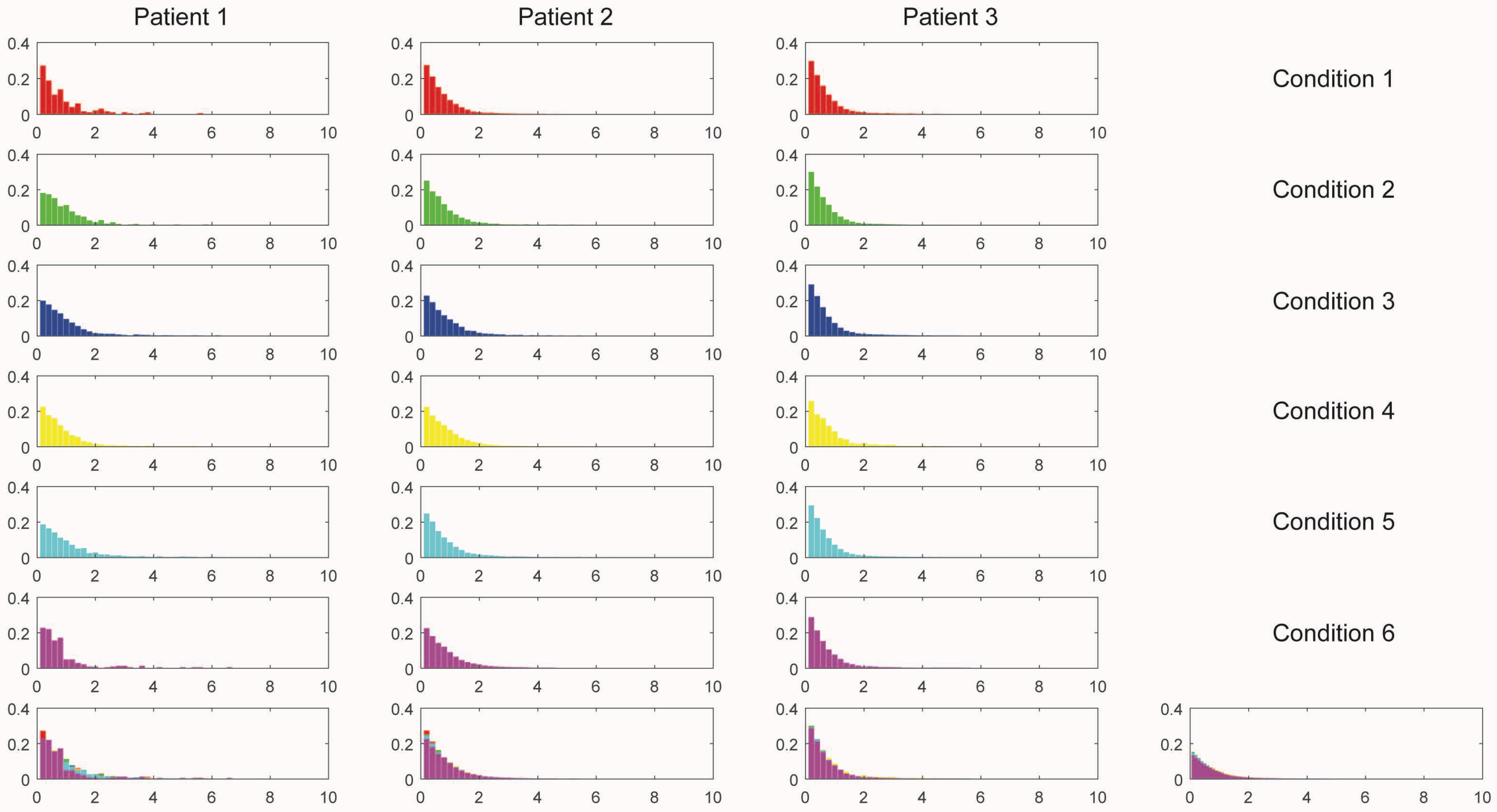

Figure S3.22: Histogram - CTLA4

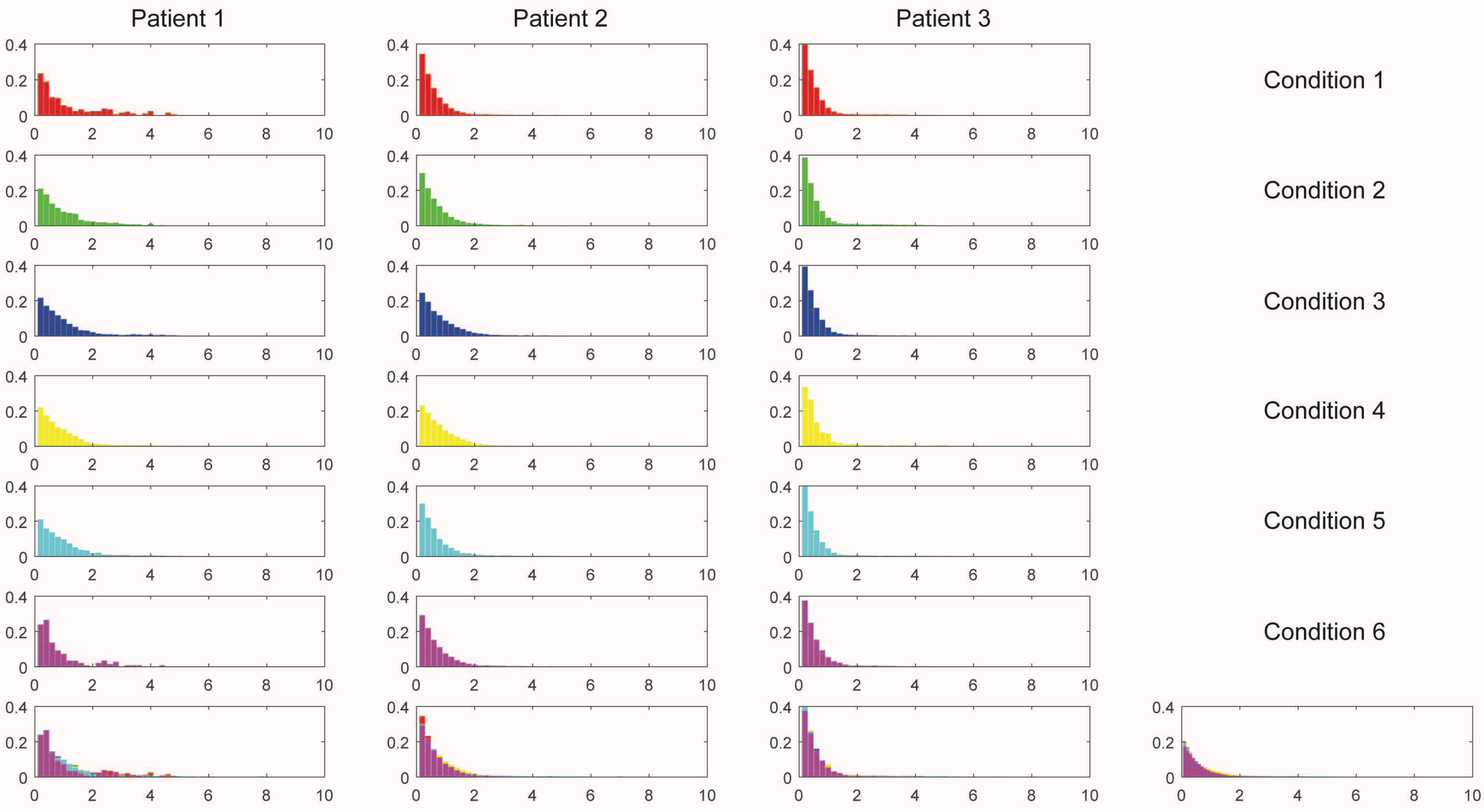

Figure S3.23: Histogram - EpCAM

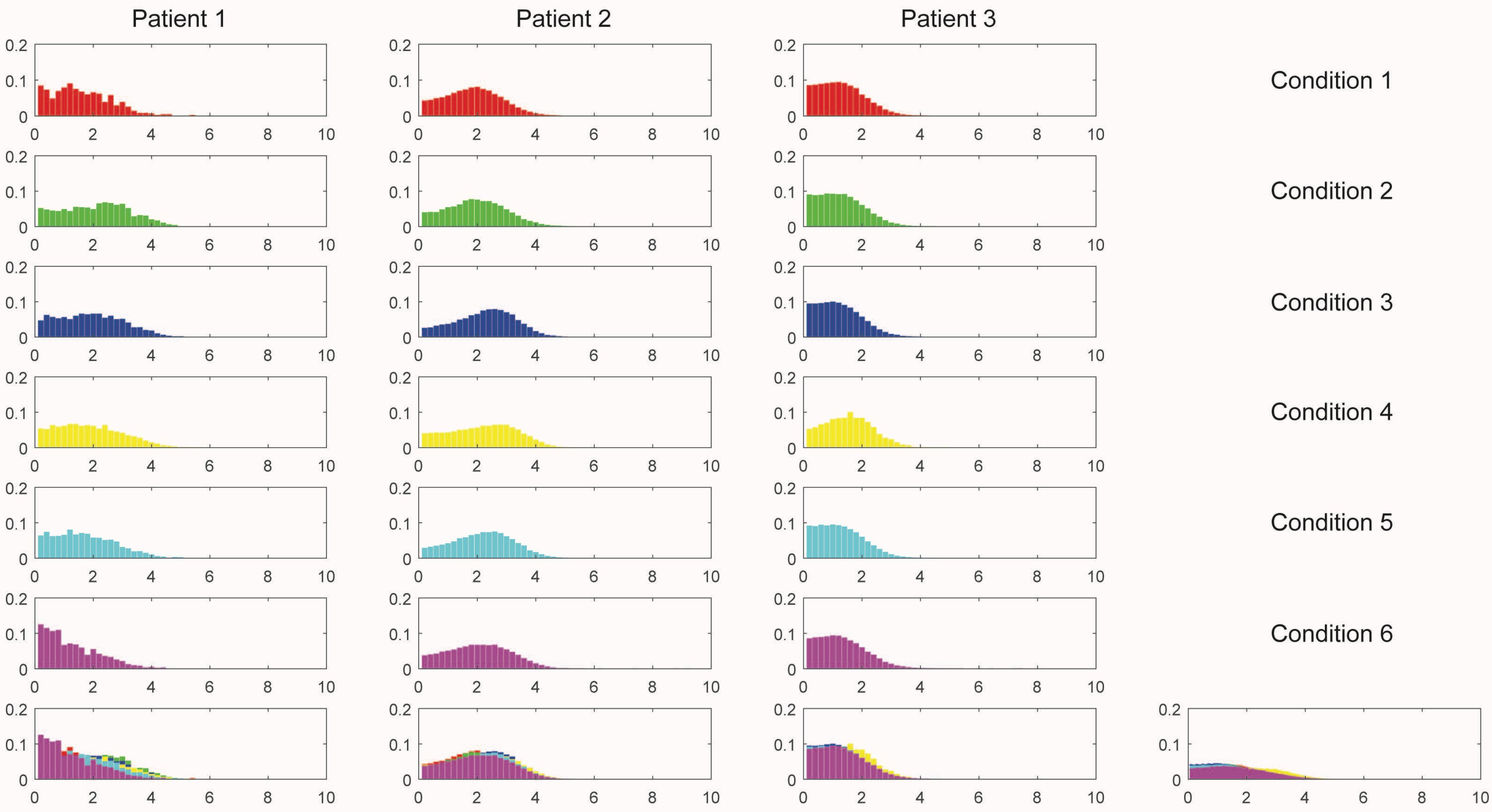

Figure S3.24: Histogram - FAPa

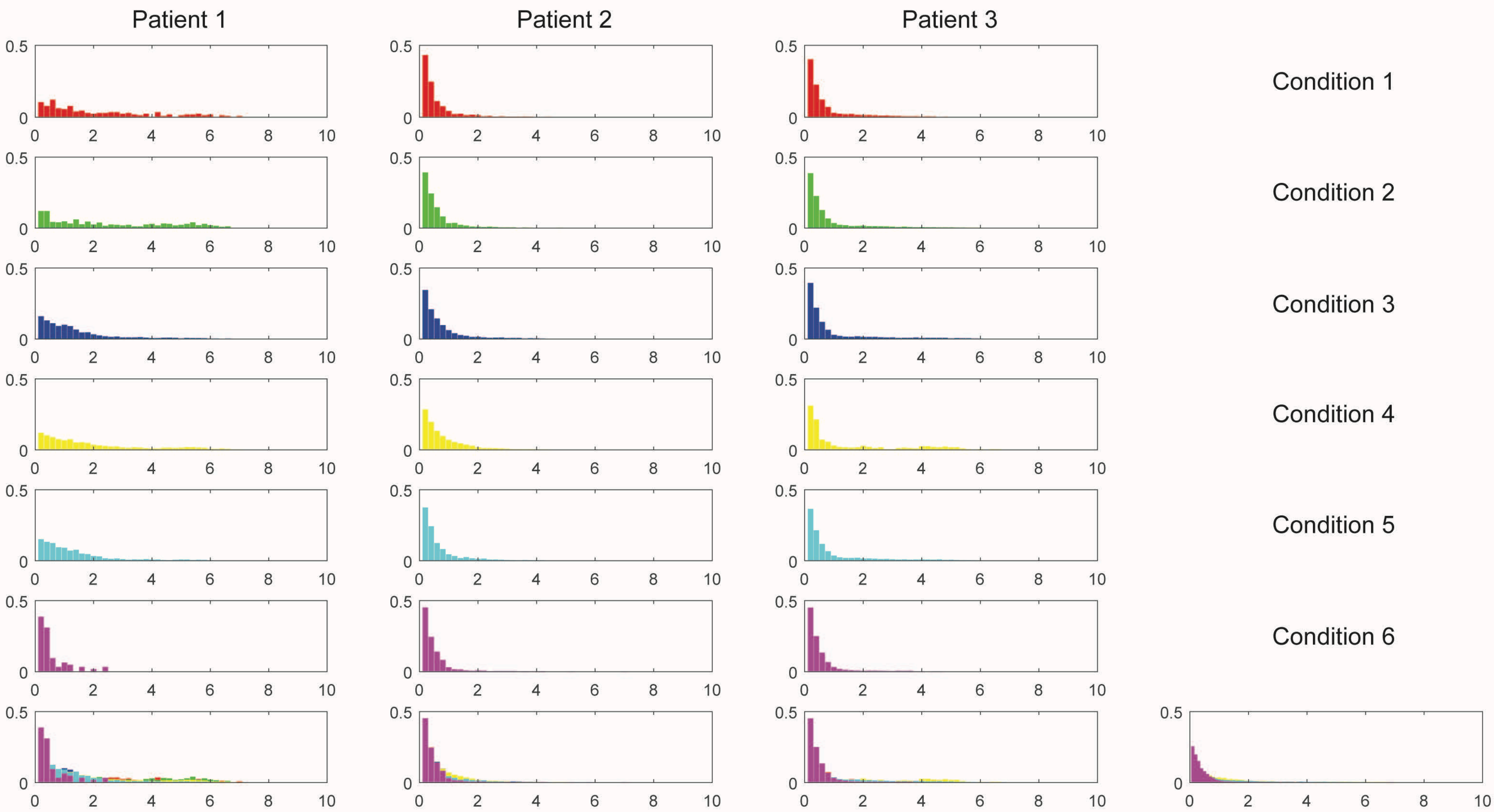

Figure S3.25: Histogram - FOLR1

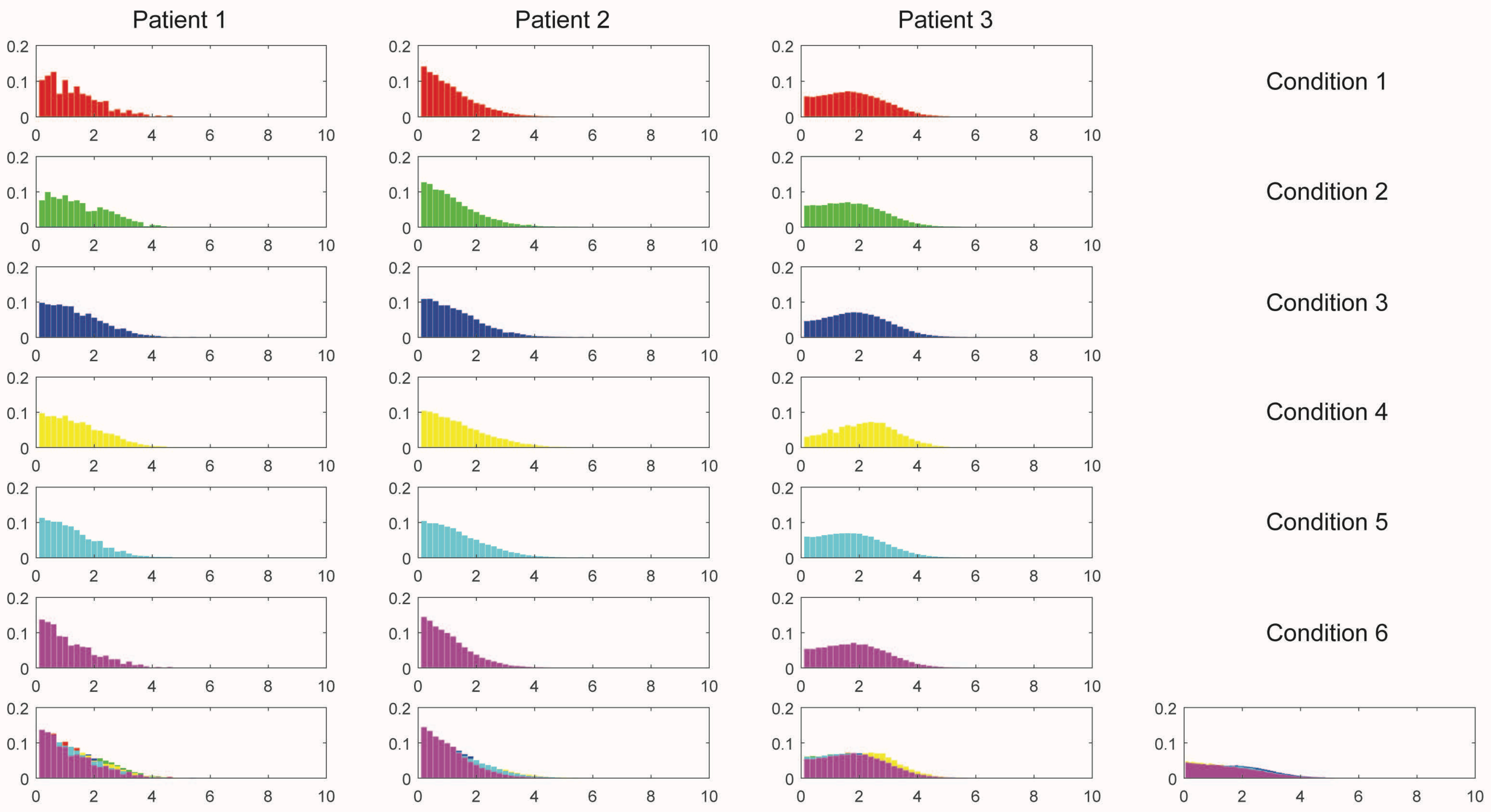

Figure S3.26: Histogram - Foxp3

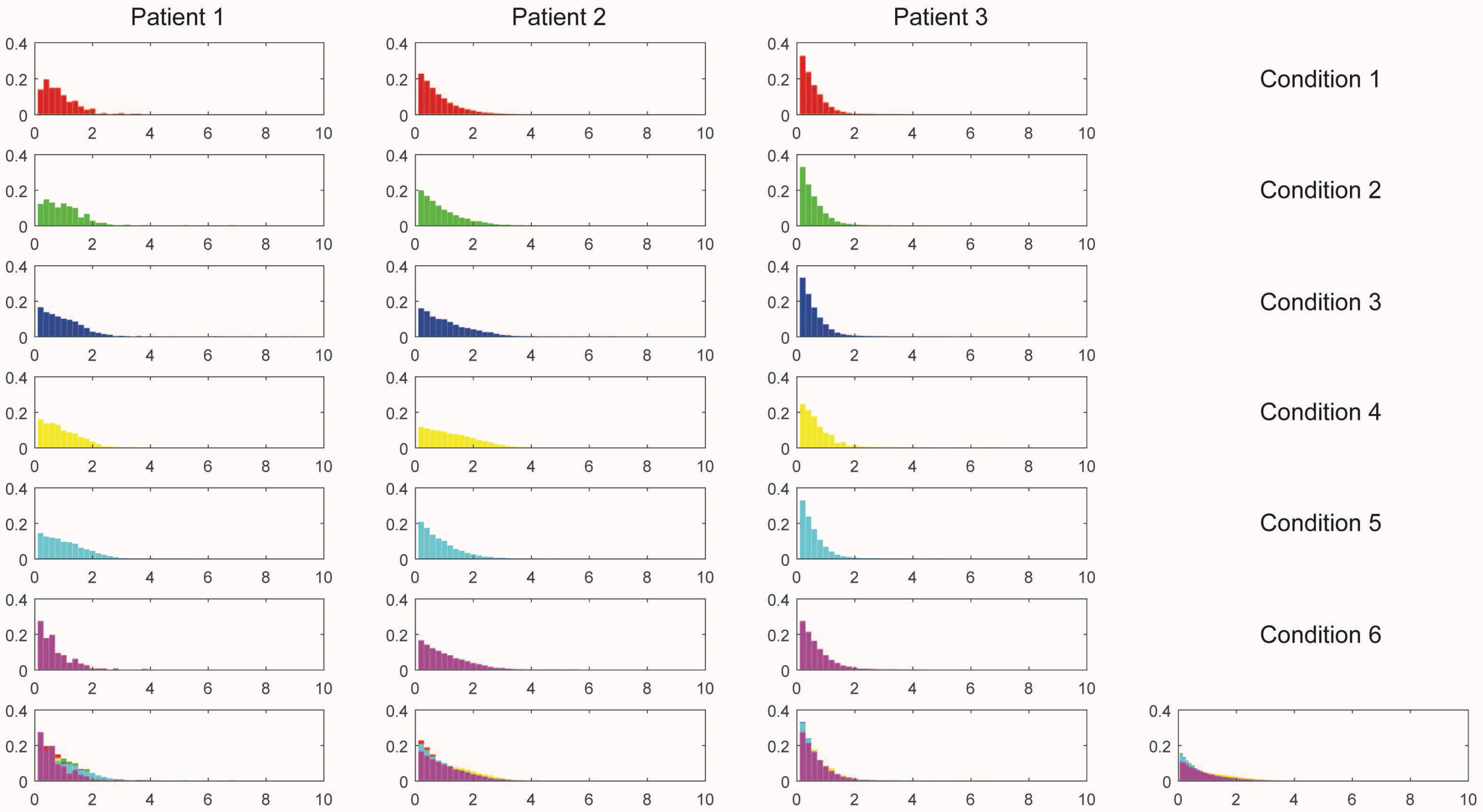

Figure S3.27: Histogram - HLA-DR

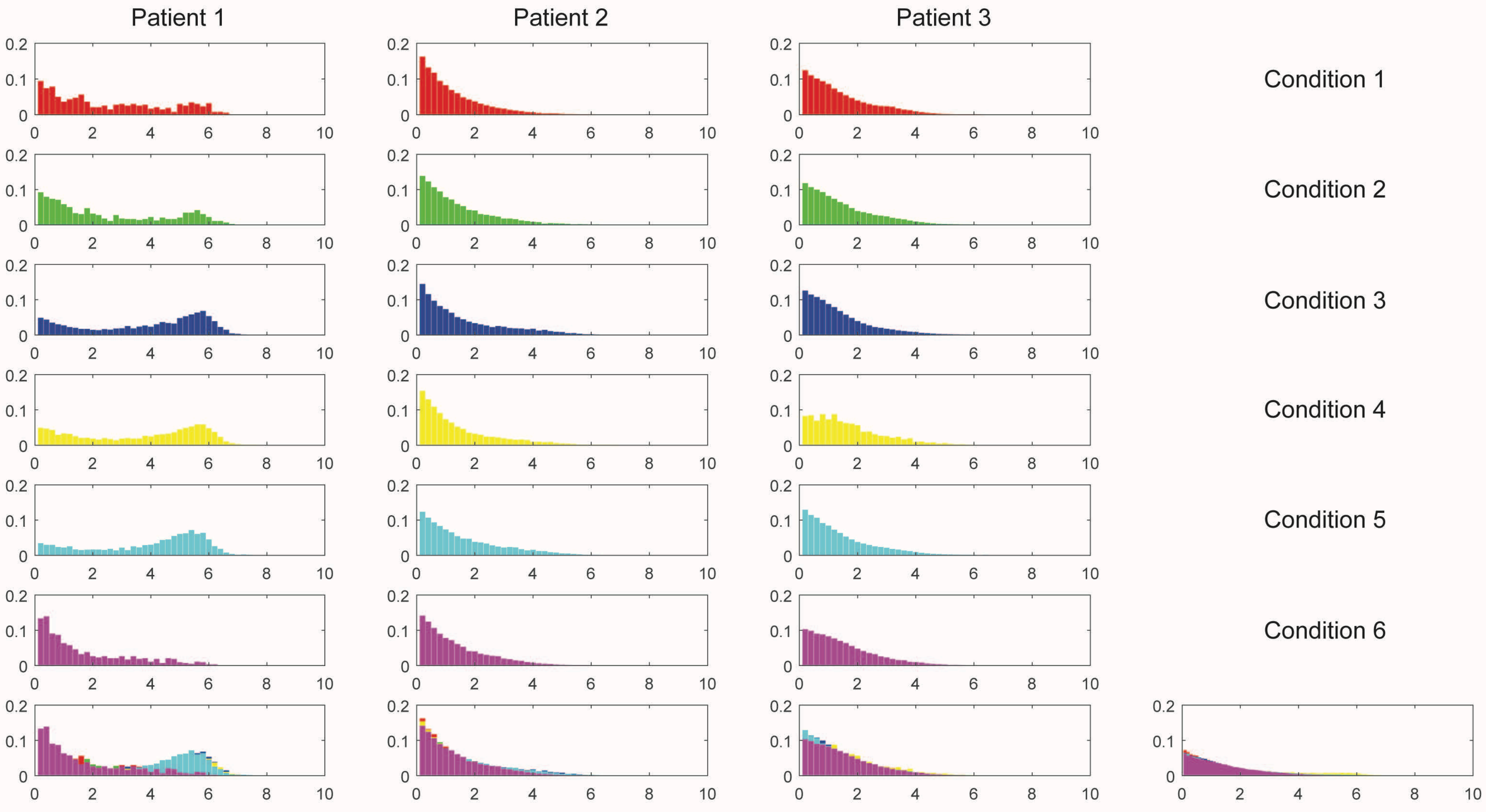

Figure S3.28: Histogram - IFNg

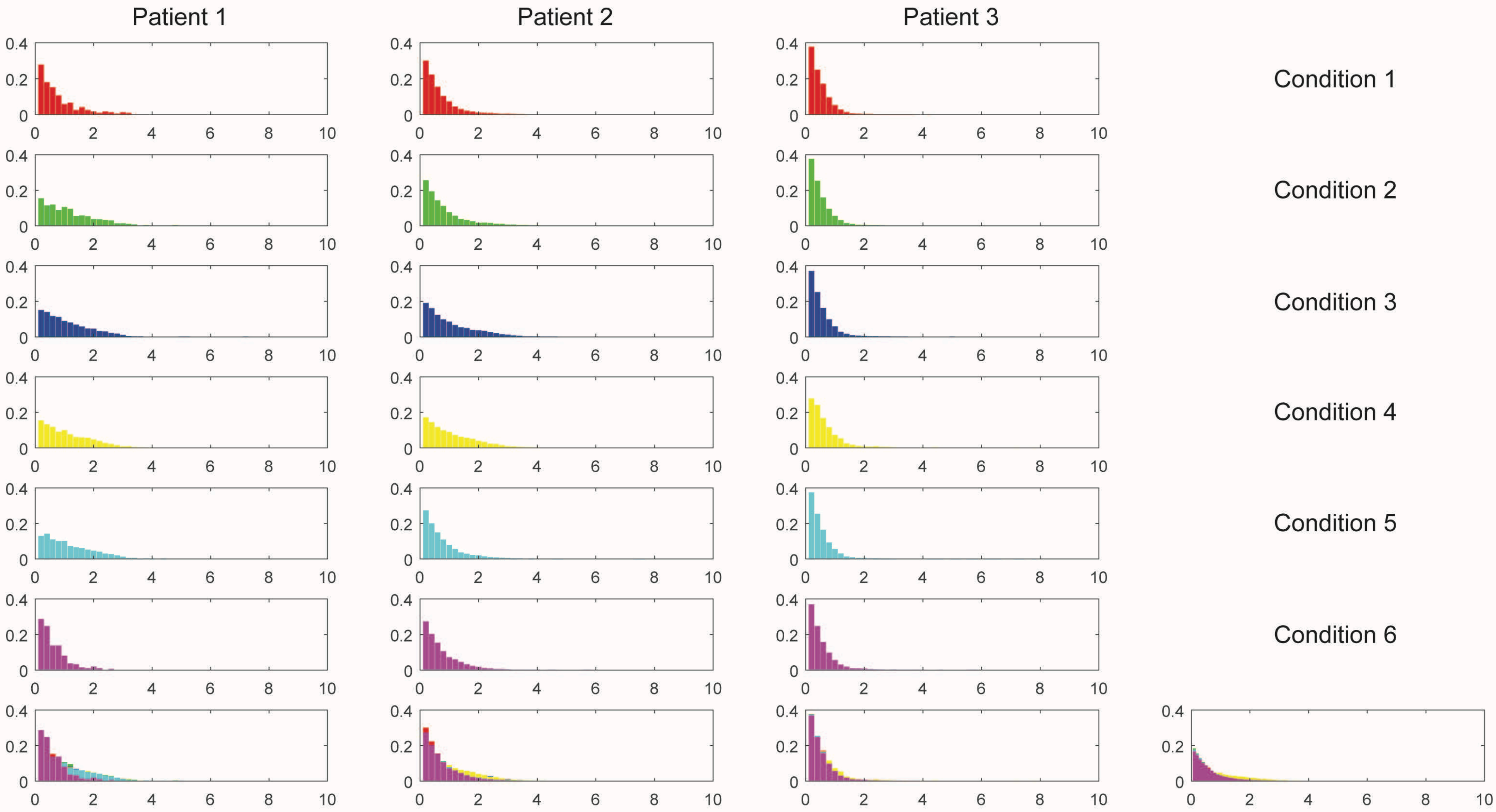

Figure S3.29: Histogram - Lag3

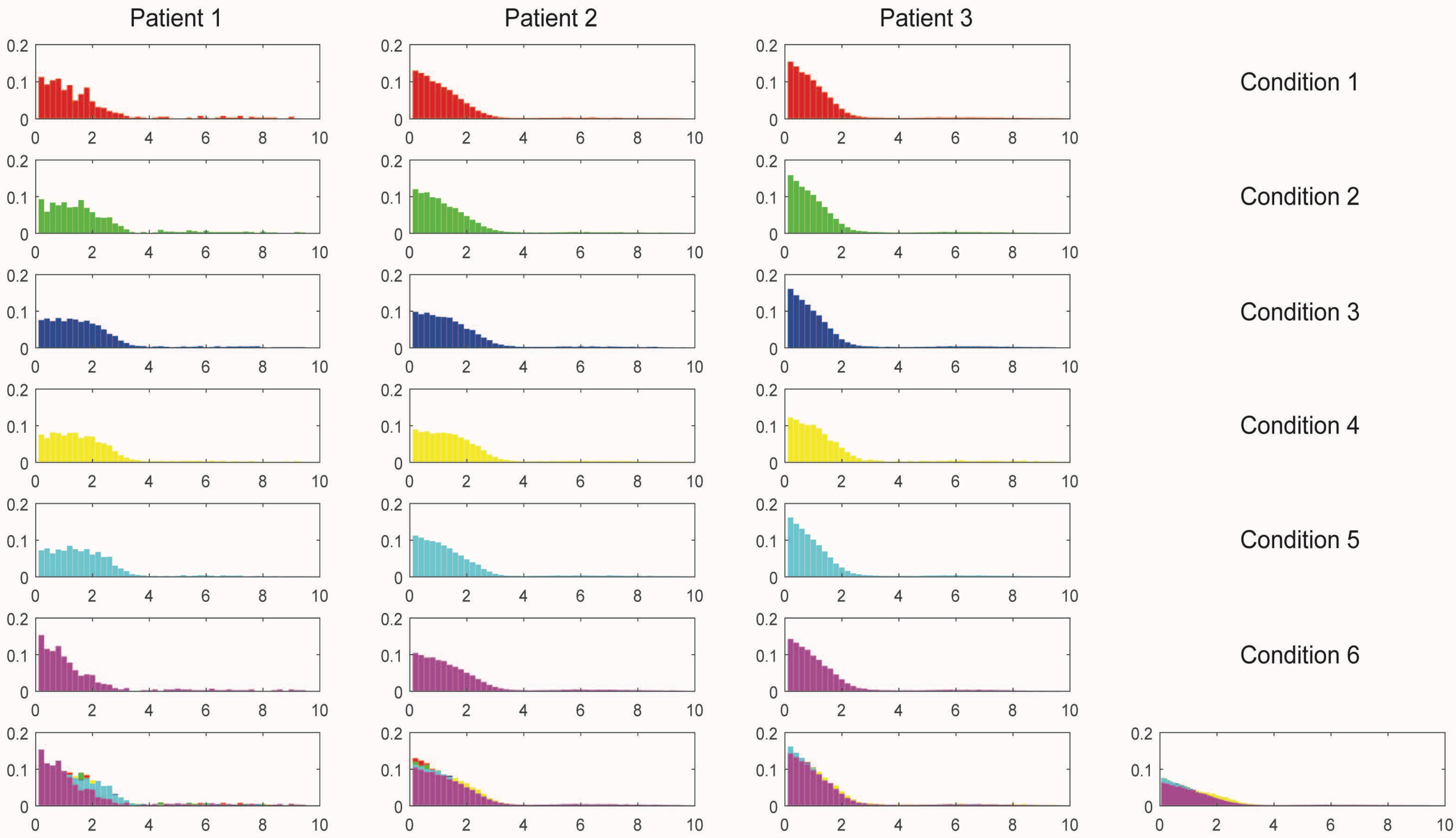

Figure S3.30: Histogram - OX40

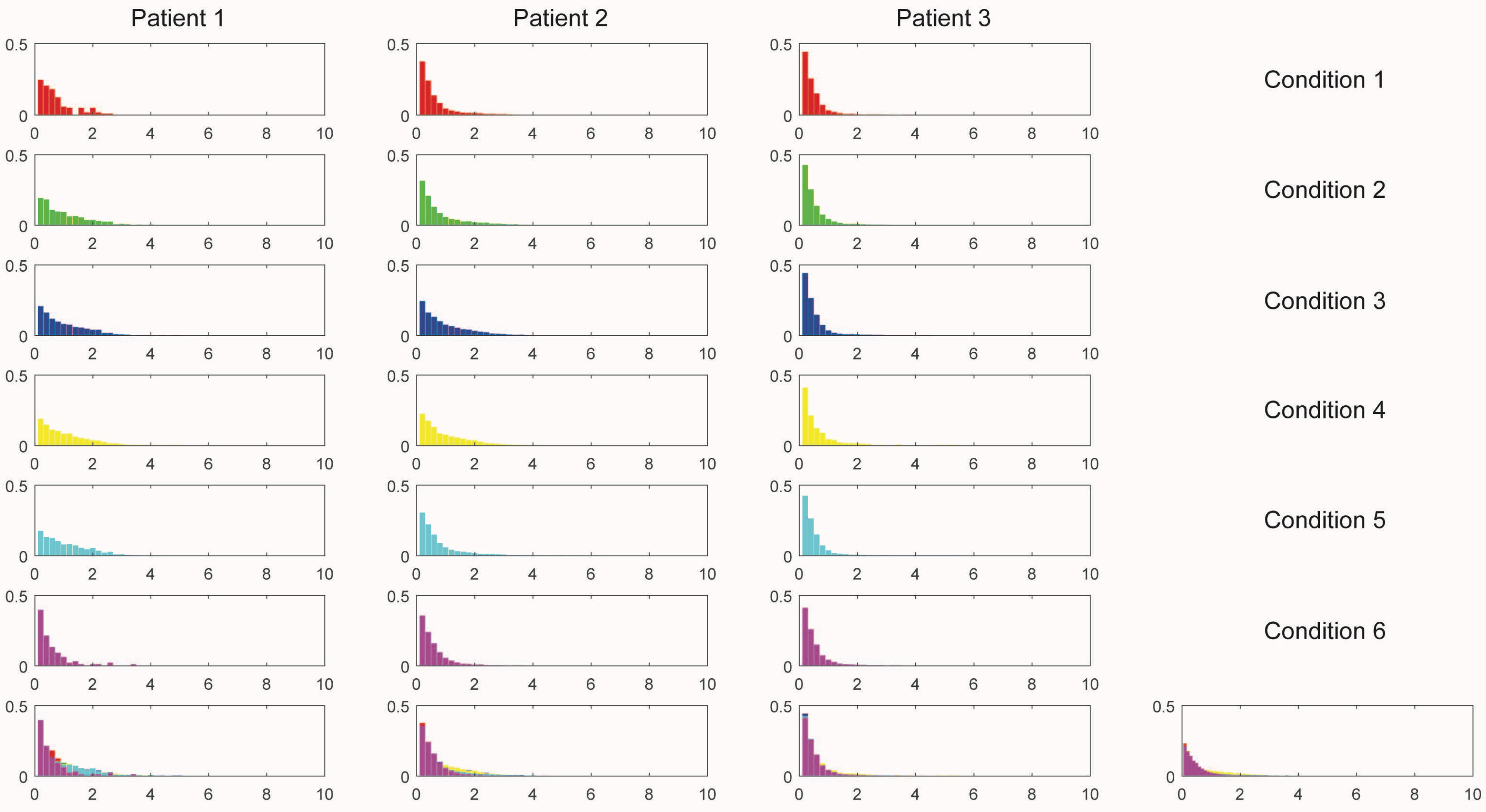

Figure S3.31: Histogram - PD1

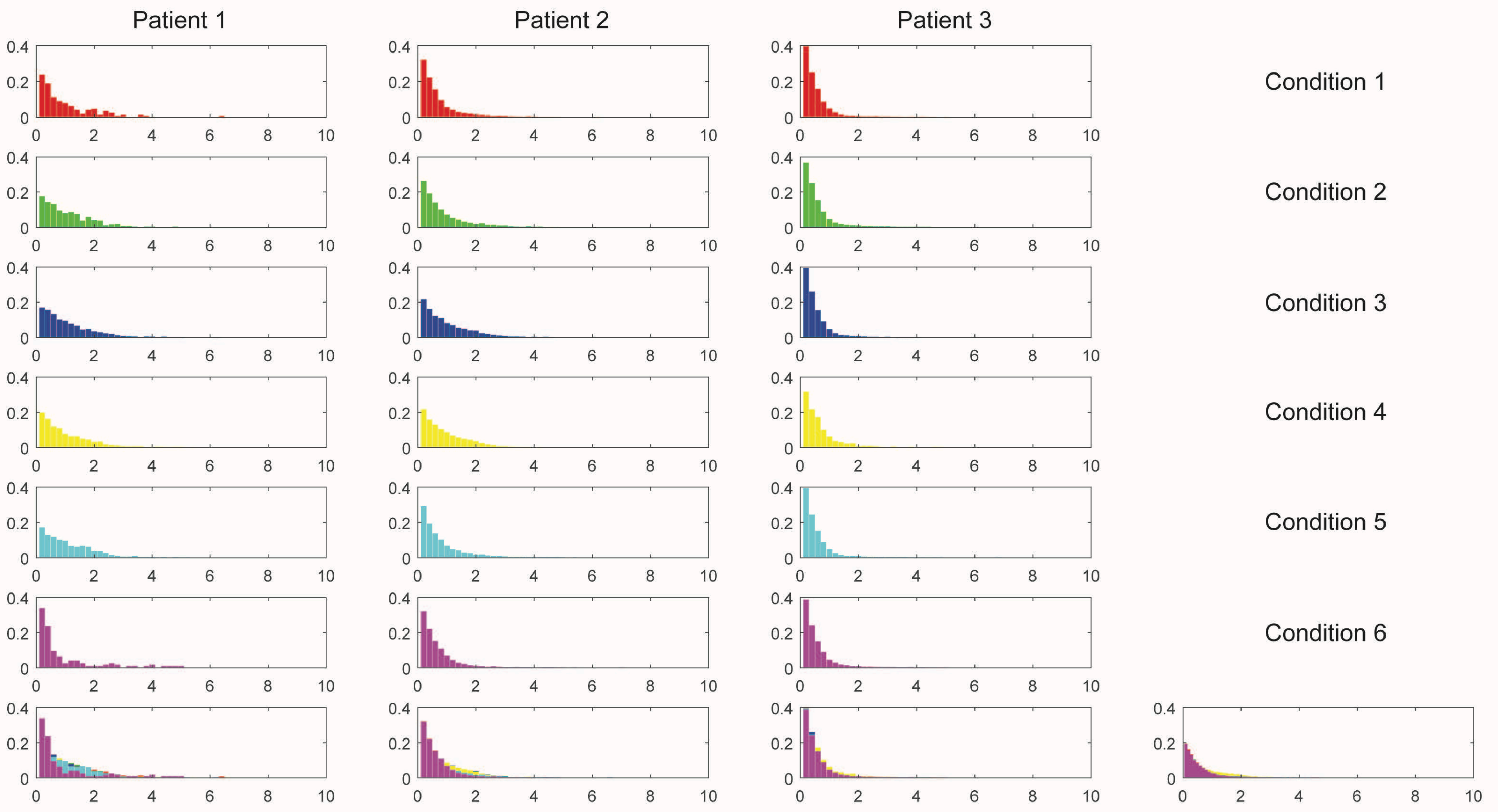

Figure S3.32: Histogram - PDGFRb

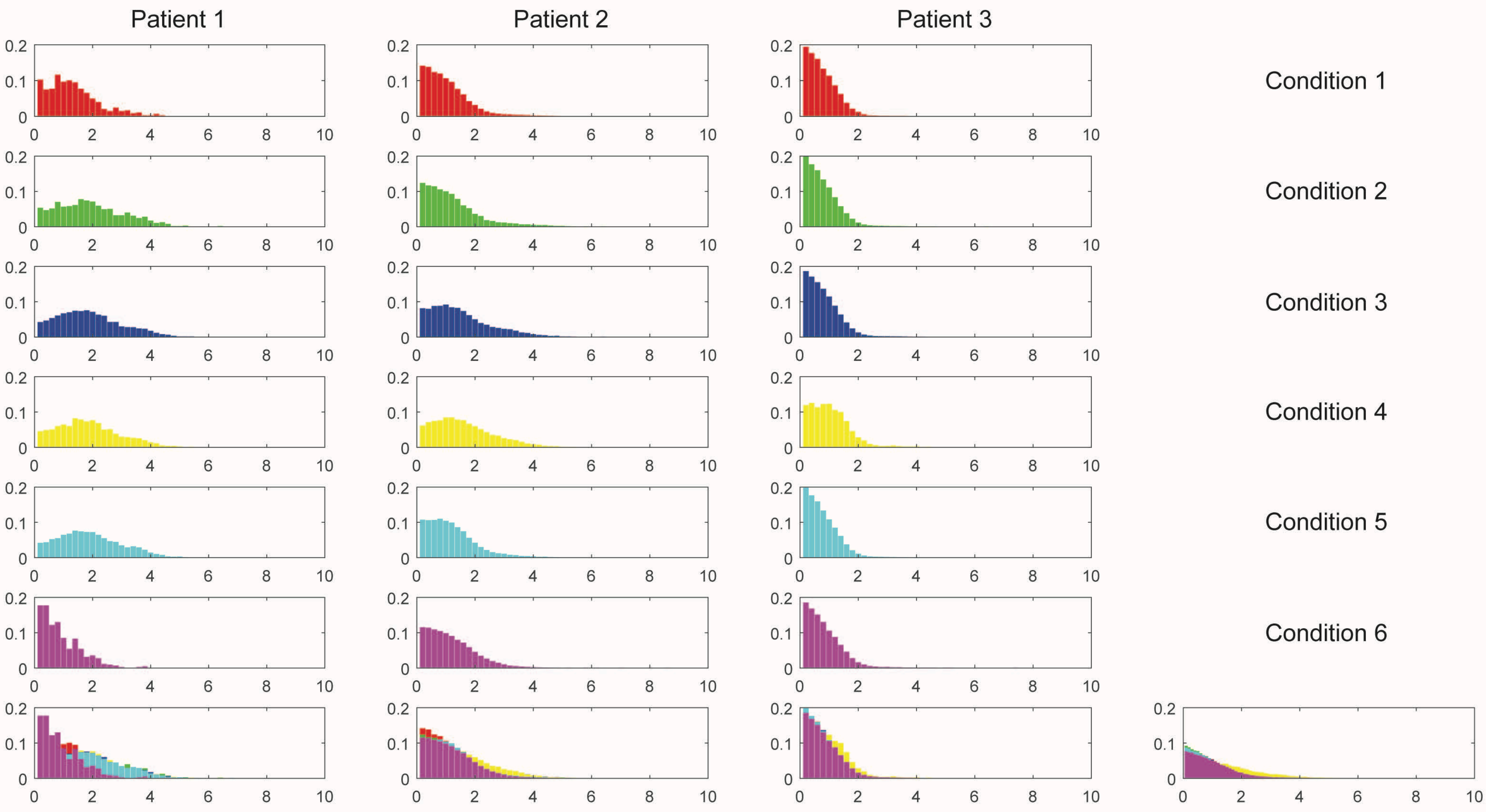

Figure S3.33: Histogram - PDL1

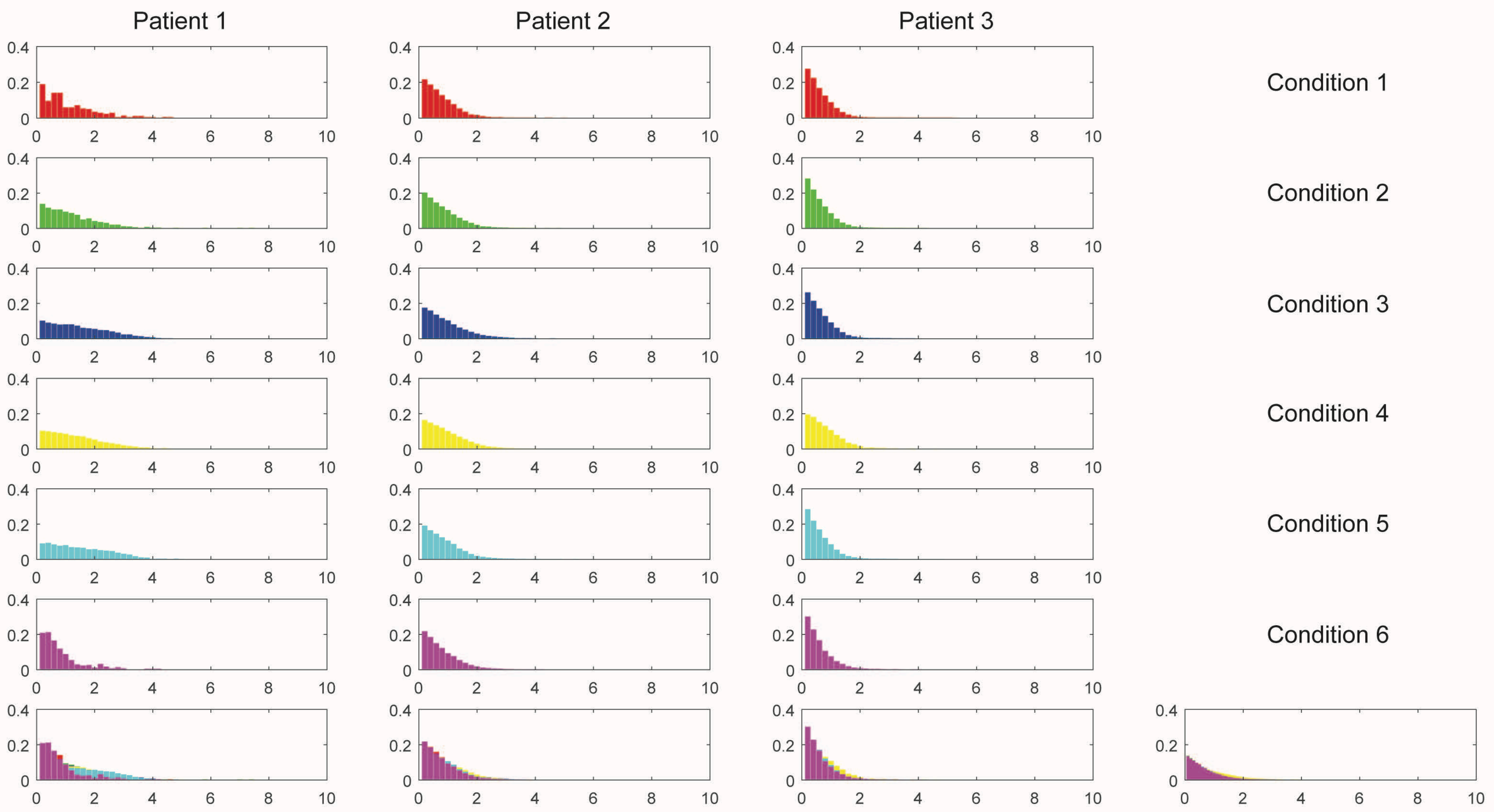

Figure S3.34: Histogram - TAG72

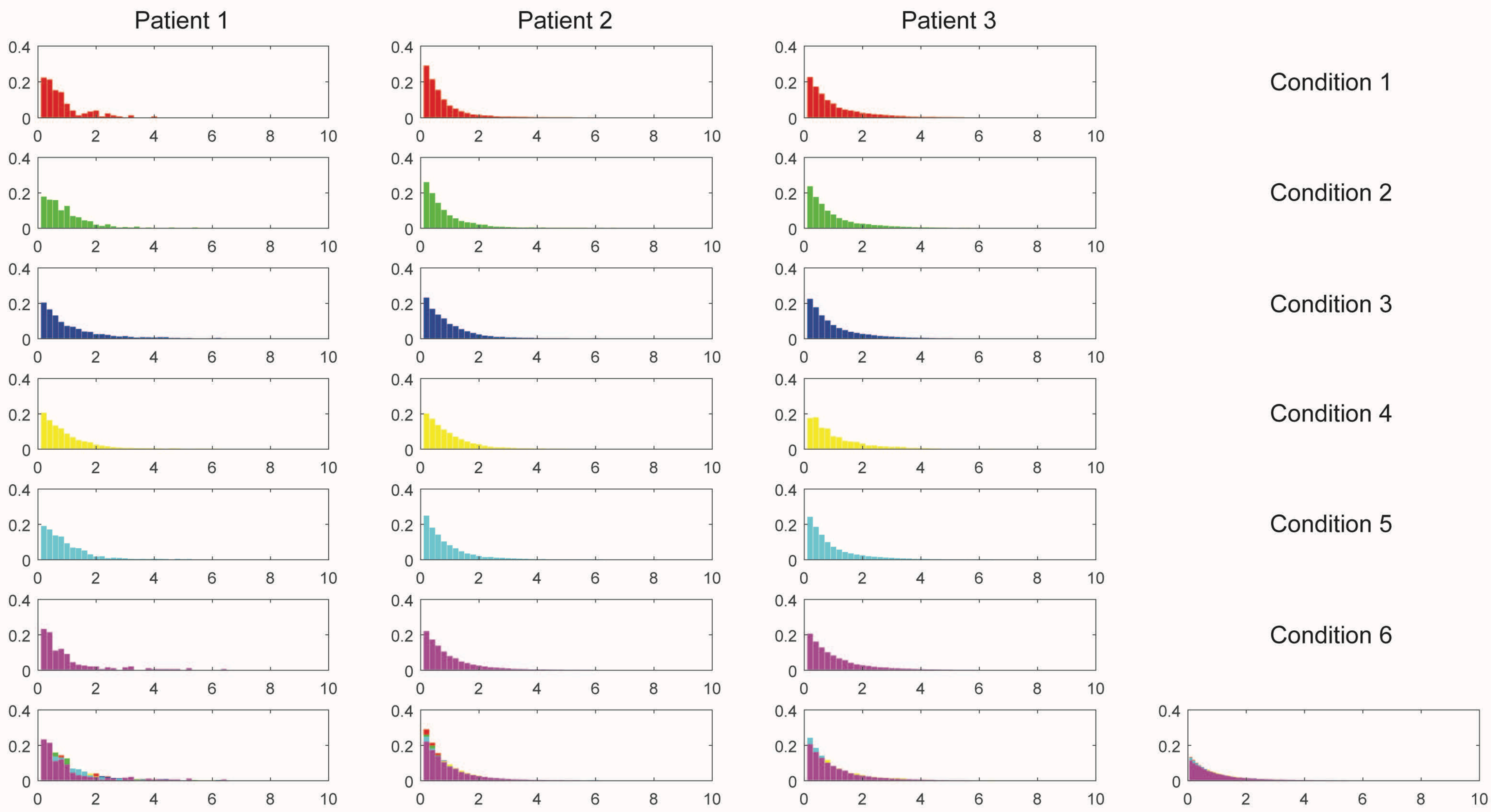

Figure S3.35: Histogram - Tim3

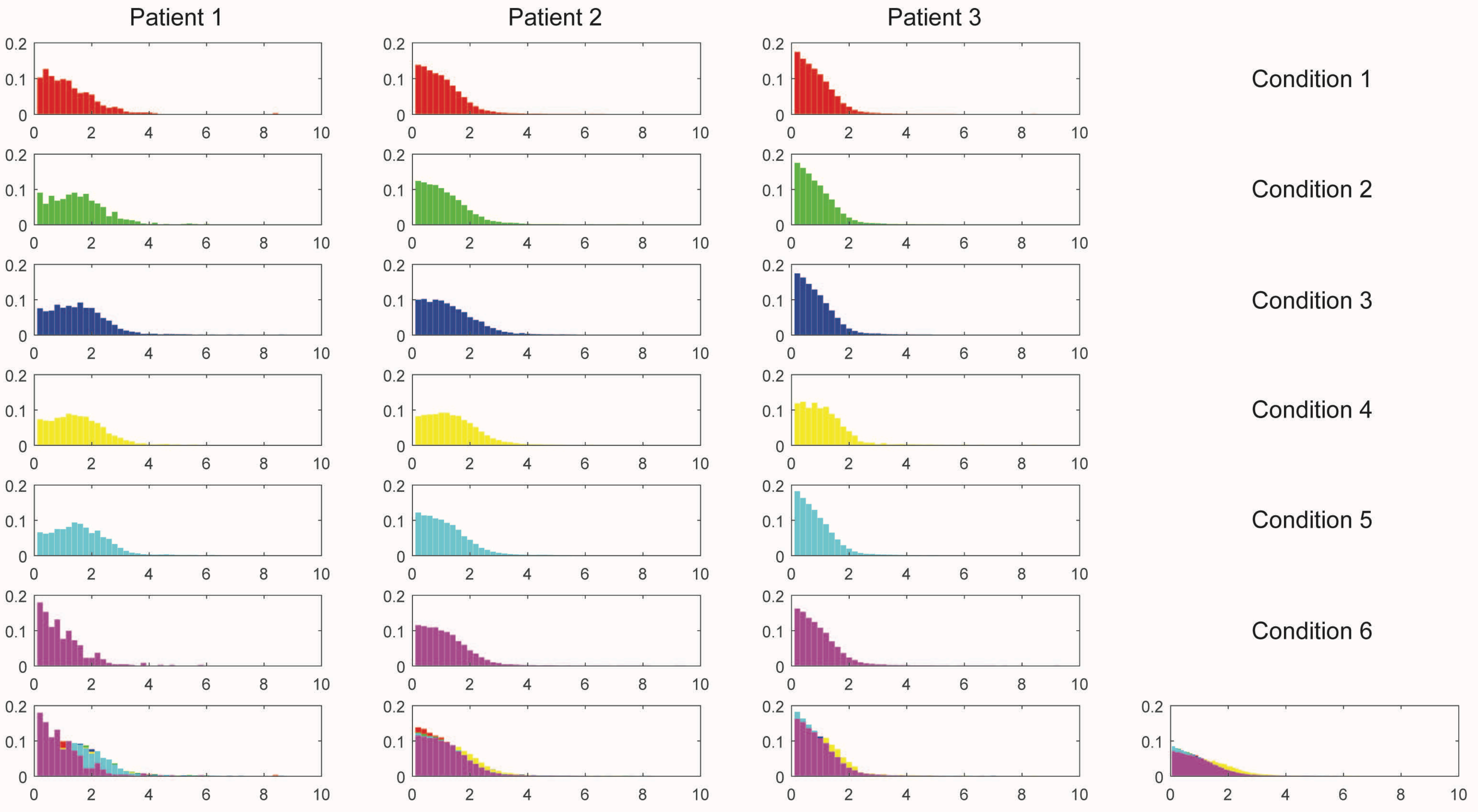

Supplement: Supplementary file 1 [file cancers-13-00755-s001.zip › cancers-1083276 - supplementary/Anandan and Thomsen_2021_Figure S5.pdf]
