# Supplementary material for: Phenotypic Characterization by Mass Cytometry of the Microenvironment in Ovarian Cancer and Impact of Tumor Dissociation Methods
Source: Cancers (Basel). 2021 Feb 11;13(4):755. doi: 10.3390/cancers13040755 (PMC7918057; doi:10.3390/cancers13040755)
Supplement: Supplementary file 1 [file cancers-13-00755-s001.zip › cancers-1083276 - supplementary/cancers-1083276-supplementary_Proof read_10.02.2021.docx]

Supplementary Materials

Phenotypic Characterization by Mass Cytometry of the
Microenvironment in Ovarian Cancer and Impact of Tumor Dissociation Methods

Shamundeeswari Anandan, Liv Cecilie V. Thomsen, Stein-Erik Gullaksen, Tamim Abdelaal, Katrin Kleinmanns, Jørn Skavland, Geir Bredholt, Bjørn Tore Gjertsen, Emmet McCormack and Line Bjørge

**
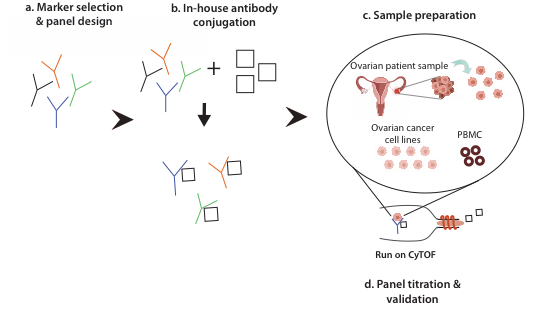
**

**Figure S1.** Overview of ovarian mass cytometry panel development.

**Figure S2.** Antibody staining on unstimulated and PHA-stimulated PBMCs*.* On the left are the markers defined as immune checkpoints in this experiment, the associated metal tags, and the final dilutions selected for further experiments. On the right are the overlaid histograms for each marker in the relevant cell subsets (horizontally) according to dilution (vertically) from the lowest uppermost in the figure to the highest lowermost, alternating between expression demonstrated in unstimulated (Unstim) PBMCs and stimulated (Stim) PBMCs.

EpCAM – tumor marker

Median expression

Median expression

Median expression

FOLR1 – tumor marker

FAPa – stromal marker

**Figure S3.** Stromal and tumor panels were titrated on ovarian cancer cell lines and a mixture of the patient samples. Since the stromal marker fibroblast activation protein alpha (FAPα; left) demonstrated good signals at all dilutions in the patient sample, a lower dilution of 1:800 was selected for this marker. The tumor markers folate receptor (FOLR1; center) and epithelial cell adhesion molecule (EpCAM; right) showed decreasing marker expression with higher dilutions. Still, as the antibodies were expressed at the high dilution (1:1600), this was selected for these markers.

**Figure S4.** Panel validation on pooled patient data. The antibody panel was applied to a pool of dissociated patient tumors (*n* = 4, each dissociated by a different method) with the antibodies diluted according to the results from the previous titration steps. The generated CyTOF data were analyzed in Cytobank, and a heatmap was generated. The main cell subsets (vertically) were defined according to cellular antibody expression (horizontally). The major immune phenotypes (CD45+ cells) were identified, including CD8+cytotoxic T cells, CD4+ T helper cells, CD20+ B cells, and HLA DR+ antigen-presenting cells. Stromal and tumor cells were also identified.

**Figure S5.** Histograms illustrating antibody expression according to three separate patient samples*.* Marker distribution per patient is displayed for all conditions. Probability density function of each marker show distribution differences across patients for all conditions. The x-axis represents marker expression values arcsin-transformed using a cofactor of 5. The marker expression range is limited to between 0.1 and 6 (ignoring the zero peak for visualization purposes). The y-axis represents the probability density, which is the occurrence frequency for different marker expression values. (This is enclosed as a separate file due to size.)

**Figure S6.** Cox proportional-hazard models of median expression of the functional markers. (This is enclosed as a separate file due to size.)

**Table S1:** Overview of antibodies and metal conjugates (Tags) included in the HGSOC TME-based mass cytometry by time-of-flight (CyTOF) panel, the positive and negative controls used for each antibody, and the final antibody dilutions that should be applied to tissues

| Markers | Clone | Tag | Positive control | Negative control | Dilution |
| --- | --- | --- | --- | --- | --- |
| CD8a | RPAT8 | 146Nd | PBMC | OV90*luc+* and CAOV3*luc+* | 1:400 |
| CD4 | RPAT4 | 145Nd | PBMC | OV90*luc+* and CAOV3*luc+* | 1:100 |
| CD3 | UCHT1 | 170Er | PBMC | OV90*luc+* and CAOV3*luc+* | 1:6400 |
| CD45RO | UCHL1 | 164Dy | PBMC | OV90*luc+* and CAOV3*luc+* | 1:200 |
| HLA-DR | L243 | 174Yb | PBMC | OV90*luc+* and CAOV3*luc+* | 1:6400 |
| CD20 | 2H7 | 147Sm | PBMC | OV90*luc+* and CAOV3*luc+* | 1:50 |
| CD25 | 2A3 | 167Er | PBMC | OV90*luc+* and CAOV3*luc+* | 1:50 |
| CD56 | NCAM16.2 | 163Dy | PBMC | OV90*luc+* and CAOV3*luc+* | 1:12800 |
| CD19 | HIB19 | 169Tm | PBMC | OV90*luc+* and CAOV3*luc+* | 1:200 |
| CD14 | M5E2 | 160Gd | PBMC | OV90*luc+* and CAOV3*luc+* | 1:200 |
| CD11b | ICRF44 | 209Bi | PBMC | OV90*luc+* and CAOV3*luc+* | 1:400 |
| CD45 | HI30 | 89Y | PBMC | OV90*luc+* and CAOV3*luc+* | 1:1600 |
| CD103 | BER-ACT8 | 151Eu | PBMC | OV90*luc+* and CAOV3*luc+* | 1:200 |
| CD47* | CC2C6 | 153Eu | PBMC | OV90*luc+* and CAOV3*luc+* | 1:12800 |
| PD1/CD279 | EH12. 2H7 | 155Gd | Stimulated PBMC | PBMC | 1:100 |
| PD-L1/CD274 | 29E.2A3 | 159Tb | Stimulated PBMC | PBMC | 1:200 |
| CTLA-4/CD152 | 14D3 | 161Dy | Stimulated PBMC | PBMC | 1:200 |
| TIM-3 | F38-2E2 | 154Sm | Stimulated PBMC | PBMC | 1:50 |
| LAG-3/CD223 | 11C3C65 | 150Nd | Stimulated PBMC | PBMC | 1:50 |
| OX40/CD134* | Ber-ACT35 (ACT35) | 165Ho | Stimulated PBMC | PBMC | 1:100 |
| Foxp3 | 259D/C7 | 162Dy | Stimulated PBMC | PBMC | 1:800 |
| IFNg | B27 | 168Er | OV90*luc+* and CAOV3*luc+* | PBMC | 1:50 |
| CD34 | 581 | 148Nd | OV90*luc+* and CAOV3*luc+* | PBMC | 1:3200 |
| CD73* | AD2 | 172Yb | OV90*luc+* and CAOV3*luc+* | PBMC | 1:1600 |
| CD24 | ML5 | 166Er | OV90*luc+* and CAOV3*luc+* | PBMC | 1:200 |
| CD44* | BJ18 | 152Sm | OV90*luc+* and CAOV3*luc+* | PBMC | 1:400 |
| CD133* | 5-E3 (5E3) | 142Nd | OV90*luc+* and CAOV3*luc+* | PBMC | 1:200 |
| CD117 | 104D2 | 143Nd | OV90*luc+* and CAOV3*luc+* | PBMC | 1:50 |
| EpCAM/CD326 | 9C4 | 141Pr | OV90*luc+* and CAOV3*luc+* | PBMC | 1:6400 |
| TAG72* | 0.N.561 | 149Sm | OV90*luc+* and CAOV3*luc+* | PBMC | 1:50 |
| FOLR1* | 548908 | 158Gd | OV90*luc+* and CAOV3*luc+* | PBMC | 1:6400 |
| PDGFRB/CD140b | 18A2 | 156Gd | OV90*luc+* and CAOV3*luc+* | PBMC | 1:50 |
| FAPalpha* | F11-24 | 176Yb | OV90*luc+* and CAOV3*luc+* | PBMC | 1:800 |
| aSMA* | 1A4 | 175Lu | OV90*luc+* and CAOV3*luc+* | PBMC | 1:6400 |
| AXL* | MM0098-2N33 | 171Yb | OV90*luc+* and CAOV3*luc+* | PBMC | 1:200 |
| *In-house conjugated antibodies | | | | | |

**Table S2** Antibody-expression of the cell clusters significantly affected by the different dissociation methods

| **Cluster name** | **Antigen expression** |
| --- | --- |
| Immune cluster 7 | CD45+HLA-DR+CD14+ |
| Stromal cluster 5 | EpCAM+CD47+PDGFR+FOLR1+CD56+CD24+ |
| Stromal cluster 6 | EpCAM+CD47+CD56+ |
| Tumor cluster 2 | EpCAM+CD47+FOLR1+ |
| Tumor cluster 4 | EpCAM+CD47+ |
| Tumor cluster 5 - Cancer stem cells | CD34+ |

**Table S3** Overview of the patient cohort (*n* = 3).

| **Patient** | **Age** | **Stage** | **Morphology** | **Chemonaïve** | **Progression-free survival (days)** | **Status** |
| --- | --- | --- | --- | --- | --- | --- |
| 1 | 71 | IIIc | High-grade serous ovarian adenocarcinoma | No | 1516 | Alive without disease |
| 2 | 61 | IIc | High-grade serous ovarian adenocarcinoma | Yes | 449 | Alive with disease |
| 3 | 69 | IIIc | High-grade serous ovarian adenocarcinoma | Yes | 549 | Alive without disease |

**Table S4.** Viability of the cells in the dissociated tissues. For each patient sample and the six dissociation methods the percentage of dead cells in the sample is listed. The viability was measured directly after dissociation of the tumor before freezing.

| **Patient** | **Collagenase** | **Collagenase + trypsin** | **Miltenyi 1 hr** | **Miltenyi 2 hrs** | **Dispase** | **Mechanical** |
| --- | --- | --- | --- | --- | --- | --- |
| 1 | 5.6 | 6.56 | 5.1 | 5.9 | 4.5 | 11.9 |
| 2 | 15 | 14 | 19 | 16 | 14 | 30 |
| 3 | 3.99 | 8.1 | 9.65 | 24.8 | 8.56 | 35.7 |

**Supplementary Material 1.** Marker selection and panel design

A 35-antibody HGSOC panel focusing on cellular components of the tumor microenvironment was developed using markers selected on the basis of a literature review and designed with the panel designer (Fluidigm, CA). The panel comprised three major categories: tumor, stromal, and immune markers. These included 33 surface antibodies and two intercellular antibodies. The 33 selected surface markers included general immune lineage markers (*n* = 15), ovarian stromal, tumor, and stem cell markers (*n* = 12 ), and immune checkpoint antibodies (*n* = 6). Pre-conjugated antibodies (*n* = 24) were purchased from Fluidigm (Supplementary Table 1).

**Supplementary Material 2.** In-house antibody conjugation to rare metals

Apart from the 24 pre-conjugated antibodies purchased, a total of 11 antibodies were successfully conjugated in house. In-house conjugation of carrier-free antibodies (*n* = 11) to metal-chelated polymers (MaxPAR antibody conjugation kit, Fluidigm) was performed according to the manufacturer’s protocol (PRD002 Version 11). The in-house conjugated metal-labeled antibodies were diluted to 0.5mg/mL in antibody stabilization solution (CANDOR Biosciences, Wangen im Allgäu, Germany) and stored at 4°C until required.

**Supplementary Material 3.** *Ex vivo* stimulation of peripheral blood mononuclear cells

Immune checkpoint antibodies were titrated on stimulated peripheral blood mononuclear cells (PBMCs). Prior to stimulation, a batch of cryopreserved PBMCs (collected from healthy donors, as mentioned above) were thawed, slow diluted (1:12) in RPMI 1640 media at room temperature, and pelleted at 300 g for 5 minutes. The cells (3 × 10^6^ cells/mL) were then resuspended in complete RPMI 1640 medium supplemented with 10% FCS, 2 mM L-glutamine, penicillin 100 IU/mL, and 100U/mL Interleukin-2 (IL-2) (Gibco, Thermo Fischer Scientific) and incubated for an hour at 37 °C in a humidified atmosphere with 5% CO2, prior to stimulation with 2.5 μg/mL phytohemagglutinin (PHA, Sigma Aldrich) for 48 hours under the same incubation conditions. After stimulation, the cells were fixed using Stable-Lyse and Stable-Store (Smart Tube Inc., CA) as per the manufacturer’s protocol (Protocol number: SLSSP1TF-150203). Healthy non-stimulated PBMCs used for titrations were fixed following the same protocol.

**Supplementary Material 4.** Panel titration and validation


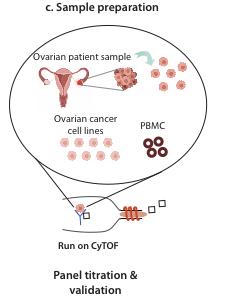


The 35-antibody panel was split into smaller titration panels (immune, stromal/tumor). Two main criteria were included in the design of these titration panels, namely, antibodies with the same metal tags were excluded, and the corresponding M±1 and +16 channels were left open and empty to avoid spillovers and oxide interference.

Initially, a backbone panel with common immune lineage markers was designed, and the rest of the panel was built on this. Immune antibodies were titrated on unstimulated PBMCs and PBMCs stimulated by either phorbol 12-myristate 13-acetate (PMA) (25 ng/mL) and ionomycin (1ug/mL) for 3 or 6 hours or 2.5 μg/mL phytohemagglutinin (PHA) and 100 IU/ml Human Interleukin-2 (IL-2) Recombinant Protein. The immune checkpoint antibodies were titrated successfully when applied to unstimulated and stimulated PBMCs (Figure S2).

**Supplementary Material 5.** Expression of functional markers

The median expression of each of the functional markers included in the panel was evaluated using Cox proportional-hazard models to investigate whether expression levels within the cell populations differed significantly when the six different dissociation methods were applied to the same tissues (Supplementary Figure S6).
