# Supplementary material for: Phenotypic Characterization by Mass Cytometry of the Microenvironment in Ovarian Cancer and Impact of Tumor Dissociation Methods
Source: Cancers (Basel). 2021 Feb 11;13(4):755. doi: 10.3390/cancers13040755 (PMC7918057; doi:10.3390/cancers13040755)
Supplement: Supplementary file 1 [file cancers-13-00755-s001.zip › cancers-1083276 - supplementary/OvCa_panel_dissociations_Functional markers on each population_Figure S6.pdf]

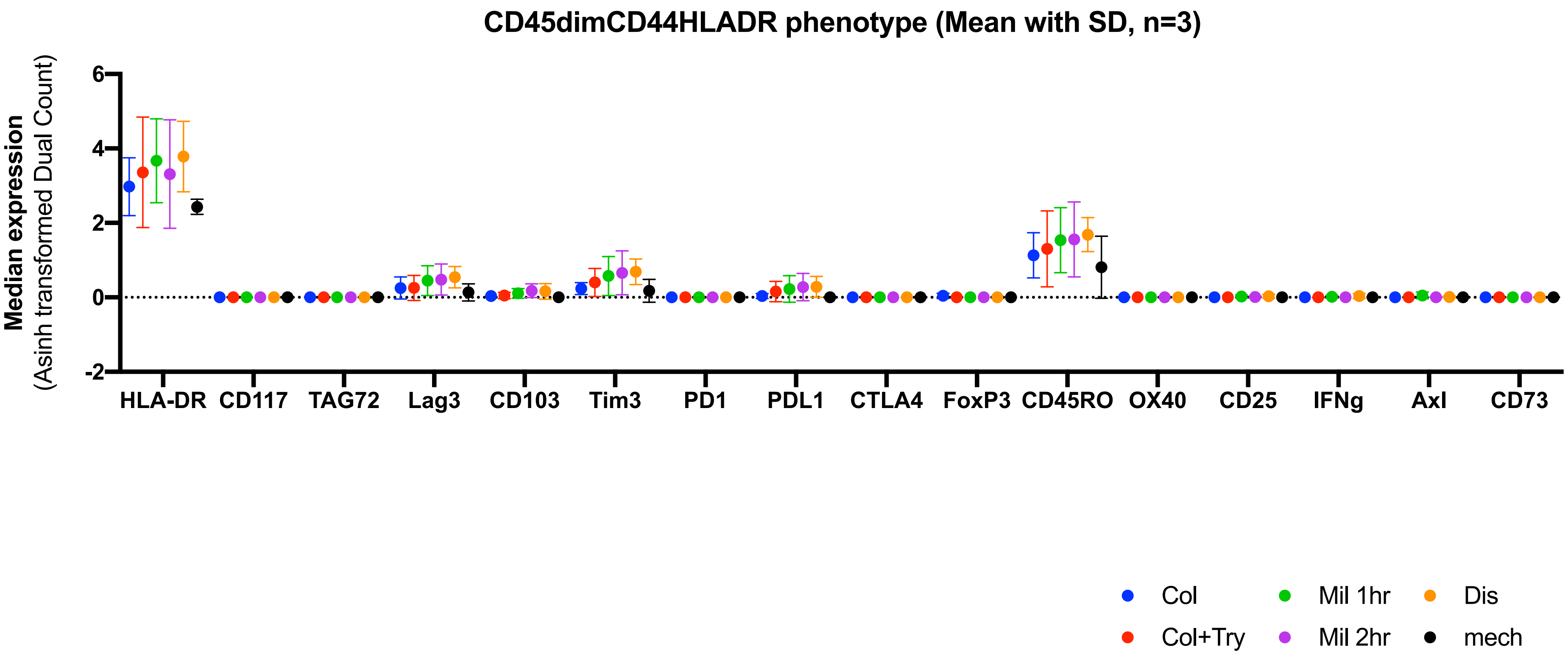
CD45dimCD44HLADR phenotype (Mean with SD, n=3)
Median expression
(Asinh transformed Dual Count)
6
4
2
0
-2
HLA-DR
CD117
TAG72
Lag3
CD103
Tim3
PD1
PDL1
CTLA4
FoxP3
CD45RO
OX40
CD25
IFNg
Axl
CD73
Col
Col+Try
Mil 1hr
Mil 2hr
Dis
mech

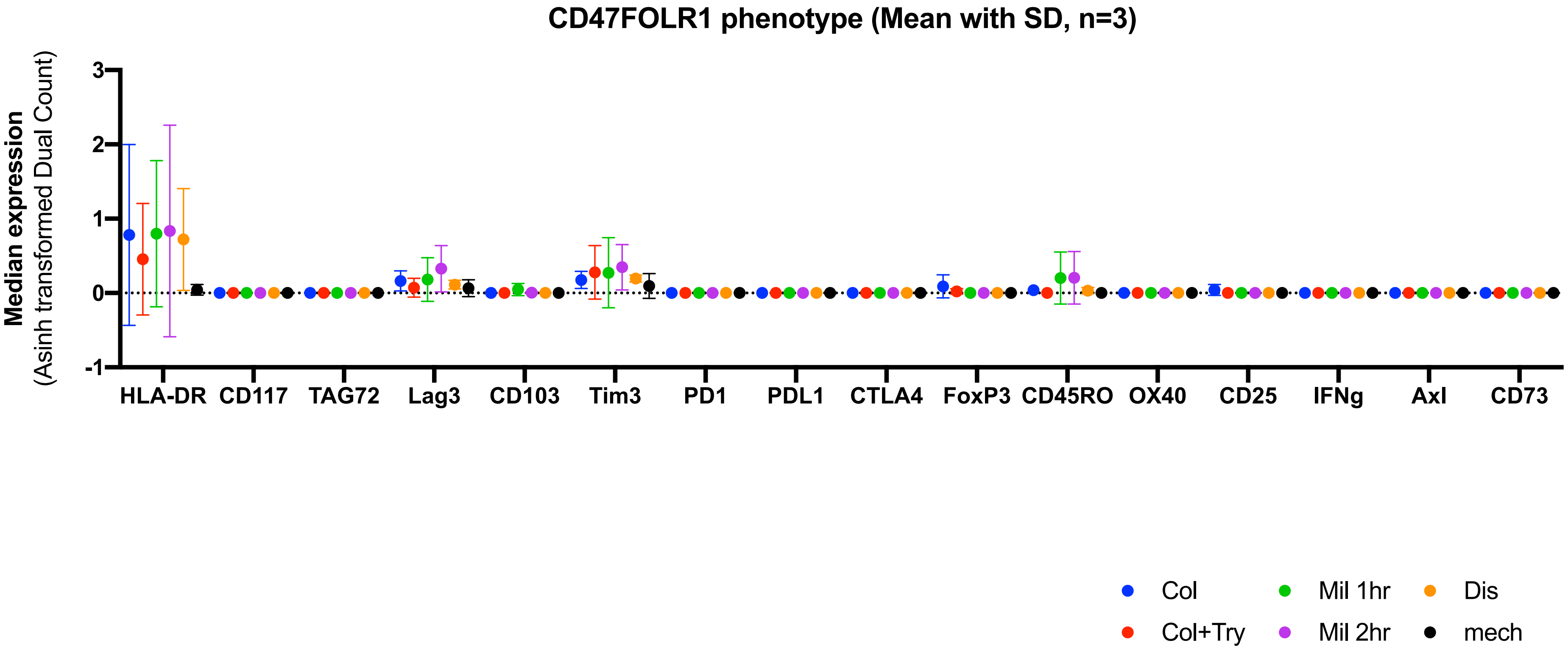
CD47FOLR1 phenotype (Mean with SD, n=3)
Median expression
(Asinh transformed Dual Count)
3
2
1
0
-1
HLA-DR
CD117
TAG72
Lag3
CD103
Tim3
PD1
PDL1
CTLA4
FoxP3
CD45RO
OX40
CD25
IFNg
Axl
CD73
Col
Col+Try
Mil 1hr
Mil 2hr
Dis
mech

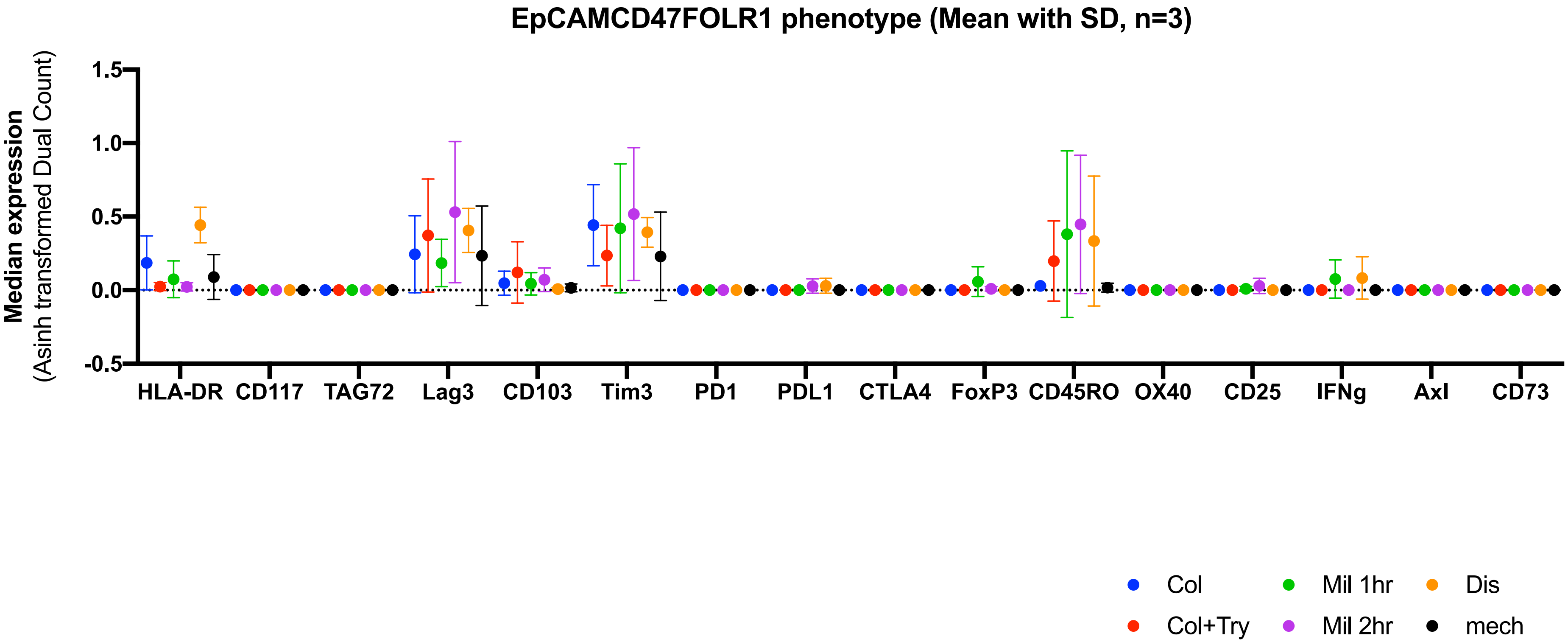

EpCAMCD47FOLR1 phenotype (Mean with SD, n=3)
Median expression
(Asinh transformed Dual Count)
1.5
1.0
0.5
0.0
-0.5
HLA-DR
CD117
TAG72
Lag3
CD103
Tim3
PD1
PDL1
CTLA4
FoxP3
CD45RO
OX40
CD25
IFNg
Axl
CD73
Col
Col+Try
Mil 1hr
Mil 2hr
Dis
mech

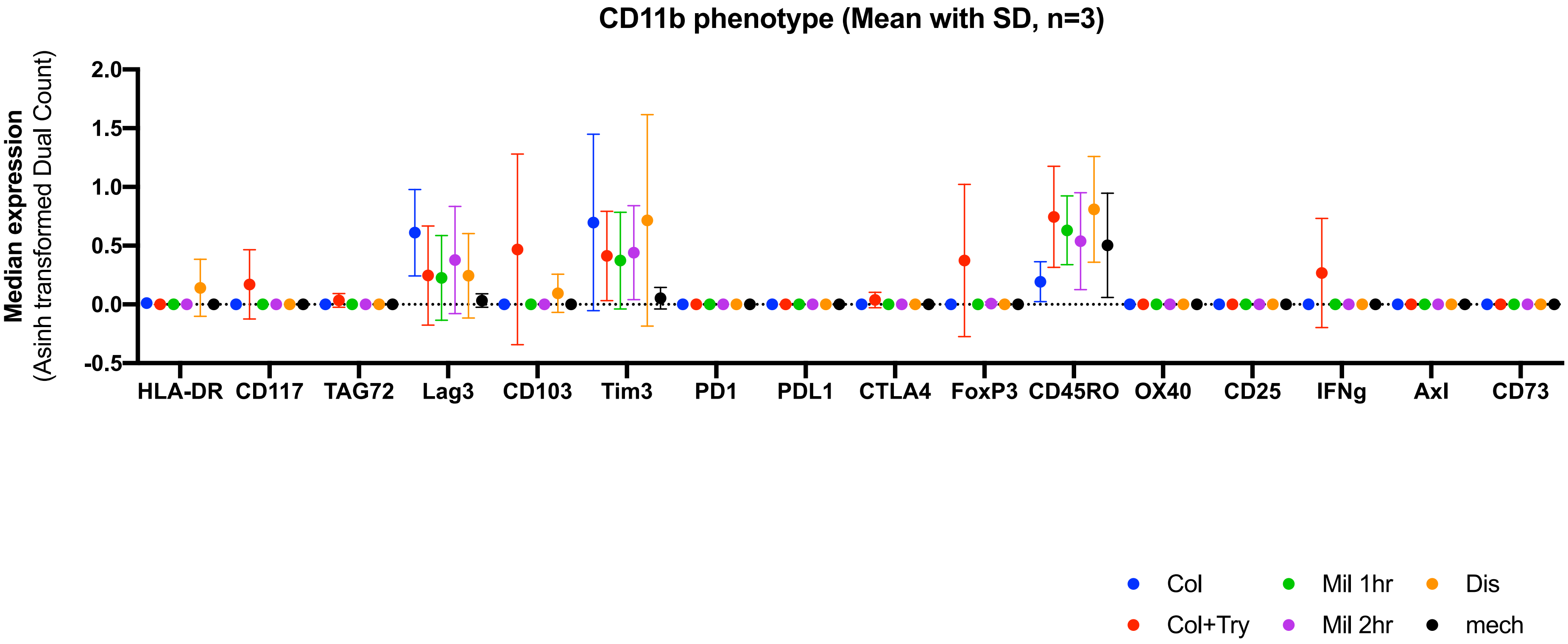
CD11b phenotype (Mean with SD, n=3)
Median expression
(Asinh transformed Dual Count)
2.0
1.5
1.0
0.5
0.0
-0.5
HLA-DR
CD117
TAG72
Lag3
CD103
Tim3
PD1
PDL1
CTLA4
FoxP3
CD45RO
OX40
CD25
IFNg
Axl
CD73
Col
Col+Try
Mil 1hr
Mil 2hr
Dis
mech

CD3 negative!

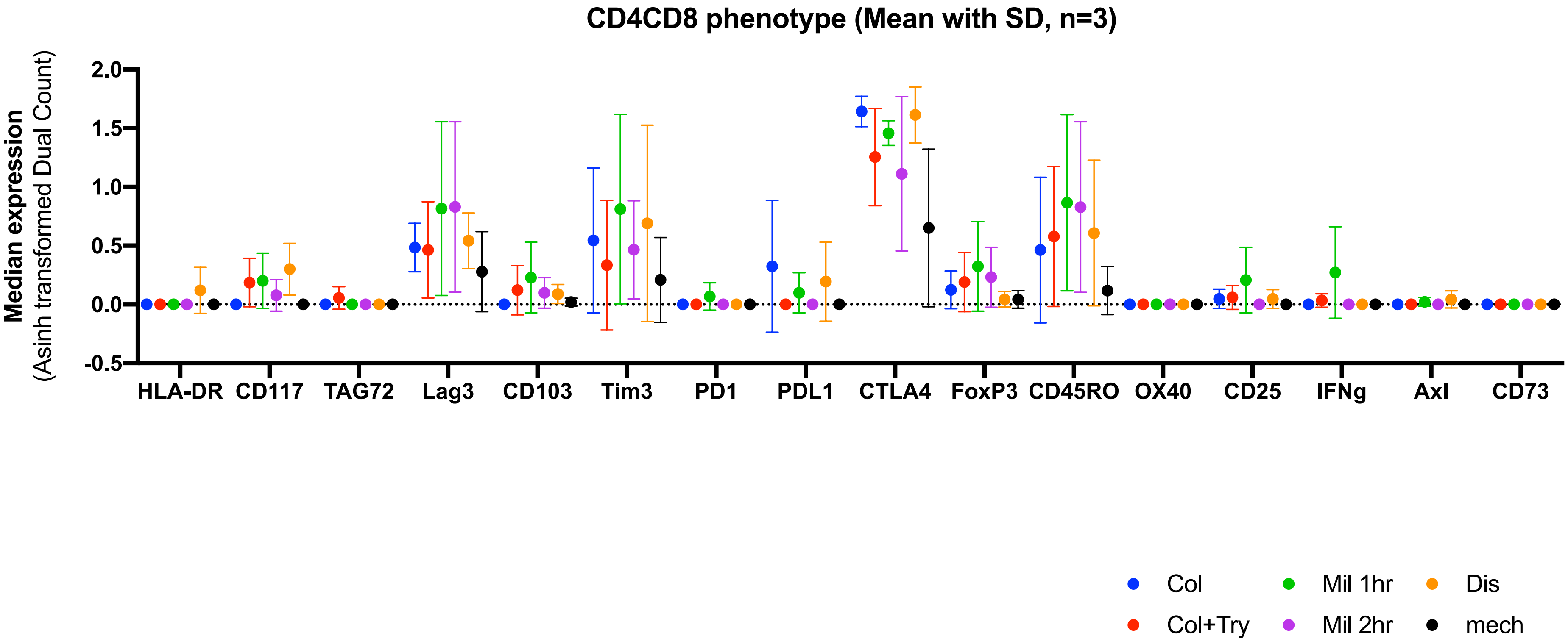

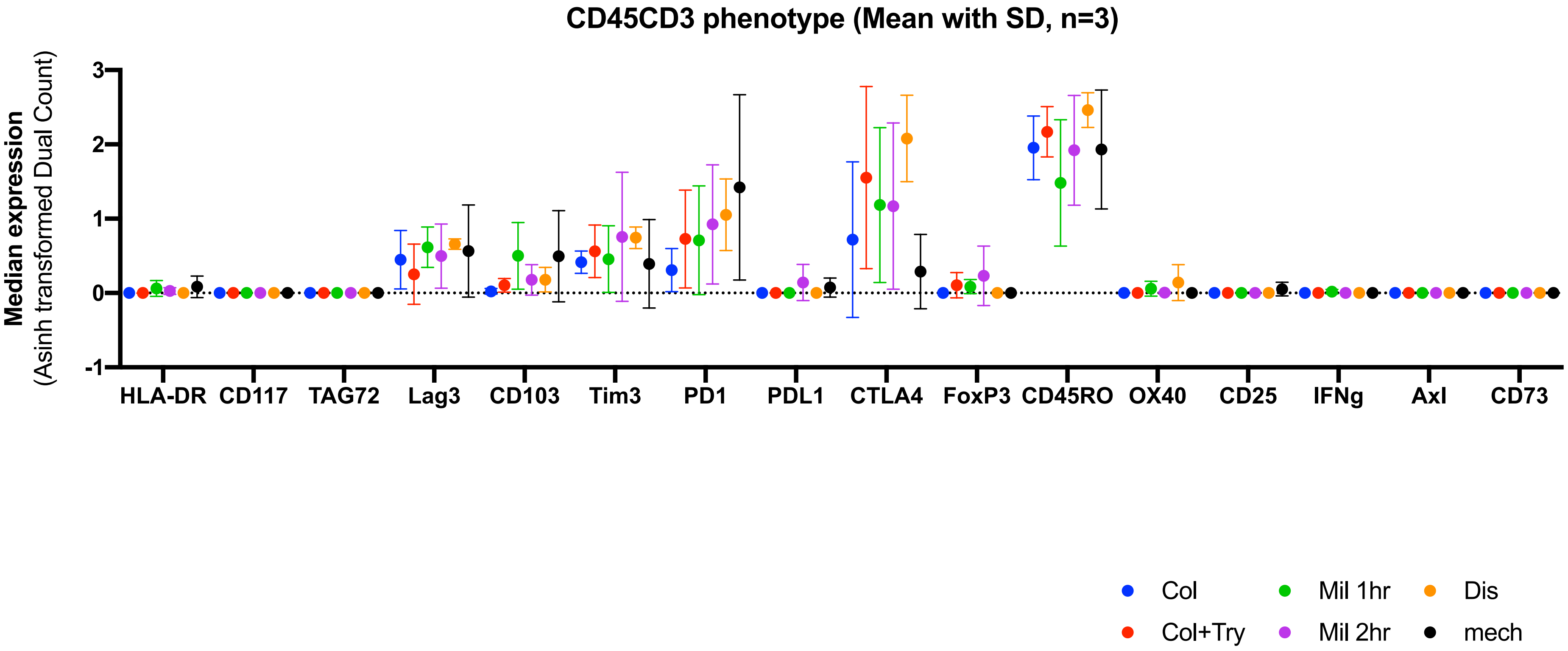
CD45CD3 phenotype (Mean with SD, n=3)
Median expression
(Asinh transformed Dual Count)
3
2
1
0
-1
HLA-DR
CD117
TAG72
Lag3
CD103
Tim3
PD1
PDL1
CTLA4
FoxP3
CD45RO
OX40
CD25
IFNg
Axl
CD73
Col
Col+Try
Mil 1hr
Mil 2hr
Dis
mech

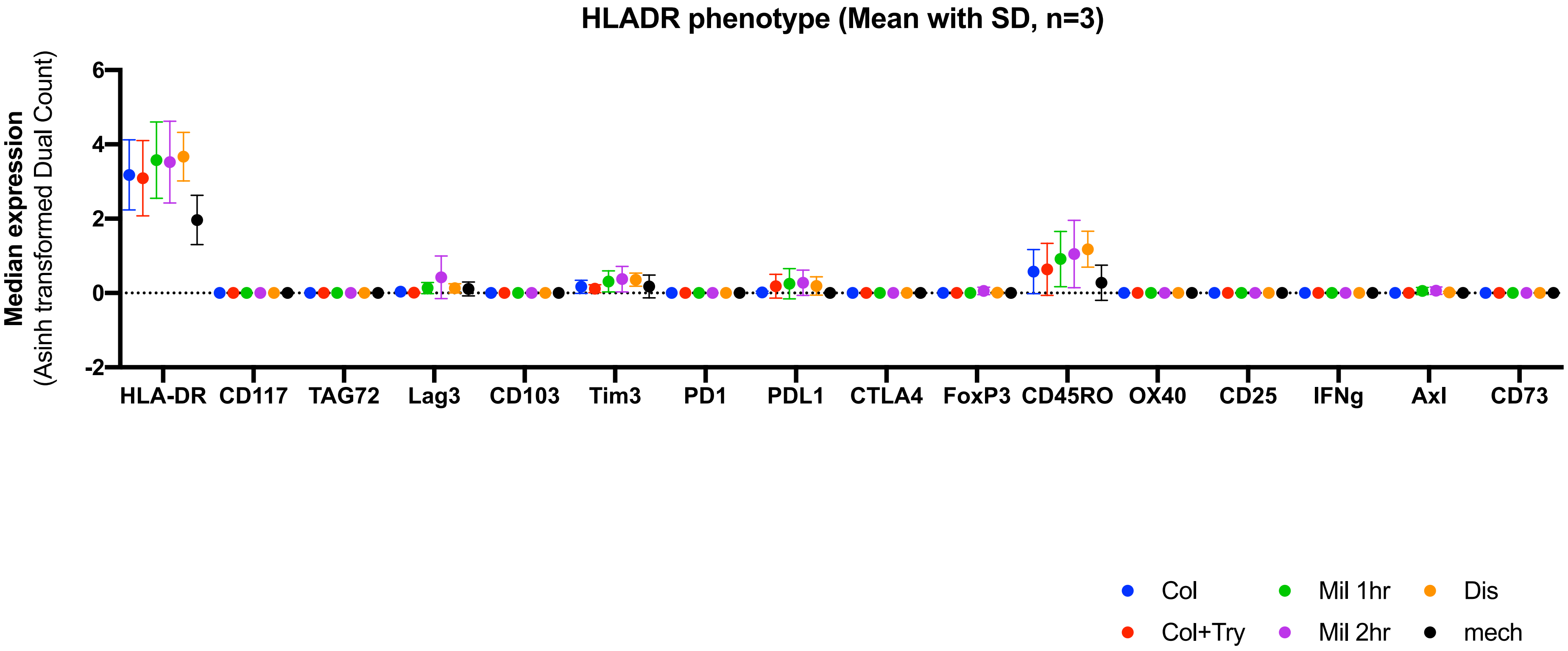

HLADR phenotype (Mean with SD, n=3)
Median expression
(Asinh transformed Dual Count)
6
4
2
0
-2
HLA-DR
CD117
TAG72
Lag3
CD103
Tim3
PD1
PDL1
CTLA4
FoxP3
CD45RO
OX40
CD25
IFNg
Axl
CD73
Col
Col+Try
Mil 1hr
Mil 2hr
Dis
mech

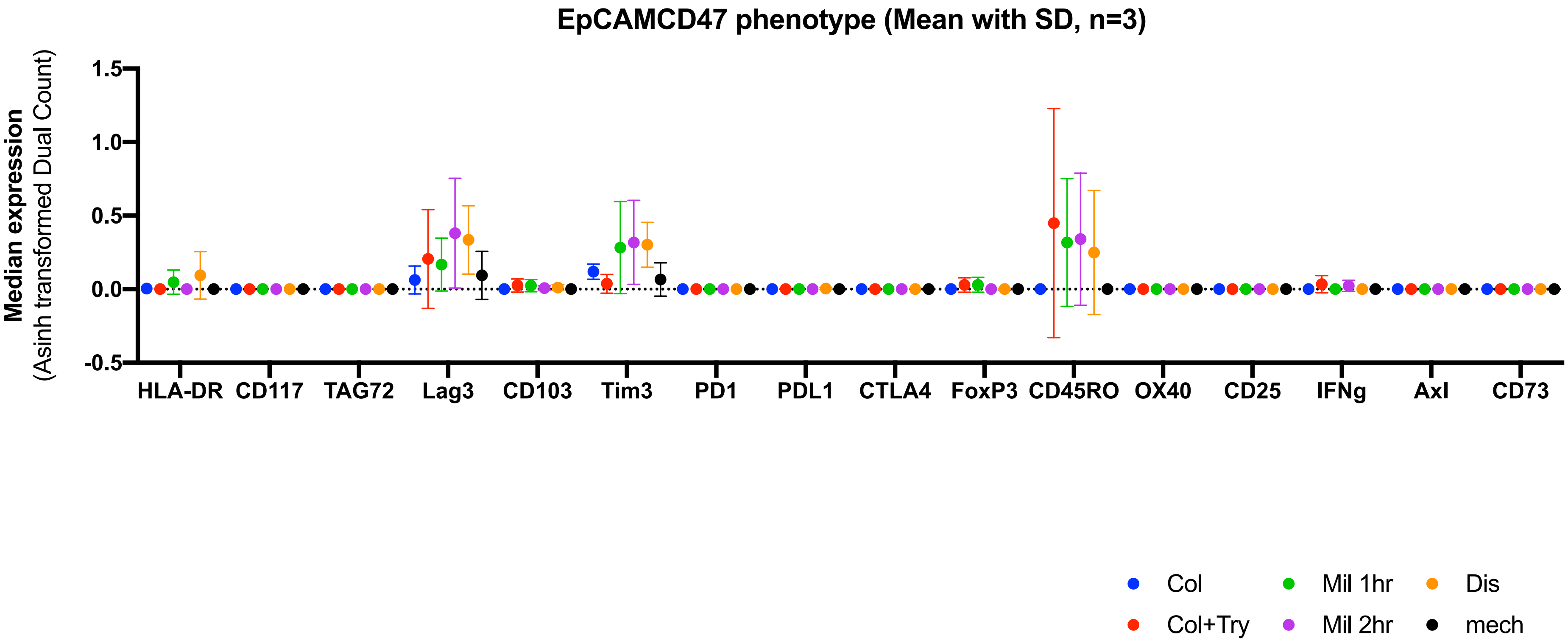
EpCAMCD47 phenotype (Mean with SD, n=3)
Median expression
(Asinh transformed Dual Count)
1.5
1.0
0.5
0.0
-0.5
HLA-DR
CD117
TAG72
Lag3
CD103
Tim3
PD1
PDL1
CTLA4
FoxP3
CD45RO
OX40
CD25
IFNg
Axl
CD73
Col
Col+Try
Mil 1hr
Mil 2hr
Dis
mech

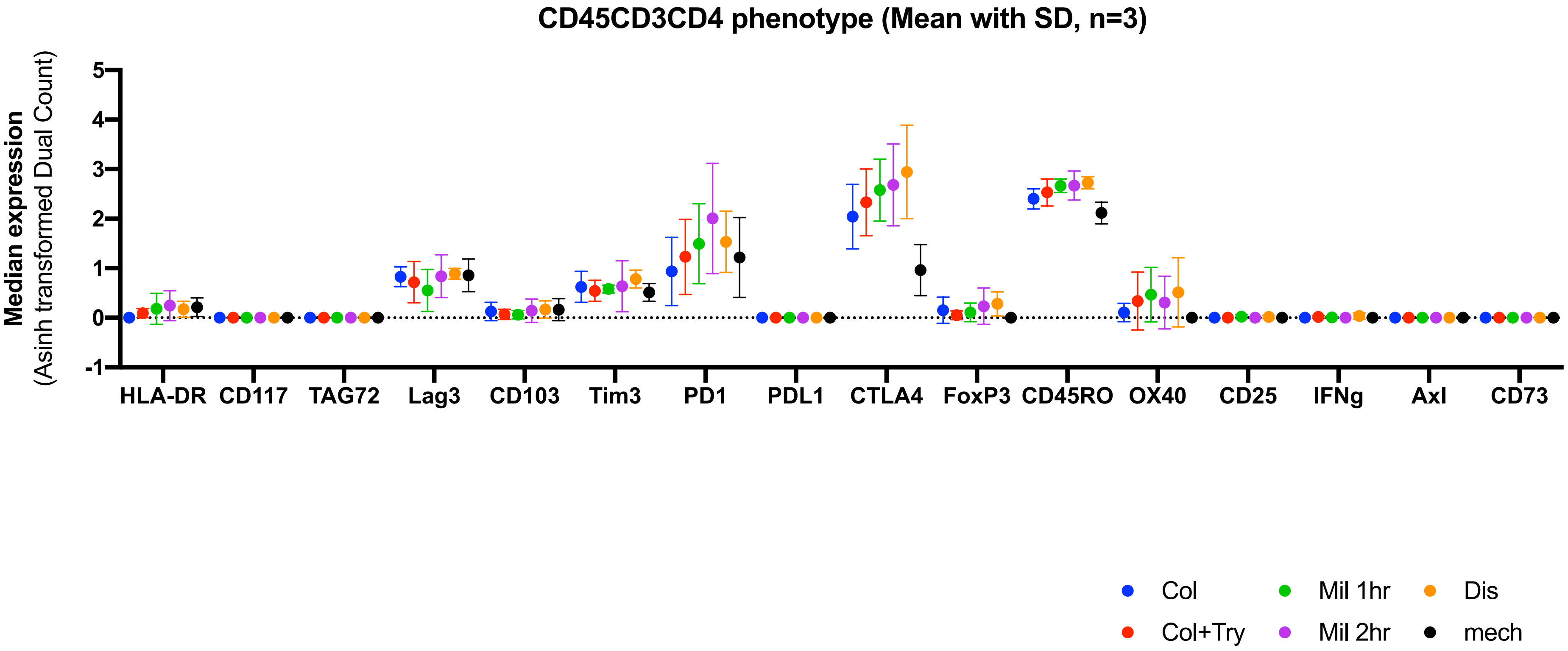
CD45CD3CD4 phenotype (Mean with SD, n=3)
Median expression
(Asinh transformed Dual Count)
5
4
3
2
1
0
-1
HLA-DR
CD117
TAG72
Lag3
CD103
Tim3
PD1
PDL1
CTLA4
FoxP3
CD45RO
OX40
CD25
IFNg
Axl
CD73
Col
Col+Try
Mil 1hr
Mil 2hr
Dis
mech

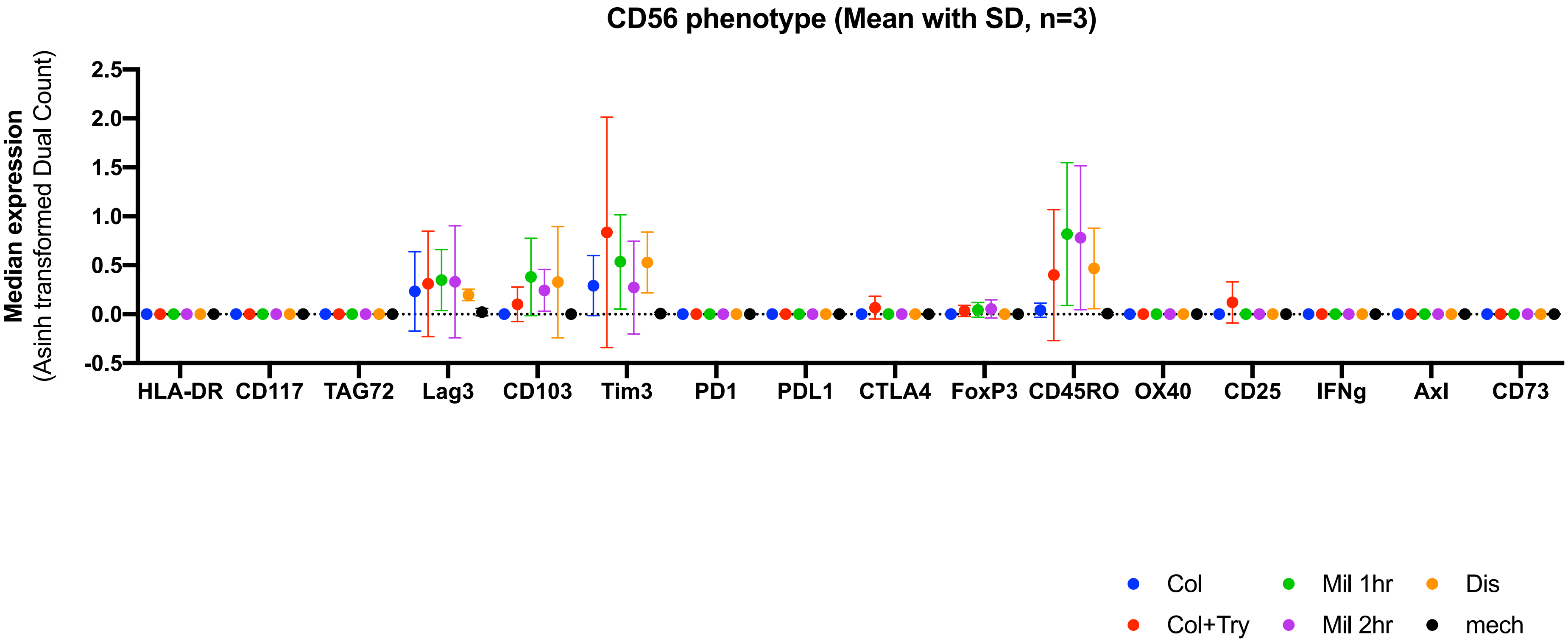

CD56 phenotype (Mean with SD, n=3)
Median expression
(Asinh transformed Dual Count)
2.5
2.0
1.5
1.0
0.5
0.0
-0.5
HLA-DR
CD117
TAG72
Lag3
CD103
Tim3
PD1
PDL1
CTLA4
FoxP3
CD45RO
OX40
CD25
IFNg
Axl
CD73
Col
Col+Try
Mil 1hr
Mil 2hr
Dis
mech

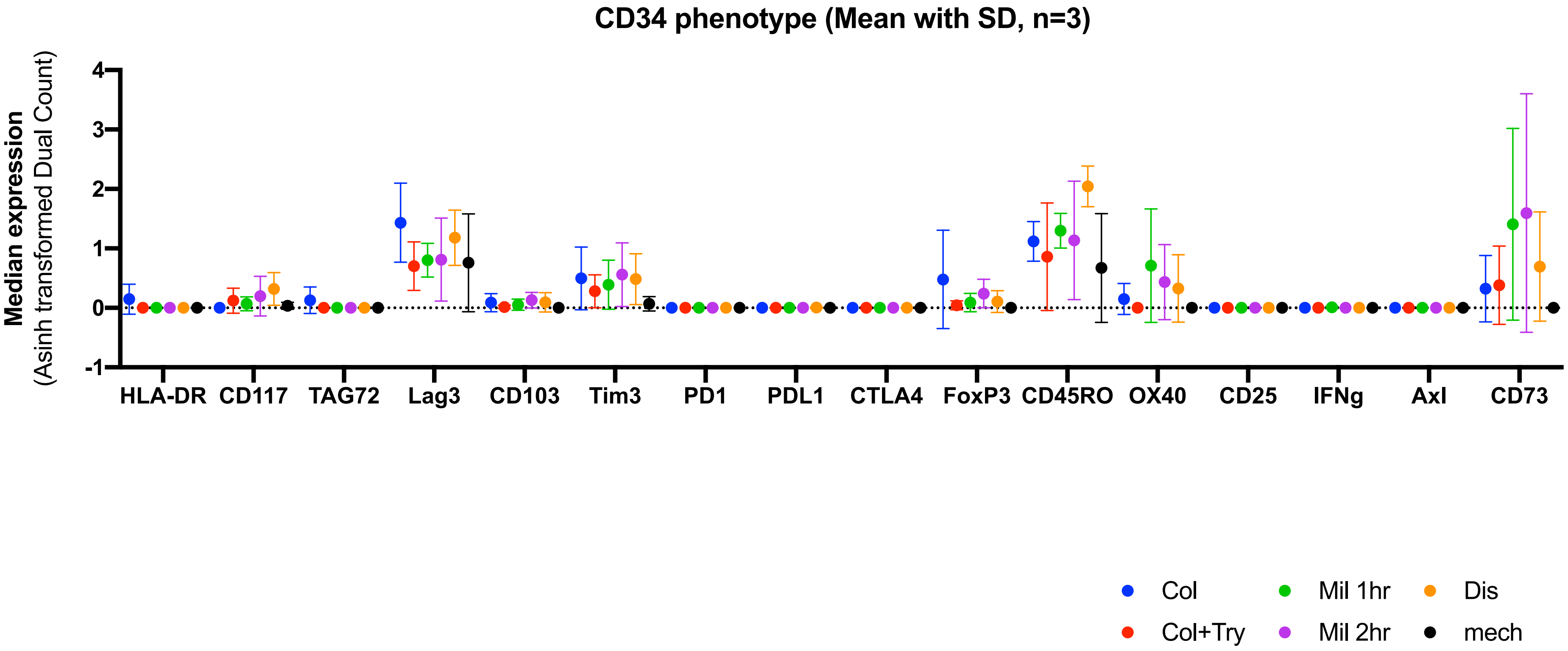

CD34 phenotype (Mean with SD, n=3)
Median expression
(Asinh transformed Dual Count)
4
3
2
1
0
-1
HLA-DR
CD117
TAG72
Lag3
CD103
Tim3
PD1
PDL1
CTLA4
FoxP3
CD45RO
OX40
CD25
IFNg
Axl
CD73
Col
Col+Try
Mil 1hr
Mil 2hr
Dis
mech

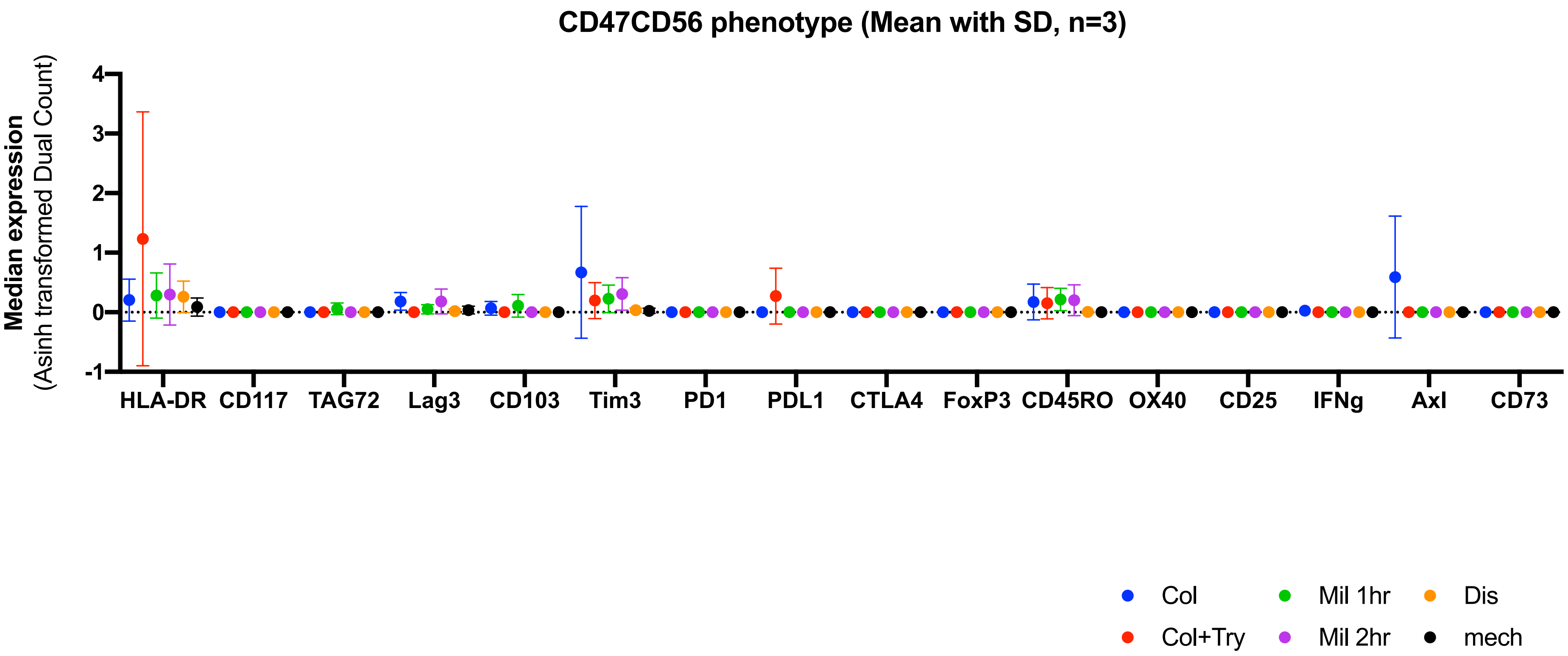
CD47CD56 phenotype (Mean with SD, n=3)
Median expression
(Asinh transformed Dual Count)
4
3
2
1
0
-1
HLA-DR
CD117
TAG72
Lag3
CD103
Tim3
PD1
PDL1
CTLA4
FoxP3
CD45RO
OX40
CD25
IFNg
Axl
CD73
Col
Col+Try
Mil 1hr
Mil 2hr
Dis
mech

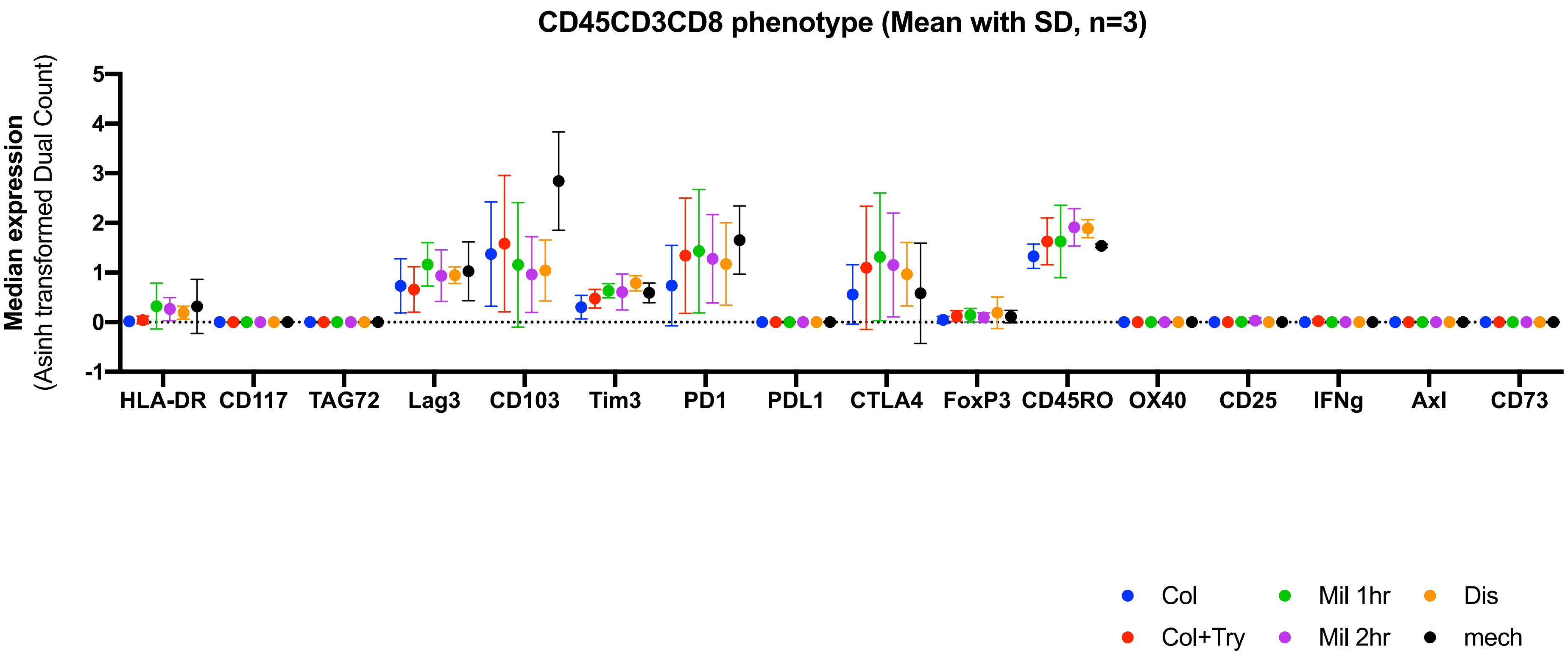

CD45CD3CD8 phenotype (Mean with SD, n=3)
Median expression
(Asinh transformed Dual Count)
5
4
3
2
1
0
-1
HLA-DR
CD117
TAG72
Lag3
CD103
Tim3
PD1
PDL1
CTLA4
FoxP3
CD45RO
OX40
CD25
IFNg
Axl
CD73
Col
Col+Try
Mil 1hr
Mil 2hr
Dis
mech

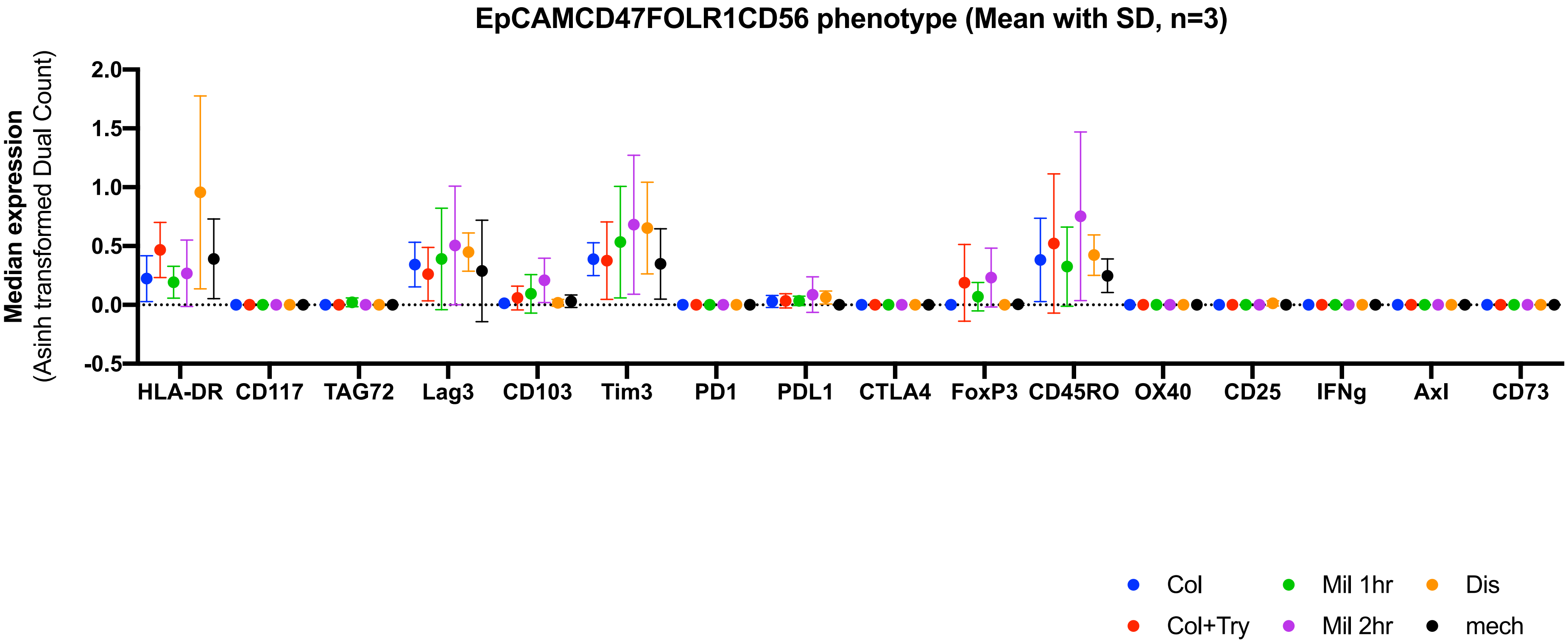
EpCAMCD47FOLR1CD56 phenotype (Mean with SD, n=3)
Median expression
(Asinh transformed Dual Count)
2.0
1.5
1.0
0.5
0.0
-0.5
HLA-DR
CD117
TAG72
Lag3
CD103
Tim3
PD1
PDL1
CTLA4
FoxP3
CD45RO
OX40
CD25
IFNg
Axl
CD73
Col
Col+Try
Mil 1hr
Mil 2hr
Dis
mech

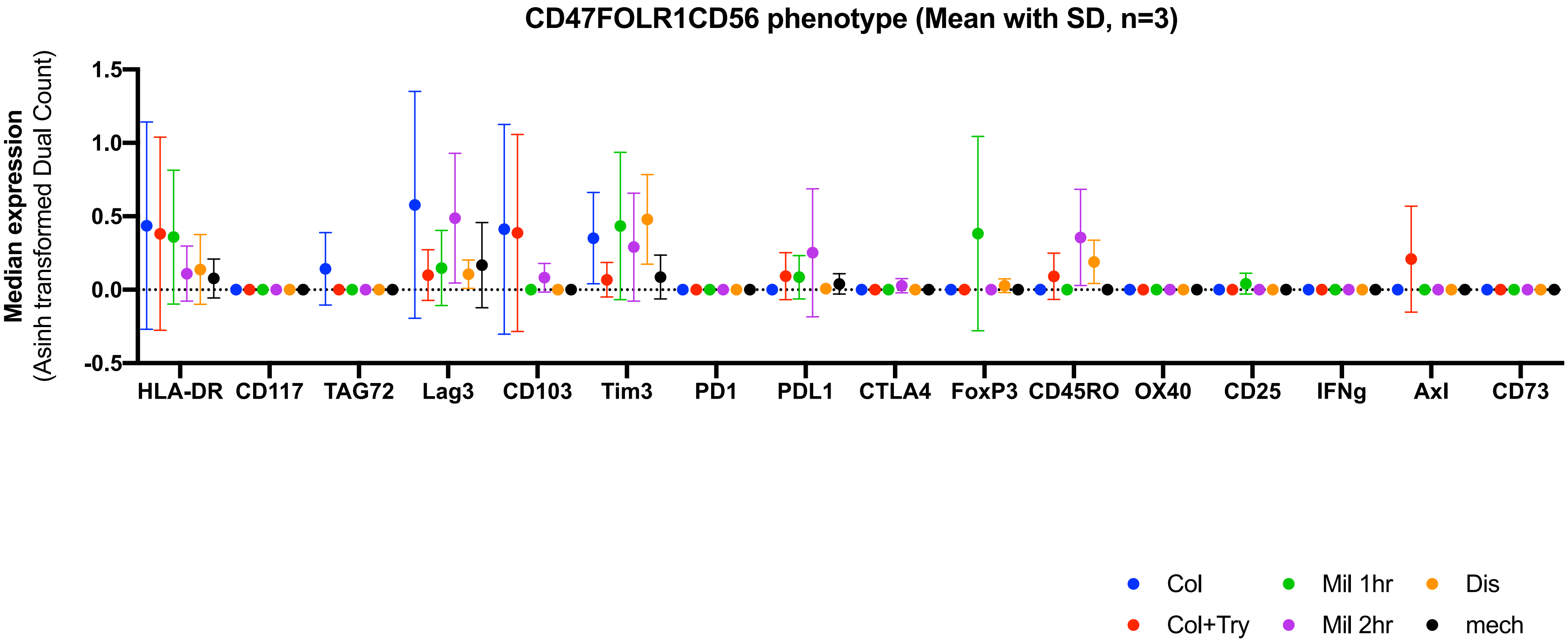
CD47FOLR1CD56 phenotype (Mean with SD, n=3)
Median expression
(Asinh transformed Dual Count)
1.5
1.0
0.5
0.0
-0.5
HLA-DR
CD117
TAG72
Lag3
CD103
Tim3
PD1
PDL1
CTLA4
FoxP3
CD45RO
OX40
CD25
IFNg
Axl
CD73
Col
Col+Try
Mil 1hr
Mil 2hr
Dis
mech

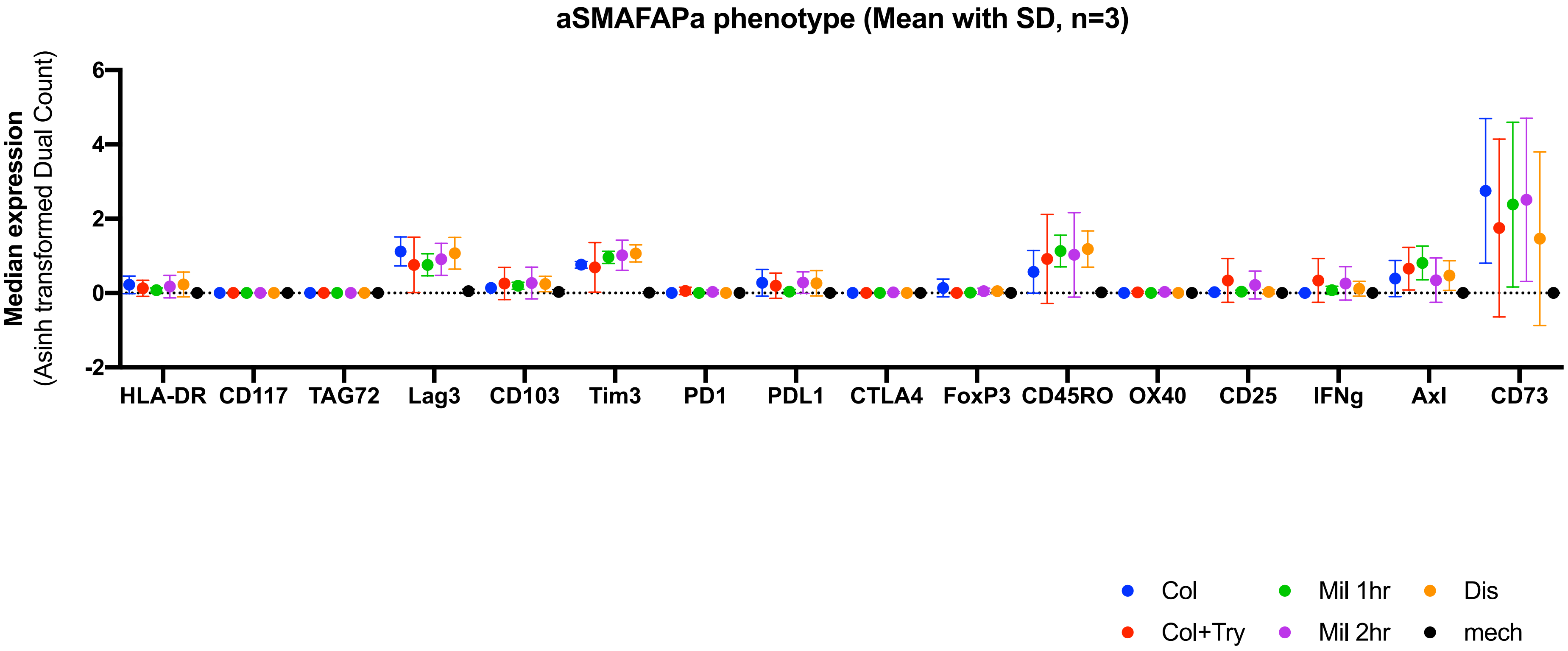
aSMAFAPa phenotype (Mean with SD, n=3)
Median expression
(Asinh transformed Dual Count)
6
4
2
0
-2
HLA-DR
CD117
TAG72
Lag3
CD103
Tim3
PD1
PDL1
CTLA4
FoxP3
CD45RO
OX40
CD25
IFNg
Axl
CD73
Col
Col+Try
Mil 1hr
Mil 2hr
Dis
mech

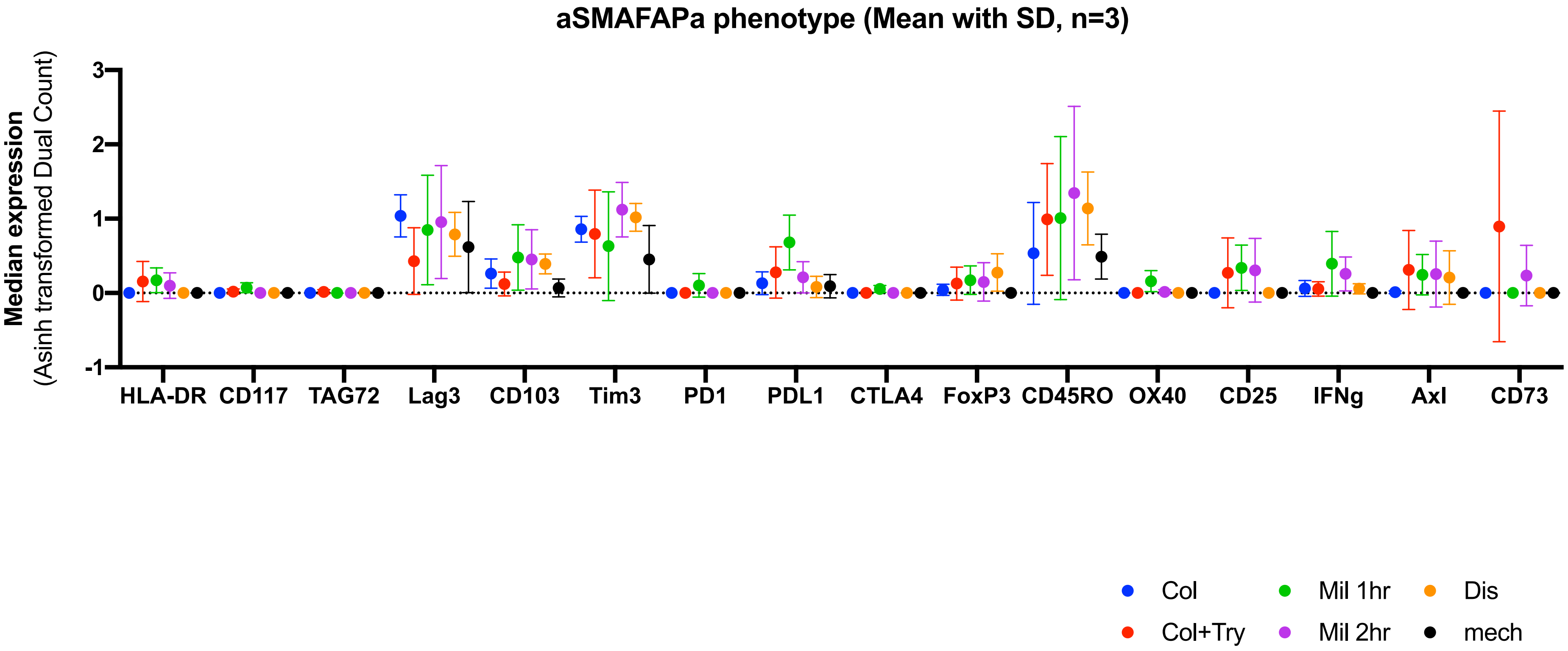
aSMAFAPa phenotype (Mean with SD, n=3)
Median expression
(Asinh transformed Dual Count)
3
2
1
0
-1
HLA-DR
CD117
TAG72
Lag3
CD103
Tim3
PD1
PDL1
CTLA4
FoxP3
CD45RO
OX40
CD25
IFNg
Axl
CD73
Col
Col+Try
Mil 1hr
Mil 2hr
Dis
mech

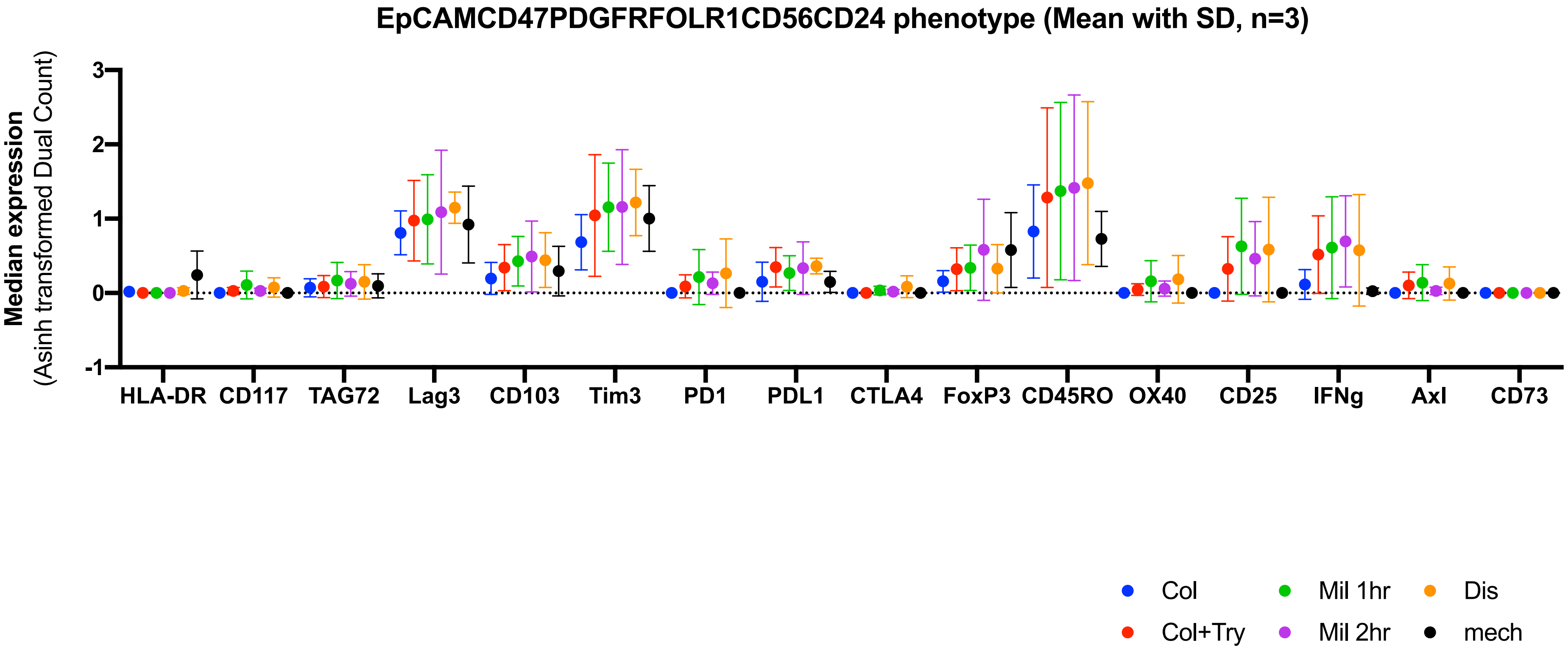
EpCAMCD47PDGFRFOLR1CD56CD24 phenotype (Mean with SD, n=3)
Median expression
(Asinh transformed Dual Count)
3
2
1
0
-1
HLA-DR
CD117
TAG72
Lag3
CD103
Tim3
PD1
PDL1
CTLA4
FoxP3
CD45RO
OX40
CD25
IFNg
Axl
CD73
Col
Col+Try
Mil 1hr
Mil 2hr
Dis
mech

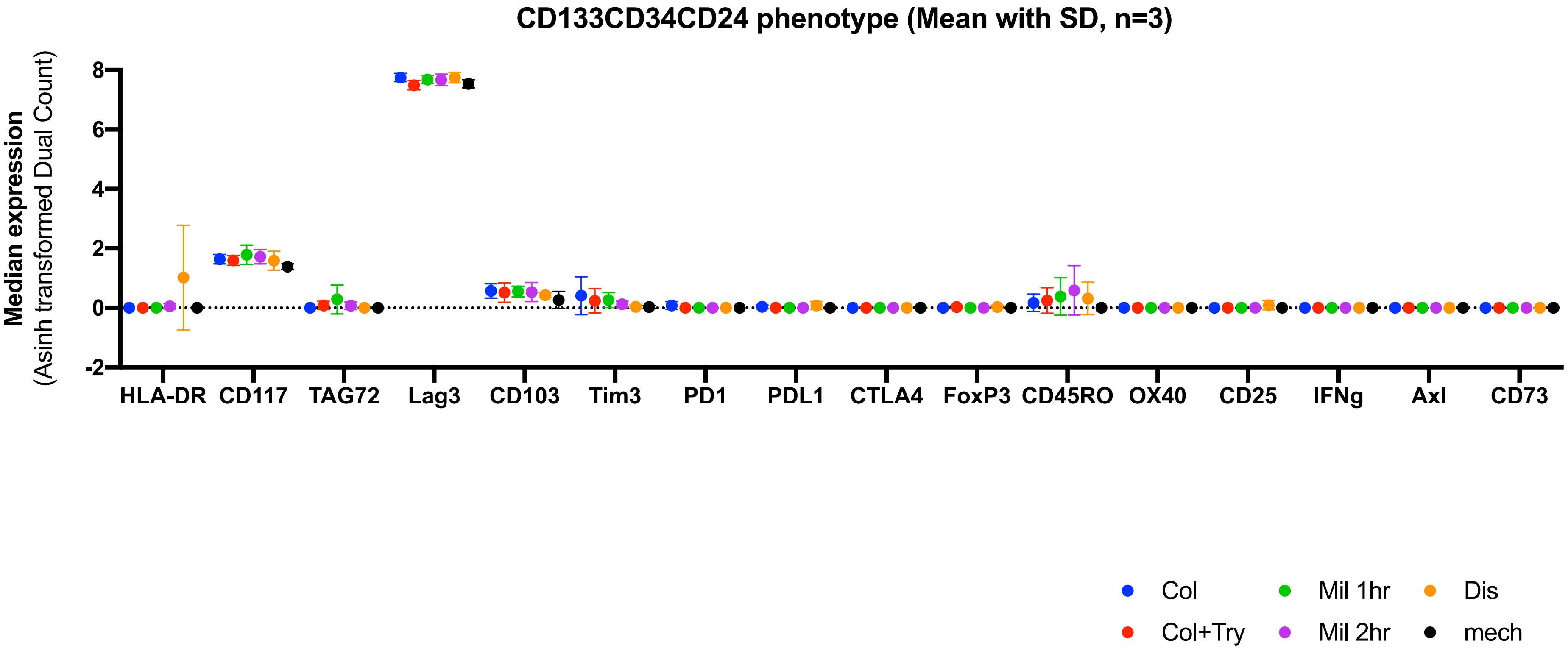
CD133CD34CD24 phenotype (Mean with SD, n=3)
Median expression
(Asinh transformed Dual Count)
8
6
4
2
0
-2
HLA-DR
CD117
TAG72
Lag3
CD103
Tim3
PD1
PDL1
CTLA4
FoxP3
CD45RO
OX40
CD25
IFNg
Axl
CD73
Col
Col+Try
Mil 1hr
Mil 2hr
Dis
mech

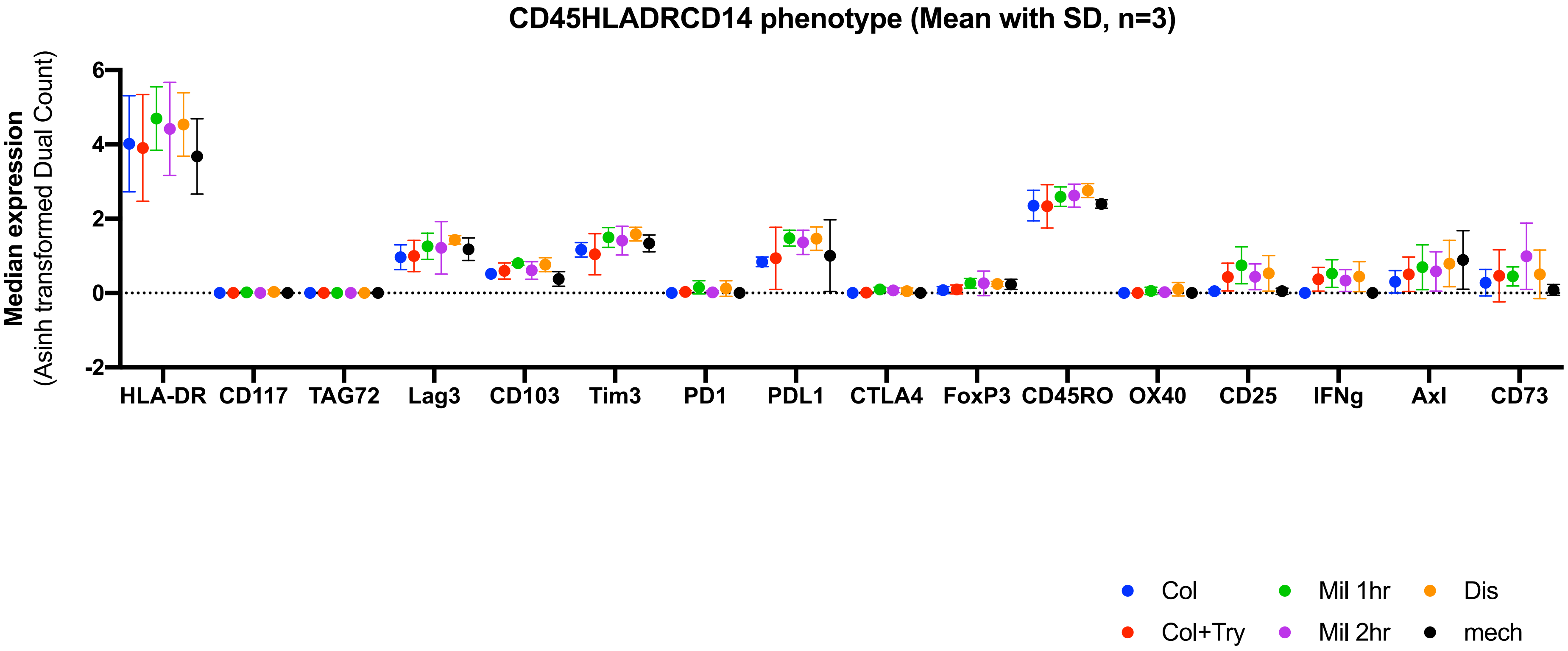
CD45HLADRCD14 phenotype (Mean with SD, n=3)
Median expression
(Asinh transformed Dual Count)
6
4
2
0
-2
HLA-DR
CD117
TAG72
Lag3
CD103
Tim3
PD1
PDL1
CTLA4
FoxP3
CD45RO
OX40
CD25
IFNg
Axl
CD73
Col
Col+Try
Mil 1hr
Mil 2hr
Dis
mech

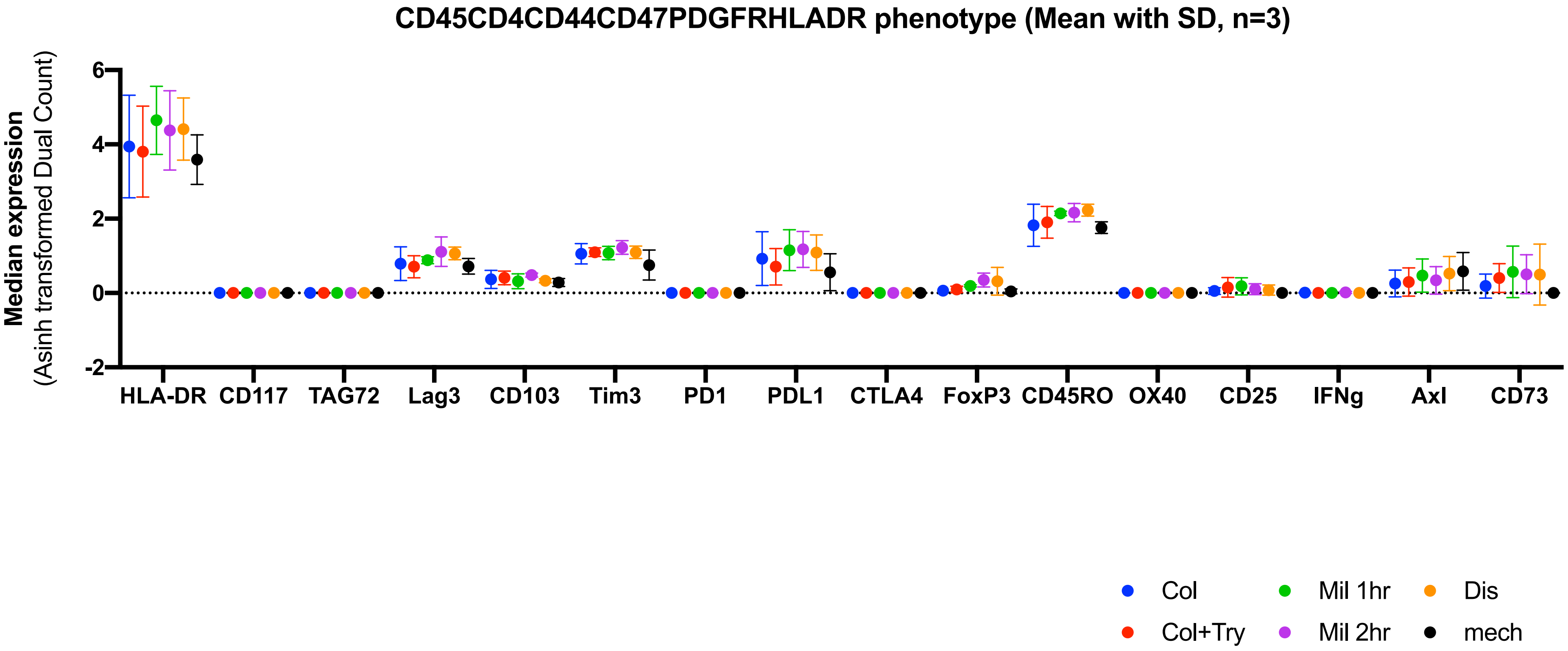
CD45CD4CD44CD47PDGFRHLADR phenotype (Mean with SD, n=3)
Median expression
(Asinh transformed Dual Count)
6
4
2
0
-2
HLA-DR
CD117
TAG72
Lag3
CD103
Tim3
PD1
PDL1
CTLA4
FoxP3
CD45RO
OX40
CD25
IFNg
Axl
CD73
Col
Col+Try
Mil 1hr
Mil 2hr
Dis
mech

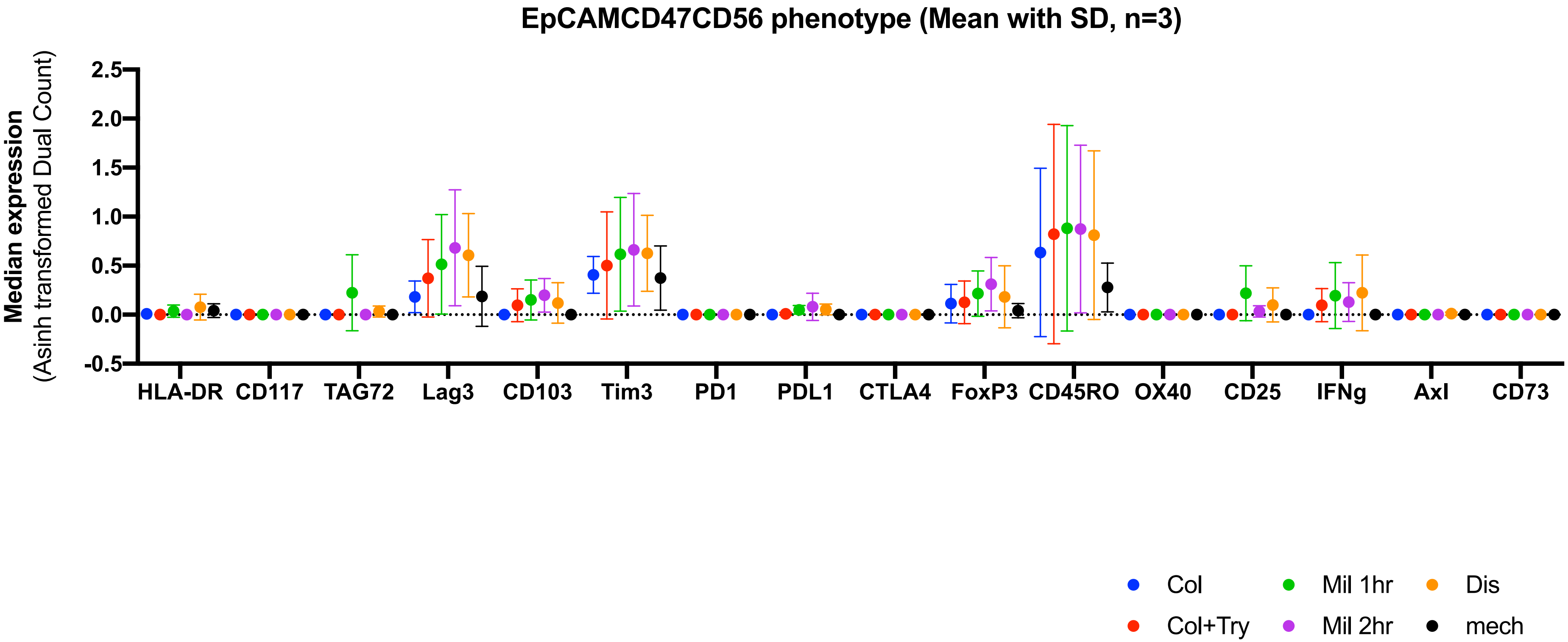
EpCAMCD47CD56 phenotype (Mean with SD, n=3)
Median expression
(Asinh transformed Dual Count)
2.5
2.0
1.5
1.0
0.5
0.0
-0.5
HLA-DR
CD117
TAG72
Lag3
CD103
Tim3
PD1
PDL1
CTLA4
FoxP3
CD45RO
OX40
CD25
IFNg
Axl
CD73
Col
Col+Try
Mil 1hr
Mil 2hr
Dis
mech

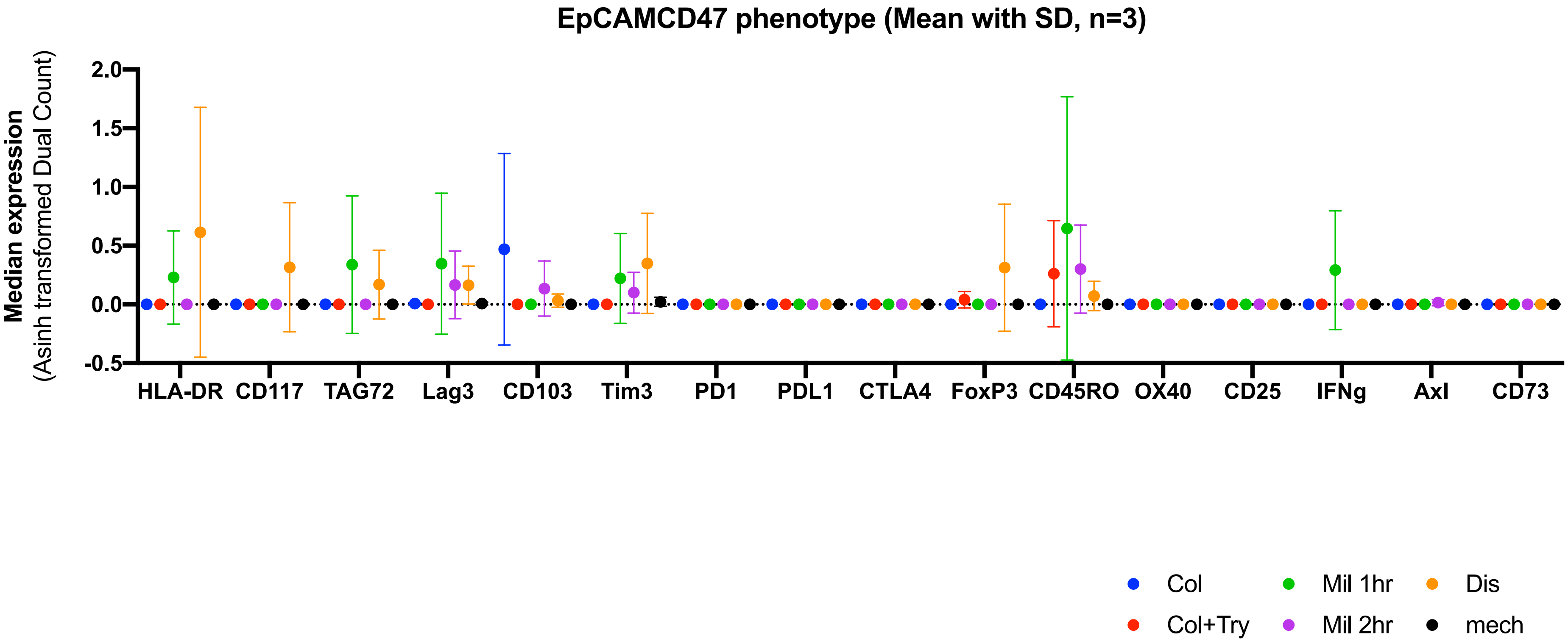

EpCAMCD47 phenotype (Mean with SD, n=3)
Median expression
(Asinh transformed Dual Count)
2.0
1.5
1.0
0.5
0.0
-0.5
HLA-DR
CD117
TAG72
Lag3
CD103
Tim3
PD1
PDL1
CTLA4
FoxP3
CD45RO
OX40
CD25
IFNg
Axl
CD73
Col
Col+Try
Mil 1hr
Mil 2hr
Dis
mech

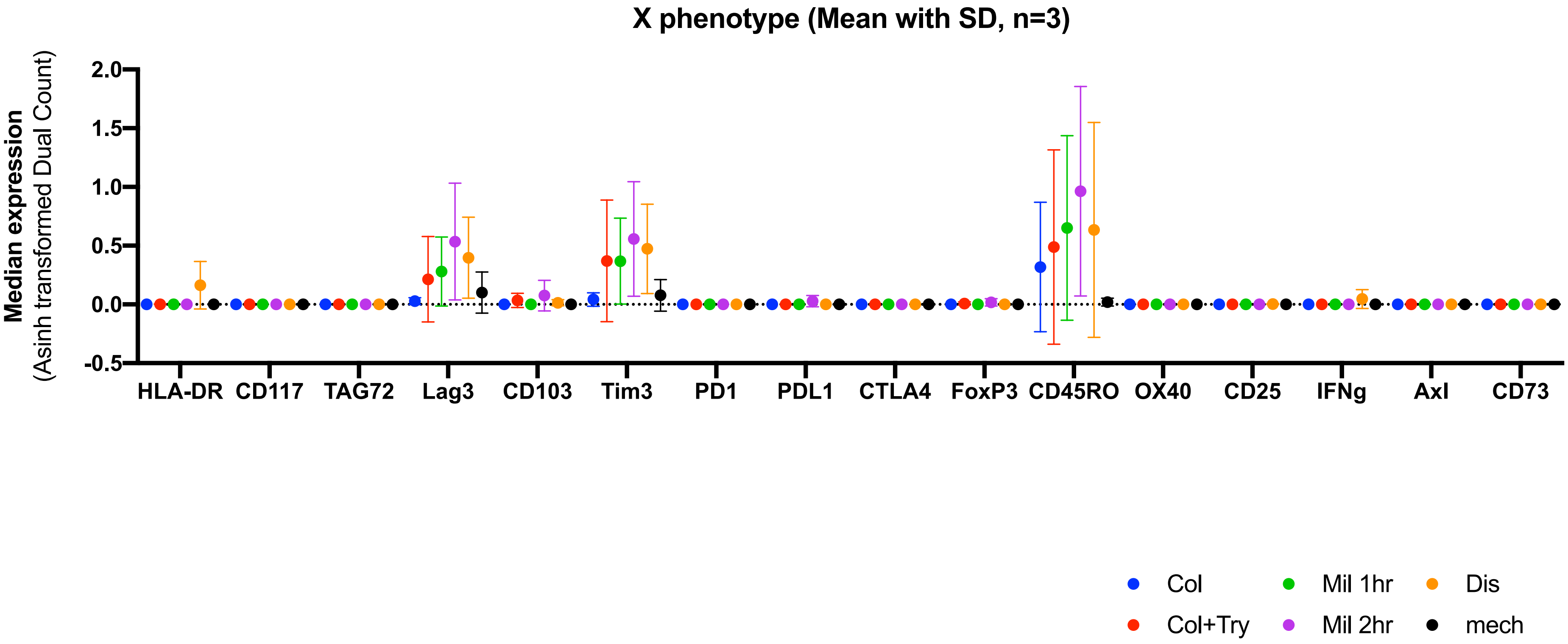
X phenotype (Mean with SD, n=3)
Median expression
(Asinh transformed Dual Count)
2.0
1.5
1.0
0.5
0.0
-0.5
HLA-DR
CD117
TAG72
Lag3
CD103
Tim3
PD1
PDL1
CTLA4
FoxP3
CD45RO
OX40
CD25
IFNg
Axl
CD73
Col
Col+Try
Mil 1hr
Mil 2hr
Dis
mech

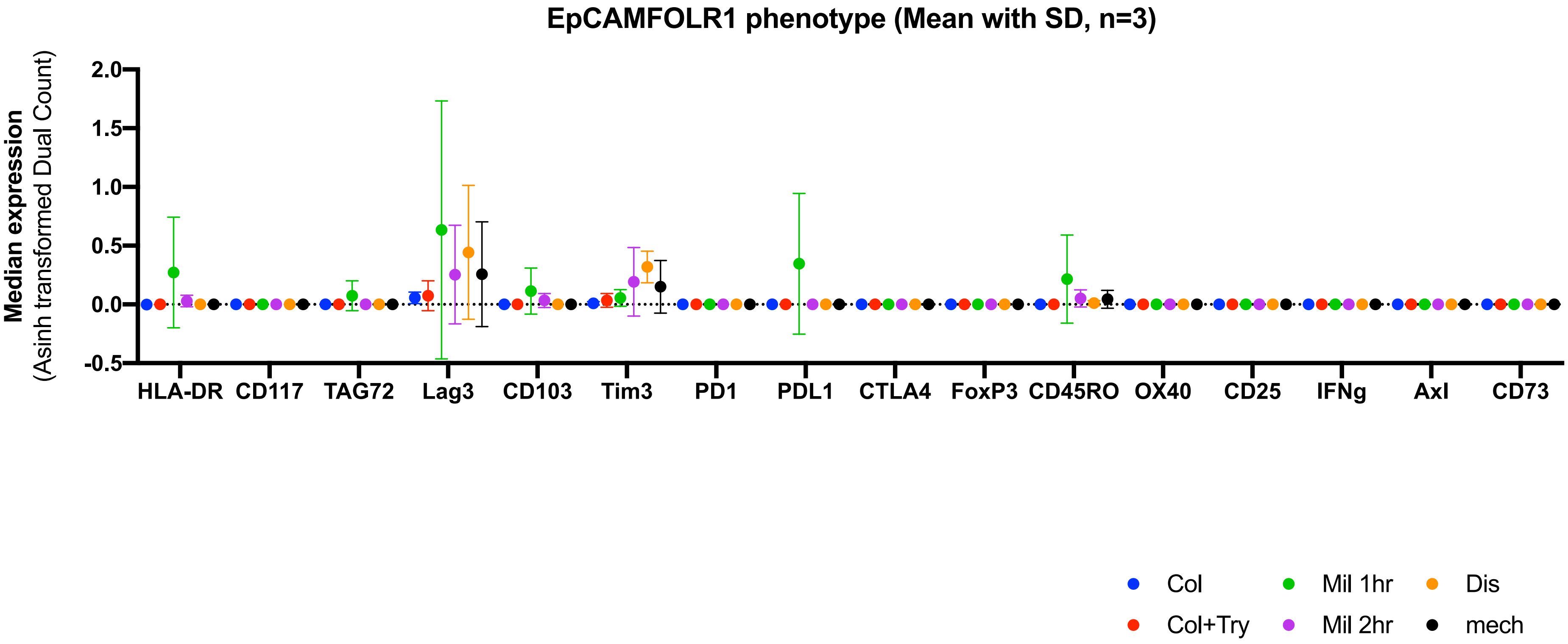

EpCAMFOLR1 phenotype (Mean with SD, n=3)
Median expression
(Asinh transformed Dual Count)
2.0
1.5
1.0
0.5
0.0
-0.5
HLA-DR
CD117
TAG72
Lag3
CD103
Tim3
PD1
PDL1
CTLA4
FoxP3
CD45RO
OX40
CD25
IFNg
Axl
CD73
Col
Col+Try
Mil 1hr
Mil 2hr
Dis
mech

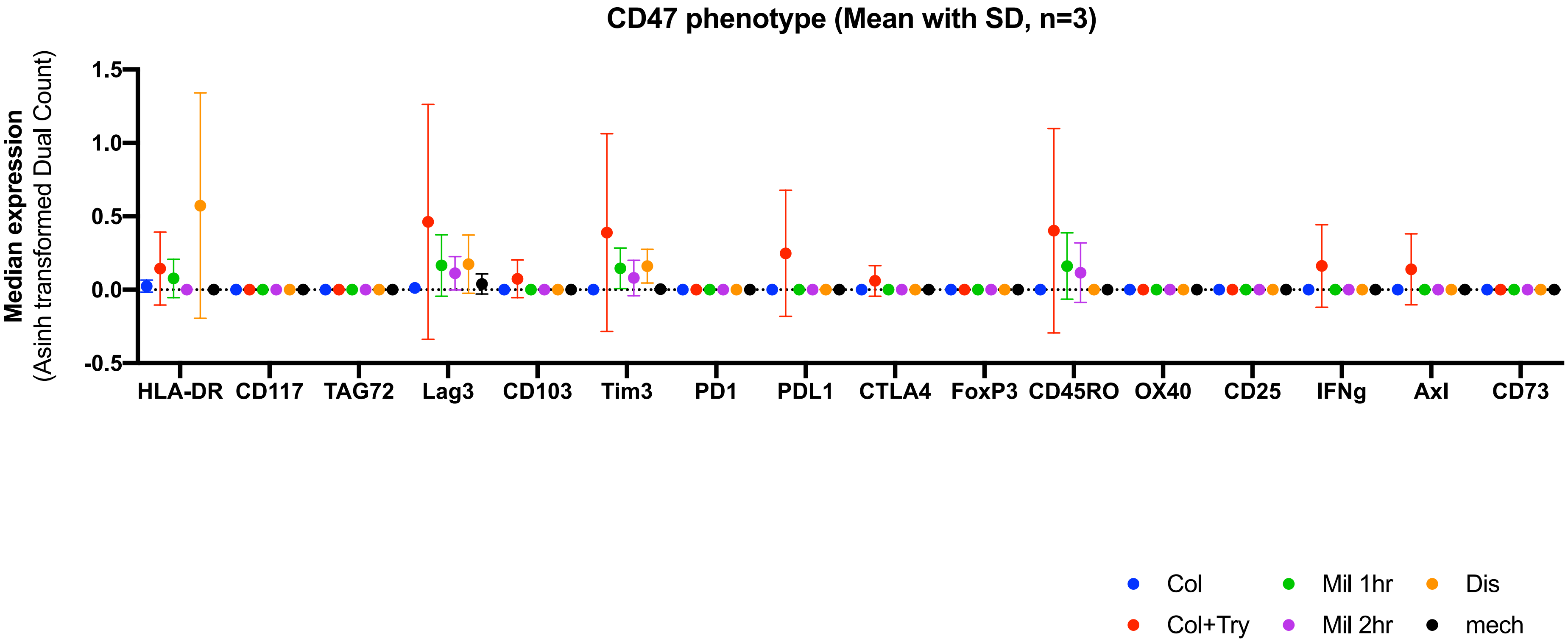

CD47 phenotype (Mean with SD, n=3)
Median expression
(Asinh transformed Dual Count)
1.5
1.0
0.5
0.0
-0.5
HLA-DR
CD117
TAG72
Lag3
CD103
Tim3
PD1
PDL1
CTLA4
FoxP3
CD45RO
OX40
CD25
IFNg
Axl
CD73
Col
Col+Try
Mil 1hr
Mil 2hr
Dis
mech

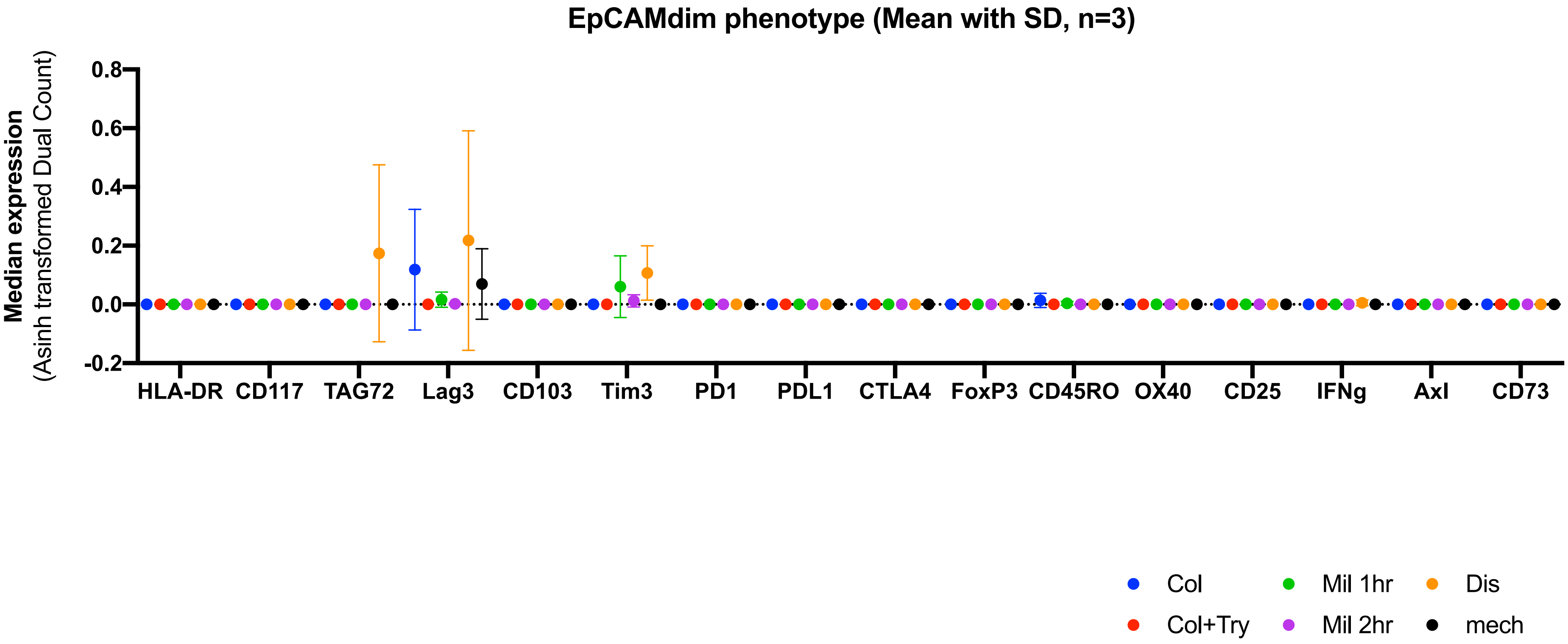
EpCAMdim phenotype (Mean with SD, n=3)
Median expression
(Asinh transformed Dual Count)
0.8
0.6
0.4
0.2
0.0
-0.2
HLA-DR
CD117
TAG72
Lag3
CD103
Tim3
PD1
PDL1
CTLA4
FoxP3
CD45RO
OX40
CD25
IFNg
Axl
CD73
Col
Col+Try
Mil 1hr
Mil 2hr
Dis
mech

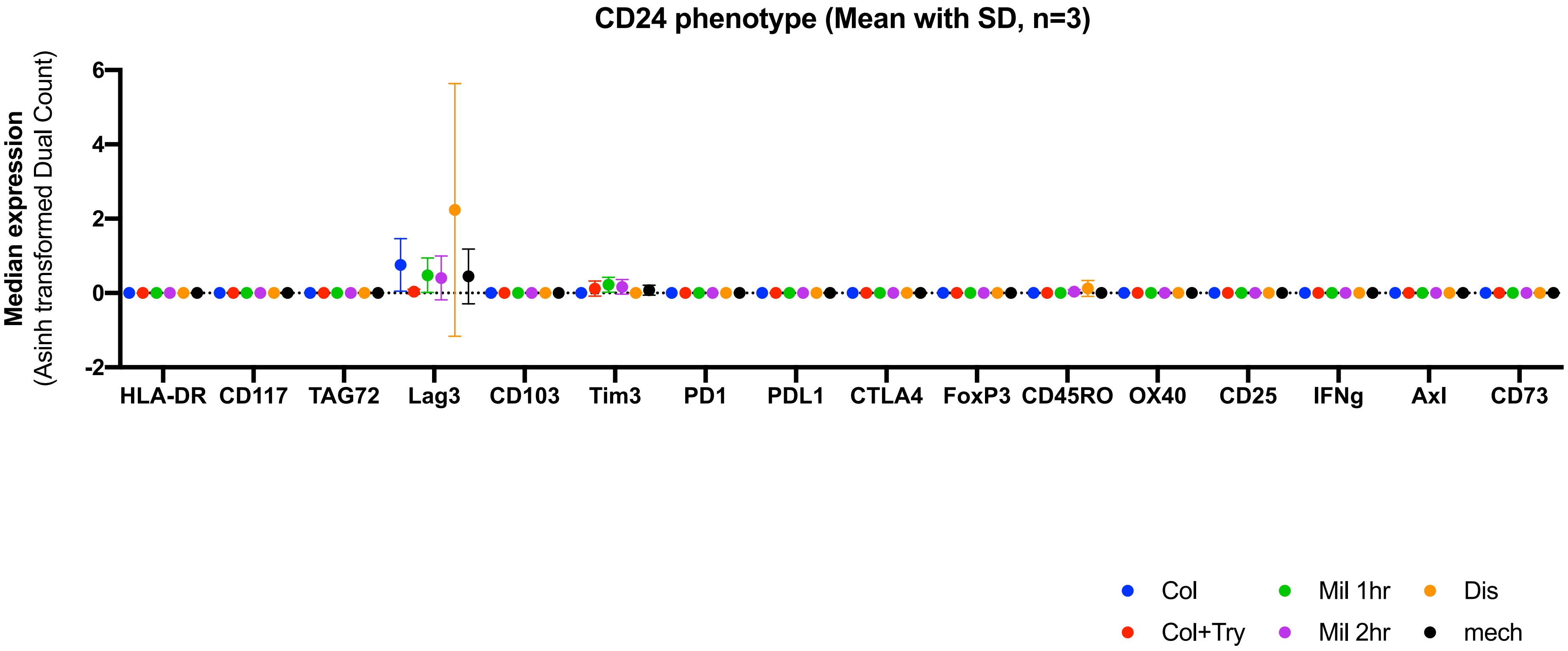
CD24 phenotype (Mean with SD, n=3)
Median expression
(Asinh transformed Dual Count)
6
4
2
0
-2
HLA-DR
CD117
TAG72
Lag3
CD103
Tim3
PD1
PDL1
CTLA4
FoxP3
CD45RO
OX40
CD25
IFNg
Axl
CD73
Col
Col+Try
Mil 1hr
Mil 2hr
Dis
mech

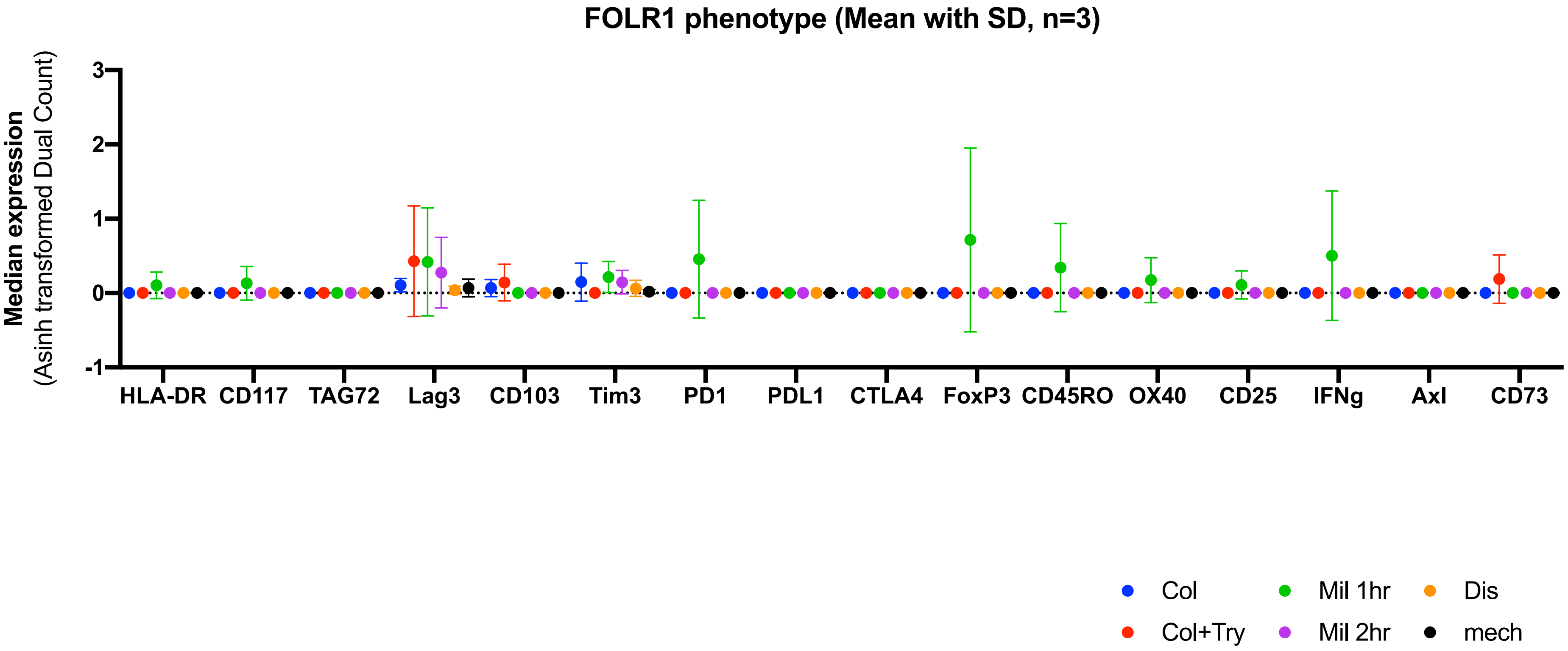
FOLR1 phenotype (Mean with SD, n=3)
Median expression
(Asinh transformed Dual Count)
3
2
1
0
-1
HLA-DR
CD117
TAG72
Lag3
CD103
Tim3
PD1
PDL1
CTLA4
FoxP3
CD45RO
OX40
CD25
IFNg
Axl
CD73
Col
Col+Try
Mil 1hr
Mil 2hr
Dis
mech
